# Nitro-sulfinate Reductive Coupling to Access (Hetero)aryl Sulfonamides

**DOI:** 10.1021/acs.joc.3c02557

**Published:** 2024-01-19

**Authors:** Sandra E. Gatarz, Oliver M. Griffiths, Henrique A. Esteves, Wenhua Jiao, Peter Morse, Ethan L. Fisher, David C. Blakemore, Steven V. Ley

**Affiliations:** † Yusuf Hamied Department of Chemistry, University of Cambridge, Cambridge CB2 1EW, U.K.; ‡ Medicine Design, Pfizer, Inc., Groton, Connecticut 06340, United States

## Abstract

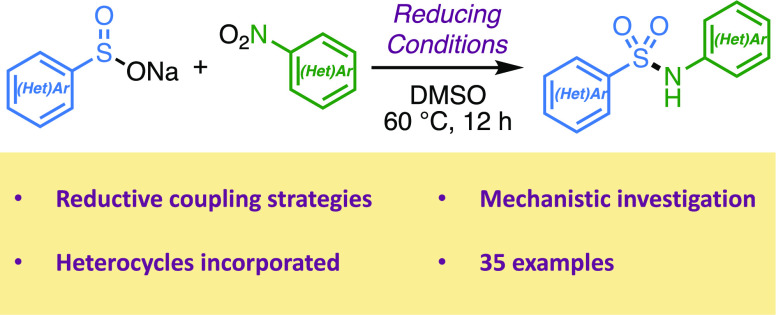

A method to assemble (hetero)aryl sulfonamides via the reductive coupling of aryl sulfinates and nitroarenes is reported. Various reducing conditions with sodium bisulfite and with or without tin(II) chloride in DMSO were developed using an ultrasound bath to improve reaction homogeneity and mixing. A range of (hetero)aryl sulfonamides bearing a selection of functional groups were prepared, and the mechanism of the transformation was investigated. These investigations have led us to propose the formation of nitrosoarene intermediates, which were established via an independent molecular coupling strategy.

## Introduction

**Figure 1:**
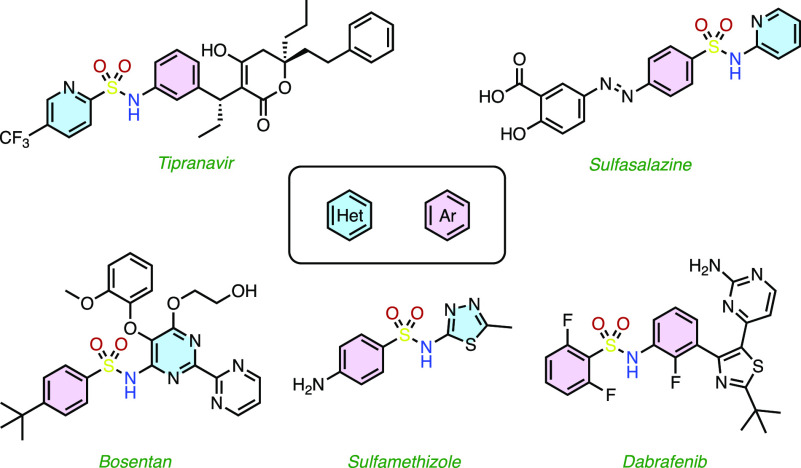
Drugs containing (hetero)aryl sulfonamides.

Aryl sulfonamides, and particularly their heteroaryl counterparts, are featured commonly in active pharmaceutical ingredients (APIs) and agrochemical agents.^1^ Their importance is exhibited in compounds such as those used in the treatment of human immunodeficiency viruses (tipranavir), rheumatoid arthritis (sulfasalazine), high blood pressure (bosentan), bacterial infections (sulfamethazole), and cancer (dabrafenib) among others (Figure 1).^1b^ Despite their noticeable absence in natural products,^2^ sulfonamides were reported in 2018 to be present in over 8% of all APIs, largely due to their advantageous properties pertaining to metabolic stability, improved physicochemical characteristics, and even their tendency to form crystalline solids, which aids workup.^3^ As a consequence, numerous methods for the synthesis of sulfonamides have emerged over the years,^4^ yet even the most common and versatile procedures, such as the base-promoted coupling of sulfonyl chlorides with amines,^4v^ are not without challenges. These can include reactivity issues with electron-withdrawn (hetero)arylamines, functional group incompatibilities, solubility issues and instability in either component, and even high toxicity of the coupling partners.^3,4v,5^ Furthermore, the chemical space accessible using this approach is limited by the lack of commercially available sulfonyl chlorides and routes to prepare them, in particular their alkyl, alkenyl, and heteroaryl counterparts.^4a,4,6^

**Scheme 1:**
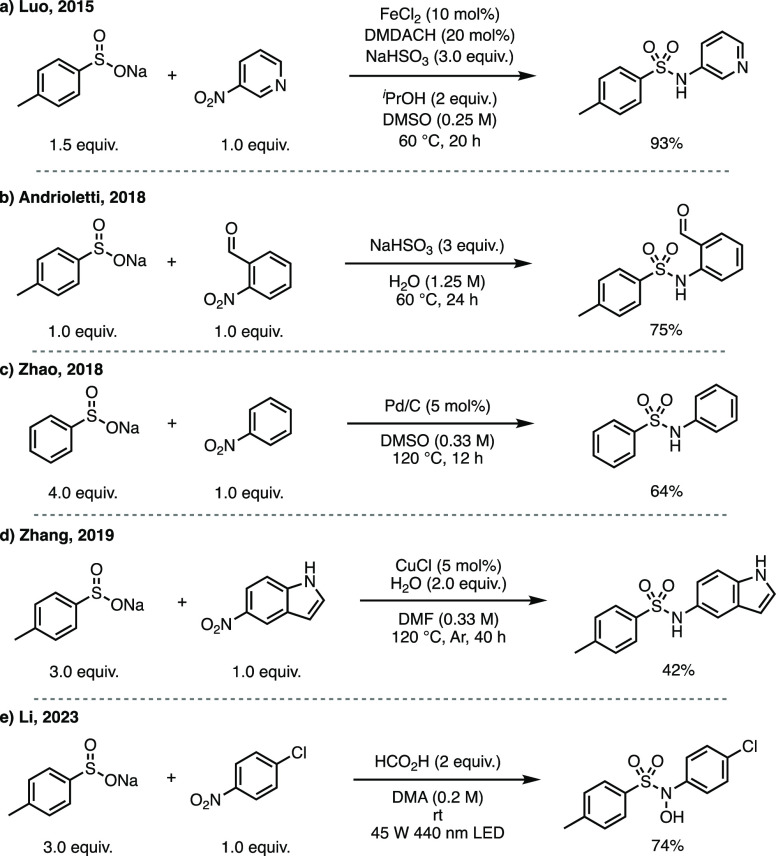
(a–e) Previously Reported Nitro-sulfinate Reductive Coupling Strategies as Alternative S–N Bond-Forming Reactions

For these reasons, synthetic chemists have expanded the tools available for the assembly of these useful entities. Indeed, an alternative S–N bond-forming strategy that has emerged is nitro-sulfinate reductive coupling (Scheme 1). This approach involves the direct coupling of nitroarenes and (typically) aryl sulfinates under reducing conditions.^7^ By employing a reductive coupling of otherwise mutually inert, readily available functional groups, there are opportunities to overcome some of the limitations associated with other sulfonamide preparations. Furthermore, nitroarenes and sodium sulfinate salts are widely available and/or readily preparable, are typically stable solids, and are among the most common intermediates.^8^ Direct coupling of these fragments bypasses the need to first prepare mutually reactive intermediates. Despite these potential advantages, nitro-sulfinate reductive couplings have so far shown practical limitations by requiring heat and/or transition metal catalysts, large stoichiometric excesses of one coupling partner, and functional group limitations, in particular with respect to aromatic heterocycles.^7^ Indeed, previous methods are limited to 2- or 3-nitropyridines and indoles as nitroheteroarene substrates.^7a,7^

Aware of these limitations, we became interested in this area and in particular the work of Luo in 2015, who showed that aryl sulfinates could be coupled with readily available nitroarenes to give the corresponding sulfonamides under reducing conditions in the presence of a catalytic quantity of FeCl_2_ and a diamine ligand.^7a^ Here, we report the development of new procedures facilitating the preparation of (hetero)aromatic sulfonamide compounds of pharmaceutical relevance.

## Results and Discussion

To begin our work, a reaction optimization study was conducted using sodium 4-fluorobenzenesulfinate (**1a**) and nitroimidazole **2a** as coupling partners under the original Luo conditions (Table 1).^7a^ The reaction proved to be capricious and failed to deliver the desired sulfonamide **3a** reliably (entry 1). However, variations on the procedure proved interesting. Without the addition of FeCl_2_, the reaction gave **3a** in 62% yield by ^19^F NMR (entry 2). It was also observed that 21% of **1a** was converted to the homocoupling byproduct **4a**. The further removal of half of the sodium bisulfite led to a reduced 35% conversion of **2a** to **3a** and also a reduction in the formation of **4a** (entry 3). Increasing the excess of sodium bisulfite to 4.5 equiv. then produced **3a** in essentially quantitative conversion, with 17% of **1a** converted to **4a** (entry 4). Interestingly, removing FeCl_2_ and exchanging NaHSO_3_ for SnCl_2_ also produced **3a** in 34% yield (entry 5), though unreacted *N*-sulfonyl hydroxylamine intermediate was observed.

**Table 1:**
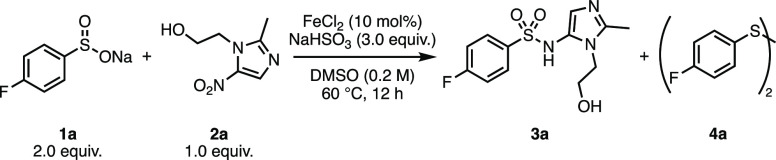
Reaction Condition Optimization^c^

entry	deviation from conditions	conversion of 2a to 3a (%)^a^	conversion of 1a to 4a (%)^a^
1	none	50	21
2	no FeCl_2_	62	21
3	no FeCl_2_, 1.5 equiv. of NaHSO_3_	35	13
4	no FeCl_2_, 4.5 equiv. of NaHSO_3_	quant.	17
5	no FeCl_2_, 3.0 equiv. of SnCl_2_ instead of NaHSO_3_	34^b^	0

a Conversion determined using ^19^F NMR and an internal standard.

b Unconverted *N*-sulfonyl hydroxylamine intermediate observed.

c Reaction conditions: 0.2 mmol, 2.0 equiv. of sodium 4-fluorobenzenesulfinate (**1a**), 1.0 equiv. of metronidazole (**2a**), 3.0 equiv. of sodium bisulfite, 10 mol % FeCl_2_ in 1 mL of DMSO (0.2 M).

With these new conditions in hand, the reaction was repeated on a 0.5 mmol scale. Unexpectedly, gelling of the reaction mixture was observed to such an extent that the reaction mixture ceased to stir effectively. This created reproducibility issues between the 0.2 and 0.5 mmol scale reactions. Recognizing that this mass transfer problem could be even more significant on ever larger scales, we decided to investigate alternative methods of stirring the reaction mixture to improve reaction homogeneity. This approach was adopted instead of simply diluting the reaction mixture further owing to product isolation difficulties from large volumes of DMSO.

The gel formation was particularly pronounced during the attempted coupling of **1a** with 5-nitro-7-azaindole (**2b**), and so, the investigation began with these substrates (Table 2). On a 0.2 mmol scale, sulfonamide **3b** was obtained in quantitative conversion. However, when scaled to 0.5 mmol, **3b** was obtained in a meager 30% yield. By recording a repeat of the 0.5 mmol reaction with a camera,^9^ we noticed a nonhomogeneous mixture formed with insolubilized solid material accumulating at the bottom of the reaction vessel. By employing an Eppendorf ThermoMixer to act as the stirrer mantle, more vigorous stirring was achieved. Indeed, a significantly improved yield of 63% of **3b** was achieved, despite the observation of an accumulated slurry at the base of the vessel. To address this, the reaction was again repeated in an ultrasonic bath,^10^ whereby gel aggregation was prevented and **3b** was obtained in 70% yield. During the optimization process, reactions were associated with a mild unpleasant odor, possibly due to the reduction of DMSO to dimethylsulfide to a small extent. However, DMSO proved to be the best solvent investigated for this reaction.

**Table 2:**
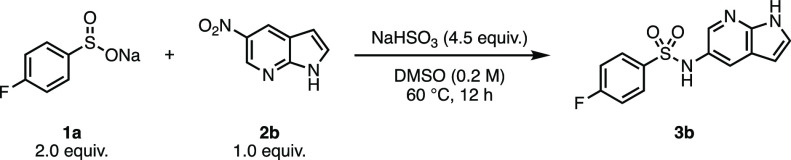
Investigating Alternative Modes of Mixing^b^

entry	stirring method	scale (mmol)	conversion to 3b (%)
1	conical stirring bar (800 rpm)	0.2	quant.
2	conical stirring bar (1100 rpm)	0.5	30%
3	Eppendorf ThermoMixer	0.5	63%
4	ultrasonic bath	0.5	70%

a Conversion determined using ^19^F NMR and an internal standard.

b Reaction conditions: 2.0 equiv. of sodium 4-fluorobenzenesulfinate (**1a**), 1.0 equiv. of 5-nitro-7-azaindole (**2b**), 4.5 equiv. of sodium bisulfite in 1 mL of DMSO (0.2 M).

With new conditions and setup for the reaction, a substrate scope was then established, beginning with varying the nitro(hetero)arene component (Table 3). Pleasingly, both *N*-substituted as well as *N*-*H*-nitropyrazoles were well-tolerated, giving **3c**, **3d**, and **3e** in 35, 32, and 34% yields, respectively. Other nitro-substituted azoles and azines were also suitable substrates. Tinidazole (**2f**) was directly converted to sulfonamide **3f** in 23% yield. 2- and 3-substituted pyridines **3g** and **3h** were prepared in 45 and 37% yields, respectively, while **3b** was isolated in 61% yield. Nitroarenes bearing various functional groups were generally well-tolerated. Sulfoxide **3i** was prepared in 41% yield. Aldehyde **3j** was isolated in 34% yield, which would otherwise encounter preparative issues using more conventional methods. Sulfonamide **3k** was isolated in 63% yield. Interestingly, amines **3l** and **3m** were also isolated in 64 and 67% yields, respectively, which would otherwise encounter selectivity issues if prepared by direct sulfonyl chloride-amine condensations.^11^ One class of substrates found to be unsuited to the reaction conditions was chloro-substituted nitroarenes, which instead of reacting in the desired reductive coupling fashion, underwent S_*N*_Ar-based reactivity with the sodium 4-fluorobenzenesulfinate nucleophile to form sulfones (see the SI).^12^

**Table 3:**
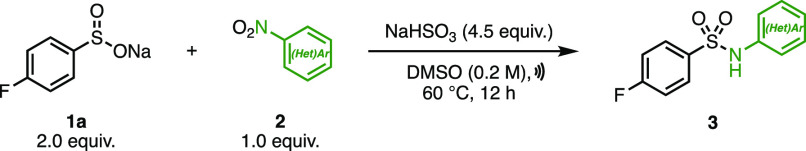

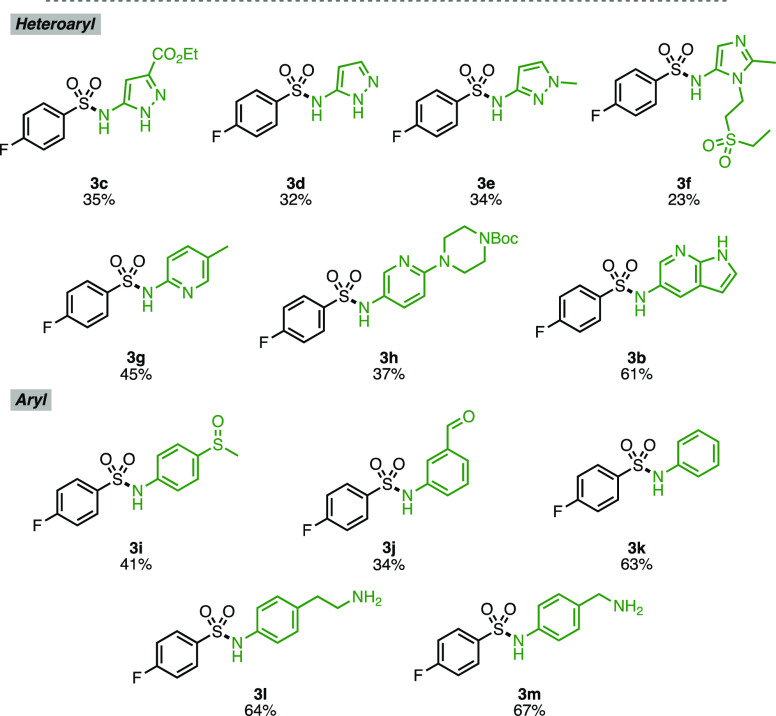
Nitro(hetero)arene Scope Investigation^a^

a Reaction conditions: 0.5 mmol, 2.0 equiv. of sodium 4-fluorobenzenesulfinate (**1a**), 1.0 equiv. of nitro(hetero)arene (**2**), 4.5 equiv. of sodium bisulfite in DMSO.

While investigating the scope of the reaction, we observed that the polar nature of many of the sulfonamides prepared resulted in some loss into the aqueous phase during the extraction process of the workup. Previous methods employed similar aqueous workups to remove DMSO from the reaction mixture and were likely to be well-suited to the compounds being investigated as they were relatively nonpolar.^7a^ As an alternative to aqueous extraction, Kugelrohr distillation was employed to remove DMSO from the crude reaction mixture. This enabled us to isolate comparatively polar compounds such as **3l** and **3m** in good yields by chromatography with the DMSO removed.

Next, we moved on to investigate the sodium (hetero)aryl sulfinate section of the scope (Table 4). The sodium sulfinates in this instance were prepared by the aqueous sodium sulfite-mediated reduction of corresponding sulfonyl chlorides.^13^ Using 1-methyl-3-nitropyrazole (**2e**) as the nitroarene, a selection of sodium aryl sulfinates were investigated. First, sodium heteroaryl sulfinates delivered sulfonamides with varying success. Thiophene sulfonamides **3n** and **3o** were isolated in modest 23 and 19% yields, respectively. Isoxazole **3p**, however, was isolated in 61% yield. In addition to heterocycles directly attached to the sulfonamide group, other heterocycles were tolerated as appendages to *S*-aryl sulfonamides, including pyrimidine (**3o**) and pyridine (**3q**). Further to these, *S*-aryl sulfonamides bearing various substituents were also obtained. Electron-donating groups were well-tolerated with **3s** and **3t** isolated in 62 and 55% yields, respectively. Sulfone- and methyl-substituted aryl sulfonamides **3u** and **3v** were isolated in 56 and 57% yields. Sodium aryl sulfinates bearing electron-withdrawing groups also delivered their corresponding sulfonamides (**3w** and **3x**) in moderate yields.

**Table 4:**
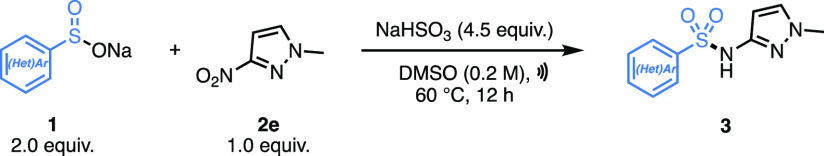

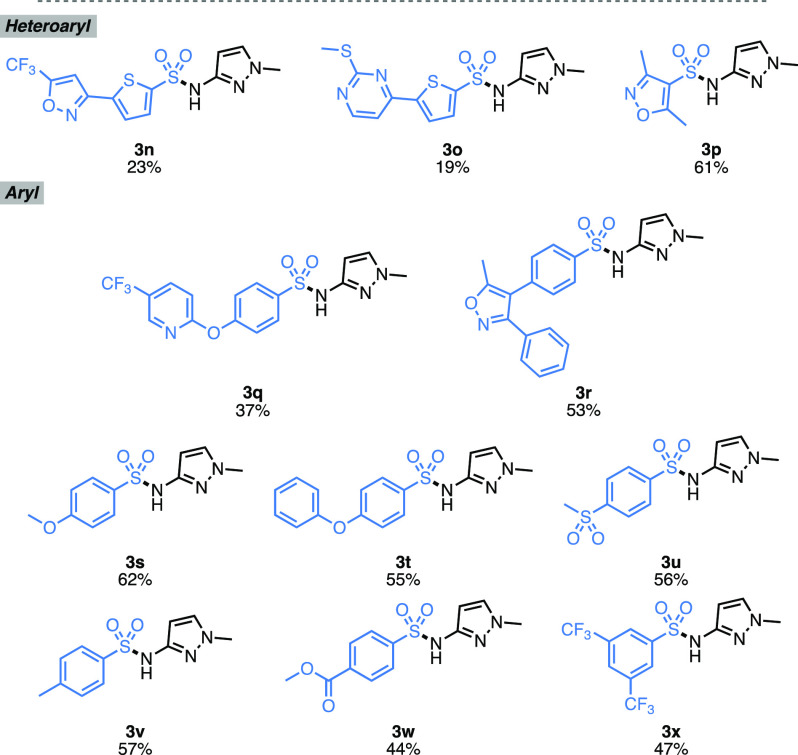
Sodium (Hetero)aryl Sulfinate Scope Investigation^a^

a Reaction conditions: 0.5 mmol, 2.0 equiv. of sodium (hetero)aryl sulfinate (**1**), 1.0 equiv. of 1-methyl-3-nitropyrazole (**2e**), 4.5 equiv. of sodium bisulfite in DMSO.

Having demonstrated that various aromatic heterocycles bearing a range of appendages were amenable to the reductive coupling conditions, we anticipated that a suitable reductive coupling methodology could find applications in drug discovery as well as route scouting toward sulfonamide targets. Having addressed methods to improve the reliability of scaling the nitro-sulfinate reductive coupling, we envisioned that a library of druglike sulfonamides could be generated from the corresponding sodium (hetero)aryl sulfinates and nitro(hetero)arenes.^14^ The building blocks containing toxicophoric and highly reactive groups were excluded.^15^ Initially, the library was enumerated automatically in DataWarrior to generate 144 (12 × 12) virtual compounds.^16^ Of this library, seven members (**3y**–**3ae**) were prepared in the laboratory using the aforementioned reductive coupling strategy (Table 5).

**Table 5:**
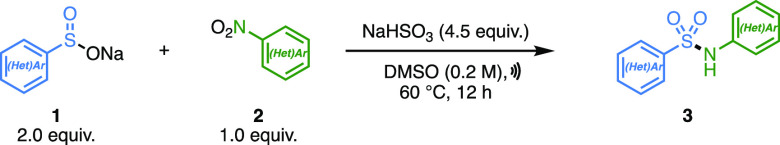

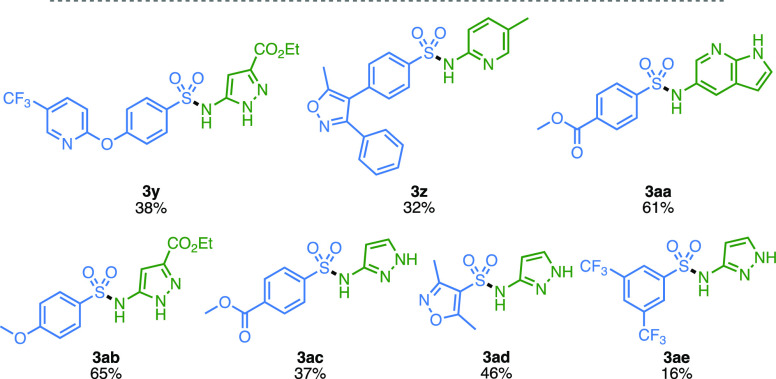
Druglike Sulfonamides^a^

a Reaction conditions: 0.5 mmol, 2.0 equiv. of sodium (hetero)aryl sulfinate (**1**), 1.0 equiv. of 1-methyl-3-nitropyrazole (**2e**), 4.5 equiv. of sodium bisulfite in DMSO.

**Scheme 2:**
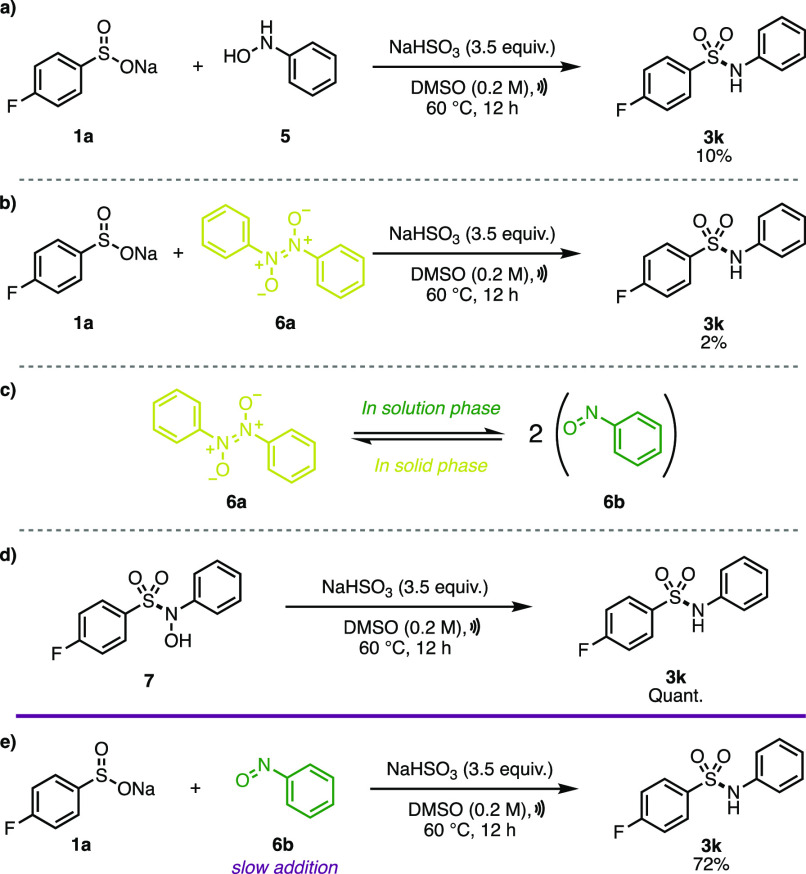
a) Control Experiment Using *N*-Phenyl Hydroxylamine (**5**), (b) Control Experiment Using Azobenzene Dioxide (**6a**), (c) Dissolution-Influenced Equilibrium of Azobenzene Dioxide **6a** and Nitrosobenzene (**6b**), (d) Sodium Bisulfite-Mediated Reduction of *N*-Sulfonyl Hydroxylamine **7**, and (e) Control Experiment Using a Slowly Added Solution of Nitrosobenzene (**6b**)

Previous investigations into the mechanisms of nitro-sulfinate reductive couplings had not provided conclusive evidence for key proposed intermediates during the reaction, with the exception of the formation of *N*-hydroxyl sulfonamide intermediates, which can be isolated during incomplete reductions.^7a,7e^ We then began our own preliminary set of control experiments (Scheme 2, see the SI for full details). Under the reaction conditions, we envisioned that potential intermediates would be *N*-arylhydroxylamines (**5**) or nitrosoarenes (**6**), as was previously suggested by Luo before they concluded otherwise as a result of their own control experiments.^7a^ This is because these intermediates are known to be formed during the bisulfite-mediated reduction of nitroarenes, as in the Piria reaction.^17^ As was consistent with Luo, when we subjected each of these intermediates directly into the standard reaction conditions with **1a**, we observed the formation of only small amounts of sulfonamide **3k** (Scheme 2a,b).^7a^ However, we were not convinced that this ruled out the formation of either **5** or **6b** as intermediates for two reasons. First, *C*-nitroso compounds typically exist as azobenzene dioxide dimers (**6a**) in the solid state (though can be isolated as green solid monomers upon sublimation), and so, the proposed electrophilic nitroso species **6b** is only formed upon dissociation of the dimer, which is often achieved in solution (Scheme 2c).^18^ Indeed, this equilibrium needs to be carefully controlled to use nitroso compounds effectively in synthesis.^19^ As a result, we speculated that addition of solid dimer **6a** in one go may prevent the reaction from proceeding due to a lack of dimer dissociation, as monomeric **6b** was the intermediate in the reaction. Second, *C*-nitroso arenes are known to react with sodium aryl sulfinates to form *N*-sulfonyl hydroxylamines, such as **7**.^20^ Indeed, we had isolated *N*-sulfonyl hydroxylamines during the optimization of the reaction when insufficient quantities of reducing agent were added. We also demonstrated that pure **7** could be reduced under the reaction conditions to **3k** in quantitative yield (Scheme 2d). This is consistent with other works, where *N*-sulfonyl hydroxylamines have been shown to reduce to the corresponding sulfonamide with sodium bisulfite.^7a^ With this information at hand, a 0.2 M solution of **6b** in DMSO-*d*_6_ was prepared. This solution was shown by ^1^H NMR to be in the monomeric form that was also the characteristic deep blue-green color typical of *C*-nitroso monomers (see the SI). This solution was then added slowly over the period of 1 h to a reaction mixture containing **1a** and sodium bisulfite in DMSO-*d*_6_ heated at 60 °C (Scheme 2e). Gratifyingly, sulfonamide **3k** was observed to form in 72% yield by ^19^F NMR. We see this as supporting evidence for potential formation of the nitrosoarene monomer (**6b**) as a key intermediate in the reaction.

**Scheme 3:**
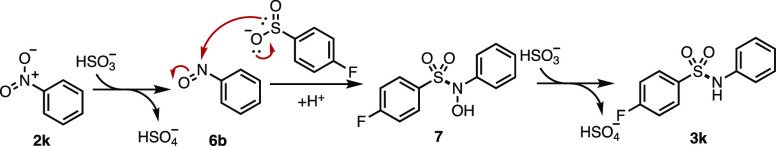
Proposed Reaction Mechanism

With these control experiments, we suggest that the reaction proceeds via nitrosoarene and *N*-sulfonyl hydroxylamine intermediates (Scheme 3). First, the nitroarene starting material (**2k**) undergoes a 2-electron reduction, in an analogous fashion to the Piria reaction,^16^ to a nitroso monomer intermediate (**6**), which upon electrophilic interception of the sodium aryl sulfinate nucleophile (**1a**) and protonation forms an *N*-sulfonyl hydroxylamine (**7**). Subsequent 2-electron reduction of this species then produces the desired sulfonamide **3k**. Further studies into the mechanism are needed, however, to confirm whether the reaction proceeds via **7** or its conjugate base under these reaction conditions.

With additional insight into the mechanism of the sodium bisulfite-mediated reductive coupling reaction, we reasoned that a wider range of nitroarenes may become viable substrates with the choice of other reducing agents as the first step of the proposed mechanism requires direct reduction of the nitroarene to the nitrosoarene. After a quick screen with alternative reducing agents for previously sluggish/unreactive substrates such as **2af**, we found that a combination of sodium bisulfite and tin(II) chloride was successfully able to convert them (entry 2, Table 6). For example, sodium 4-fluorobenzenesulfinate (**1a**) was reductively coupled with nitroheteroarene **2af** in 81% conversion.

**Table 6:**
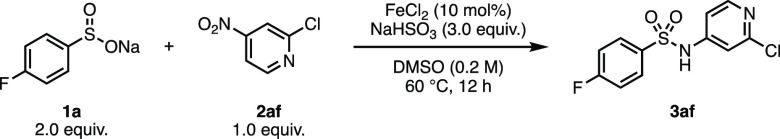
Alternative Reducing Agent Investigation^c^

entry	deviation from conditions	conversion to 3af (%)^a^
1	none	1
2	1.5 equiv. of SnCl_2_ added	81
3	no FeCl_2_, 1.5 equiv. of SnCl_2_ added	quant.
4	no FeCl_2_, 1.5 equiv. of SnCl_2_ added, 1.5 equiv. of NaHSO_3_	89^b^

a Conversion determined using ^19^F NMR and an internal standard.

b Unconverted *N*-sulfonyl hydroxylamine intermediate observed.

c Reaction conditions: 0.2 mmol, 2.0 equiv. of sodium 4-fluorobenzenesulfinate (**1a**), 1.0 equiv. of nitroarene **2af**, 3.0 equiv. of sodium bisulfite, in DMSO (0.2 M).

After a brief optimization, we found that 1.5 equiv. of tin(II) chloride and 3 equiv. of sodium bisulfite produced **3af** in quantitative conversion (entry 3). Interestingly, both reducing agents were required to achieve full conversion. Reduction in the quantity of sodium bisulfite resulted in incomplete conversion of the *N*-sulfonyl hydroxylamine intermediate (entry 4). With these new conditions, we prepared a small selection of sulfonamides, many of which were sluggish under the original conditions, including **3ag**–**3aj** in moderate to very good yields (Table 7). Not only this, but arenes that previously underwent S_*N*_Ar solely with sodium bisulfite as the reducing agent instead selectively formed the desired sulfonamide with the new tin(II) chloride-sodium bisulfite conditions (**3af**, **3ah**, and **3ai**). These results indicate that the selection of particular reducing agents may enable the activation of a much wider range of nitro-heteroarenes for the reductive coupling strategy to sulfonamides.

**Table 7:**
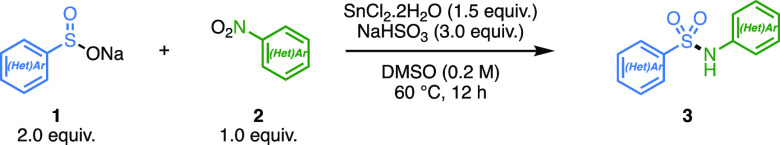

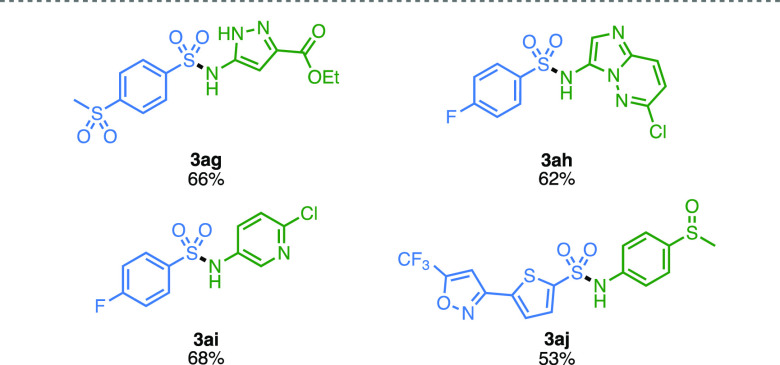
Substrates Prepared Using SnCl_2_ as a Coreducing Agent^a^

a Reaction conditions: 0.5 mmol, 2.0 equiv. of sodium (hetero)aryl sulfinate (**1**), 1.0 equiv. of nitro(hetero)arene (**2**), 1.5 equiv. of sodium bisulfite, 1.5 equiv. of SnCl_2_·2H_2_O in DMSO.

In summary, we have established a method for the reductive coupling of various nitro-heteroarenes with aryl sulfinates using sodium bisulfite with and without SnCl_2_ to afford the corresponding sulfonamides. The ready availability and stability of the initial building blocks provide a useful alternative to conventional sulfonamide preparations and in particular for the generation of heteroaryl derivatives.

## Experimental Section

### General Information

All procedures below were conducted under an inert nitrogen atmosphere unless stated otherwise. Reagents not prepared were supplied by Sigma-Aldrich, Alfa Aesar, Acros Organics, TCI, and Fluorochem and were used as received. Extra dry DMSO was purchased from Acros Organics and used for all reductive coupling reactions. Reactions carried out on a 0.5 mmol scale were done in a Fisher Scientific FB15051 ultrasonic bath. Workup solvents were obtained from commercial sources and distilled prior to use. Petroleum ether refers to the fractions of petrol collected between 40 and 60 °C bp. Automated flash column chromatography was performed using a Teledyne ISCO CombiFlash NextGen 100 system with single-use disposable silica columns of the appropriate size (SiliaSep Flash cartridges, 12 or 25 g, 40–60 μm, ISO04/012). Thin-layer chromatography (TLC) analysis was carried out using silica gel 60 F254 precoated glass-backed plates and visualized under UV light (254 nm) or with permanganate stains. ^1^H NMR, ^13^C{^1^H} NMR, and ^19^F{^1^H} NMR were obtained using a Bruker AV400 (Avance 400 MHz) spectrometer. ^1^H NMR chemical shifts (δ) are referenced to residual CHCl_3_ (7.26 ppm) and DMSO (2.50 and 3.33 ppm) in the unit of parts per million (ppm). ^13^C{^1^H} NMR chemical shifts (δ) are referenced to residual CDCl_3_ (77.5 ppm) and DMSO-*d*_6_ (39.5 ppm). Coupling constants *J* are quoted in the unit of hertz (Hz). Proton and carbon multiplicities are recorded as singlet (s), doublet (d), doublet of doublets (dd), triplet (t), quartet (q), multiplet (m), and broad (br). ^1^H NMR signals are reported to two decimal places,^13^C{^1^H} NMR signals to one decimal place, and ^19^F{^1^H} NMR signals to one decimal place. All compounds examined were dried *in vacuo* to remove residual solvents. High-resolution mass spectra (HRMS) were obtained on a Waters Xevo G2-S benchtop quadrupole time-of-flight (QTOF) spectrometer. Infrared spectra were recorded neat on a Bruker Alpha II Fourier transform infrared (FTIR) spectrometer with a universal attenuated total reflection (ATR) sampling accessory, and selected peaks were reported. The following abbreviations are used when describing the data: w (weak), m (medium), and s (strong). Melting points were uncorrected and recorded using one-end closed glass capillaries supplied by Marienfeld Superior on a Stuart Scientific melting point apparatus.

### Preparation of Starting Materials

All sodium aryl sulfinates were prepared according to the literature procedure.^12^

#### 6-Chloro-3-nitroimidazo[1,2-*b*]pyridazine (**2ah**)

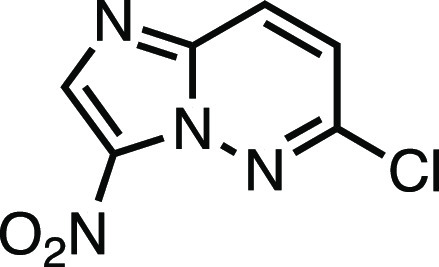



The title compound was prepared according to a literature procedure.^21^ 6-Chloro-imidazo[1,2-*b*]pyridazine (2.48 g, 15.8 mmol) was dissolved in 98% H_2_SO_4_ (30 mL) and cooled (0 °C). Concentrated HNO_3_ (5 mL) was added dropwise over 10 min with vigorous stirring. After stirring at 0 °C for 30 min, the solution was warmed to room temperature and stirred for a further 4 h. The reaction mixture was then poured onto ice (100 g), neutralized with 50% aqueous NaOH (50 mL), and then extracted with EtOAc (3 × 200 mL). The combined organic layers were washed with water (2 × 100 mL) and brine (100 mL), then dried (MgSO_4_), and filtered, and the solvent was removed *in vacuo* to produce **2ah** as a yellow crystalline solid (2.99 g, 95%). ^1^H NMR (500 MHz, CDCl_3_): δ 8.59 (s, 1H), 8.12 (d, *J* = 9.5 Hz, 1H), 7.46 (d, *J* = 9.5 Hz, 1H). ^13^C{^1^H} NMR (101 MHz, CDCl_3_): δ 150.3, 139.4, 136.5, 128.4, 124.0. mp 208–210 °C. HRMS: *m*/*z* calculated for C_6_H_4_N_4_O_2_Cl^+^, 199.0017 [M + H]^+^. Found *m*/*z*, 199.0018, Δ = 0.3 ppm. Data were consistent with those in the literature.^20^

#### 4-Fluoro-*N*-hydroxy-*N*-phenylbenzenesulfonamide (**7**)

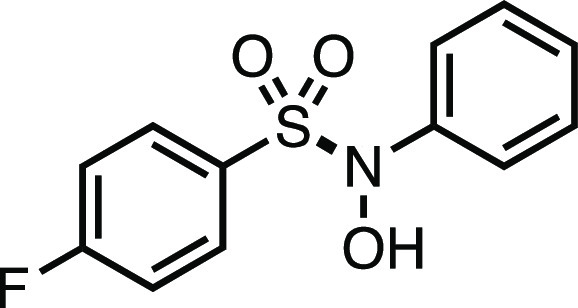



The title compound was prepared according to a literature procedure.^22^ A mixture of phenylhydroxylamine (109 mg, 1.0 mmol), NaHCO_3_ (200 mg, 1.2 mmol), and 4-fluorobenzenesulfonyl chloride (464 mg, 1.2 mmol) in THF (10 mL) was stirred at room temperature for 2 h. Water (10 mL) was added, and the mixture was extracted with EtOAc (3 × 30 mL). The combined organic layers were dried over Na_2_SO_4_, filtered, and concentrated under reduced pressure. The crude product was purified by flash column chromatography (10% EtOAc in petroleum ether) to give **7** as a beige solid (79 mg, 30%). ^1^H NMR (400 MHz, DMSO-*d*_6_): δ 11.17 (s, 1H), 7.53–7.44 (m, 2H), 7.43–7.34 (m, 2H), 7.34–7.21 (m, 3H), 7.11–7.02 (m, 2H). ^13^C{^1^H} NMR (101 MHz, DMSO-*d*_6_): δ 165.22 (d, *J* = 253.2 Hz), 142.4, 132.2 (d, *J* = 10.0 Hz), 128.6 (d, *J* = 3.0 Hz), 128.4, 127.2, 122.7, 116.1 (d, *J* = 23.0 Hz). ^19^F{^1^H} NMR (376 MHz, DMSO-*d*_6_): δ −105.1. mp 138–139 °C. ν_max_: 3346, 1344, 1148 cm^–1^. HRMS: *m*/*z* calculated for C_12_H_11_NO_3_SF^+^, 268.0444 [M + H]^+^. Found *m*/*z*, 268.0433, Δ = −4.1 ppm. Data were consistent with those in the literature.^21^

### General Procedures for the Synthesis of Sulfonamides

#### General Procedure 1

A mixture of sodium aryl sulfinate (1.0 mmol, 2.0 equiv), nitroarene (0.5 mmol, 1.0 equiv), and NaHSO_3_ (234 mg, 2.25 mmol, 3.5 equiv) in DMSO (2.5 mL, 0.2 M) was allowed to react in an ultrasonic water bath at 60 °C for 12 h. After cooling to room temperature, water (25 mL) was added, and the mixture was extracted with CH_2_Cl_2_ (3 × 40 mL). The combined organic extracts were dried over with anhydrous Na_2_SO_4_, filtered, and concentrated under reduced pressure. The crude product was purified by flash column chromatography to give the product.

#### General Procedure 2

A mixture of sodium aryl sulfinate (1.0 mmol, 2.0 equiv), nitroarene hydrochloride (0.5 mmol, 1.0 equiv), and NaHSO_3_ (234 mg, 2.25 mmol, 3.5 equiv) in DMSO (2.5 mL, 0.2 M) was allowed to react in an ultrasonic water bath at 60 °C for 12 h. The reaction was cooled down to room temperature. DMSO was removed by Kugelrohr distillation (60–80 °C, 0.1 mbar, 3 h). The crude product was purified by flash column chromatography to give the product as a free amine.

#### General Procedure 3

To a 10 mL microwave vial were added a stirrer bar, nitroarene (0.4 mmol, 1.0 equiv), aryl sulfinate (0.8 mmol, 2.0 equiv), tin chloride (0.6 mmol, 1.5 equiv), and sodium bisulfite (0.6 mmol, 1.5 equiv). The vial was then sealed, evacuated, and backfilled with nitrogen (×3). Dry DMSO (2 mL, 0.2 M) was then added, and the reaction mixture was stirred vigorously (>800 rpm) for 5 min. The mixture was then heated to 60 °C in an oil bath with vigorous stirring for 16 h. As the reaction reached a constant temperature, all components became solubilized. After 16 h, the reaction mixture was cooled to room temperature before being diluted with DCM (30 mL) and water (30 mL). The layers were separated, and the organic phase was washed with water (2 × 30 mL) and then brine (30 mL) before being dried (MgSO_4_) and filtered, and the solvent was removed *in vacuo*. The residue was then purified by column chromatography to yield the desired sulfonamide.

### Characterization of Products

#### 4-Fluoro-*N*-(1*H*-pyrrolo[2,3-*b*]pyridin-5-yl)benzenesulfonamide (**3b**)

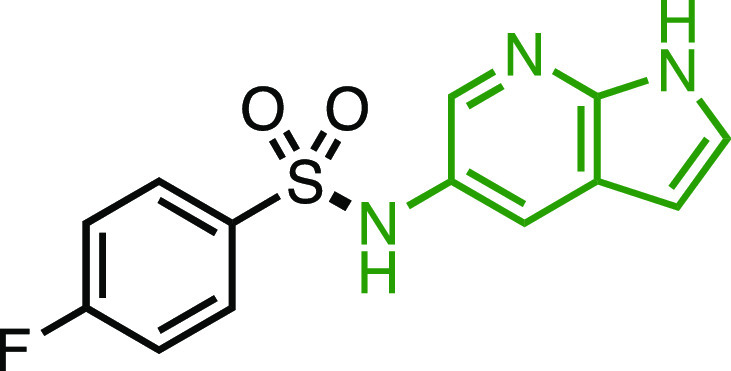



Following General Procedure 1, purification by flash column chromatography (50–80% EtOAc in petroleum ether) yielded **3b** as a white solid (88 mg, 61%). ^1^H NMR (400 MHz, DMSO-*d*_6_): δ 11.62 (s, 1H), 10.02 (s, 1H), 7.83–7.78 (m, 1H), 7.72–7.64 (m, 2H), 7.61–7.56 (m, 1H), 7.45–7.40 (m, 1H), 7.37–7.29 (m, 2H), 6.36 (dd, *J* = 3.4, 1.9 Hz, 1H). ^13^C{^1^H} NMR (101 MHz, DMSO-*d*_6_): δ 164.2 (d, *J* = 251.4 Hz), 146.5, 138.8, 135.5 (d, *J* = 3.0 Hz), 129.8 (d, *J* = 9.6 Hz), 127.4, 126.2, 123.2, 119.4, 116.4 (d, *J* = 22.7 Hz), 100.0. ^19^F{^1^H} NMR (376 MHz, DMSO-*d*_6_): δ −107.2. mp 183–185 °C. ν_max_: 1333, 1166 cm^–1^. HRMS: *m*/*z* calculated for C_13_H_11_N_3_O_2_SF^+^, 292.0556 [M + H]^+^. Found *m*/*z*, 292.0561, Δ = 1.7 ppm.

#### Ethyl 5-((4-Fluorophenyl)sulfonamido)-1*H*-pyrazole-3-carboxylate (**3c**)

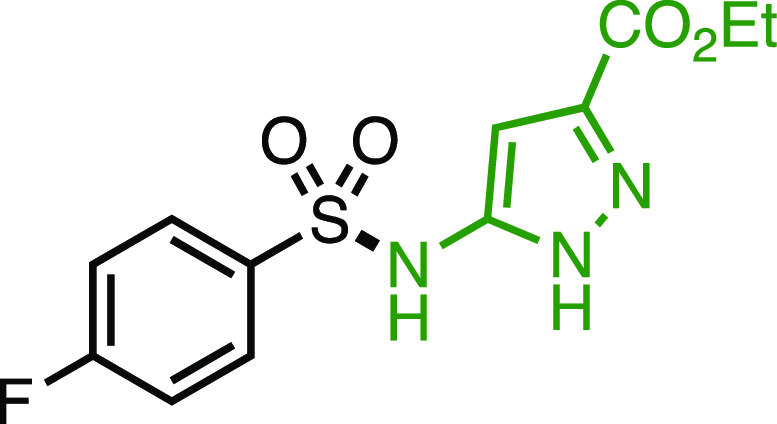



Following General Procedure 1, purification by flash column chromatography (30–50% EtOAc in petroleum ether) yielded **3c** as a white solid (55 mg, 35%). ^1^H NMR (400 MHz, DMSO-*d*_6_): δ 13.61 (s, 1H), 10.76 (s, 1H), 7.82 (dd, *J* = 8.7, 5.2 Hz, 2H), 7.46–7.35 (m, 2H), 6.46 (s, 1H), 4.27 (q, *J* = 7.1 Hz, 2H), 1.27 (t, *J* = 7.1 Hz, 3H). ^13^C{^1^H} NMR (101 MHz, DMSO-*d*_6_): δ 164.3 (d, *J* = 251.6 Hz), 158.5, 146.2, 136.3, 133.8, 129.7 (d, *J* = 9.7 Hz), 116.4 (d, *J* = 22.5 Hz), 100.3, 60.9, 14.1. ^19^F{^1^H} NMR (376 MHz, DMSO-*d*_6_): δ −107.1. mp 175–177 °C. ν_max_: 1703, 1356, 1138 cm^–1^. HRMS: *m*/*z* calculated for C_12_H_13_N_3_O_4_SF^+^, 314.0611 [M + H]^+^. Found *m*/*z*, 314.0613, Δ = −0.6 ppm.

#### 4-Fluoro-*N*-(1*H*-pyrazol-3-yl)benzenesulfonamide (**3d**)

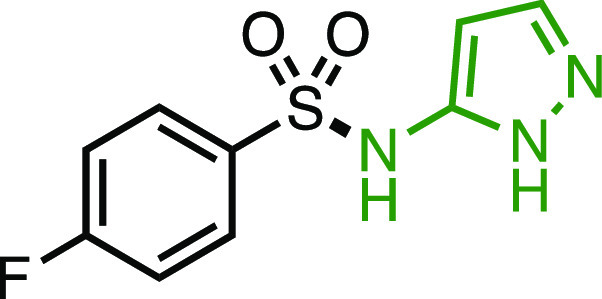



Following General Procedure 1, purification by flash column chromatography (30–90% EtOAc in petroleum ether) yielded **3d** as a white solid (38 mg, 32%). ^1^H NMR (400 MHz, DMSO-*d*_6_): δ 12.36 (s, 1H), 10.42 (s, 1H), 7.91–7.76 (m, 2H), 7.63–7.49 (m, 1H), 7.46–7.32 (m, 2H), 6.02–5.88 (m, 1H). ^13^C{^1^H} NMR (101 MHz, DMSO-*d*_6_): δ 164.19 (d, *J* = 251.0 Hz), 136.6, 129.73 (d, *J* = 9.6 Hz), 129.5, 116.16 (d, *J* = 22.9 Hz), 97.2, one quaternary carbon not visible. ^19^F{^1^H} NMR (376 MHz, DMSO-*d*_6_): δ −107.6. ν_max_: 3323, 1352, 1149 cm^–1^. HRMS: *m*/*z* calculated for C_9_H_9_N_3_O_2_SF^+^, 242.0400 [M + H]^+^. Found *m*/*z*, 242.0407, Δ = 2.9 ppm.

#### 4-Fluoro-*N*-(1-methyl-1*H*-pyrazol-3-yl)benzenesulfonamide (**3e**)

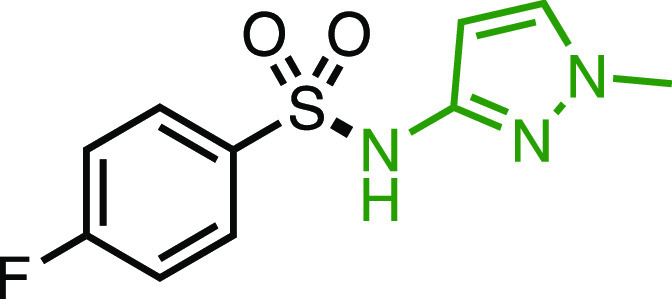



Following General Procedure 1, purification by flash column chromatography (20–90% EtOAc in petroleum ether) yielded **3e** as a white solid (43 mg, 34%). ^1^H NMR (400 MHz, DMSO-*d*_6_): δ 10.48 (s, 1H), 7.87–7.79 (m, 2H), 7.49 (d, *J* = 2.3 Hz, 1H), 7.44–7.35 (m, 2H), 5.91 (d, *J* = 2.3 Hz, 1H), 3.63 (s, 3H). ^13^C{^1^H} NMR (101 MHz, DMSO-*d*_6_): δ 164.2 (d, *J* = 251.1 Hz), 145.0, 136.5 (d, *J* = 3.0 Hz), 131.8, 129.7 (d, *J* = 9.5 Hz), 116.2 (d, *J* = 22.9 Hz), 97.3, 38.4. ^19^F{^1^H} NMR (376 MHz, DMSO-*d*_6_): δ −106.5. mp 150–151 °C. ν_max_: 1325, 1169 cm^–1^. HRMS: *m*/*z* calculated for C_10_H_11_N_3_O_2_SF^+^, 256.0556 [M + H]^+^. Found *m*/*z*, 256.0568, Δ = 4.7 ppm.

#### *N*-(1-(2-(Ethylsulfonyl)ethyl)-2-methyl-1*H*-imidazol-5-yl)-4-fluorobenzenesulfonamide (**3f**)

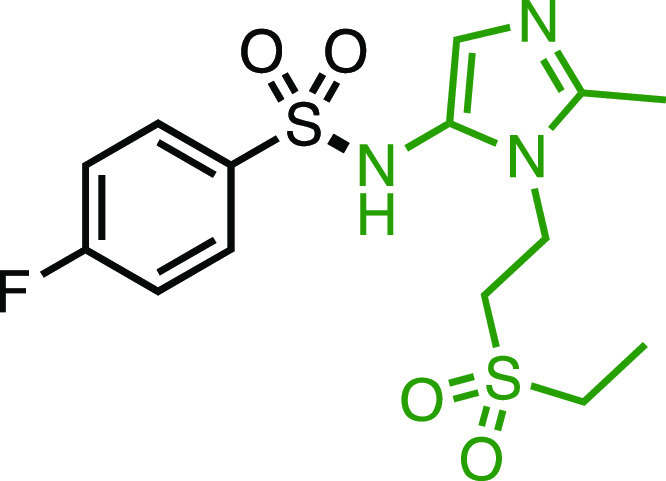



Following General Procedure 1, purification by flash column chromatography (0–2% MeOH in EtOAc) yielded **3f** as a brown solid (44 mg, 23%). ^1^H NMR (400 MHz, DMSO-*d*_6_): δ 7.94–7.79 (m, 2H), 7.46–7.32 (m, 2H), 5.92 (s, 1H), 4.19 (t, *J* = 7.2 Hz, 2H), 3.51 (t, *J* = 7.2 Hz, 2H), 3.10 (q, *J* = 7.4 Hz, 2H), 2.20 (s, 3H), 1.19 (t, *J* = 7.4 Hz, 3H), exchangeable proton not visible. ^13^C{^1^H} NMR (101 MHz, DMSO-*d*_6_): δ 164.1 (d, *J* = 250.5 Hz), 143.4, 139.89, 139.86, 129.1 (d, *J* = 9.5 Hz), 116.3 (d, *J* = 22.6 Hz), 111.9, 48.7, 46.9, 35.7, 12.9, 5.8. ^19^F{^1^H} NMR (376 MHz, DMSO-*d*_6_): δ −107.9. mp 119–121 °C. ν_max_: 1128 cm^–1^. HRMS: *m*/*z* calculated for C_14_H_19_N_3_O_4_FS_2_^+^, 376.0801 [M + H]^+^. Found *m*/*z*, 376.0810, Δ = 2.4 ppm.

#### 4-Fluoro-*N*-(5-methylpyridin-2-yl)benzenesulfonamide (**3g**)

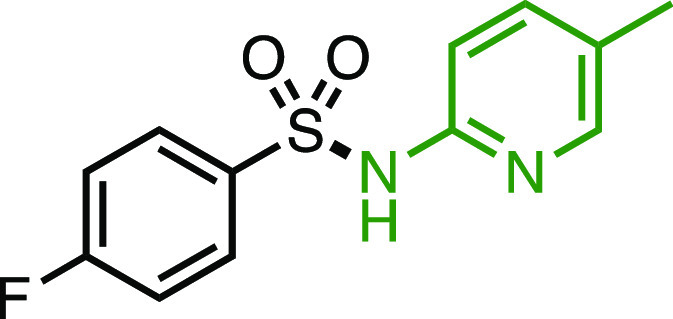



Following General Procedure 1, purification by flash column chromatography (40% EtOAc in petroleum ether) yielded **3g** as a white solid (60 mg, 45%). ^1^H NMR (400 MHz, DMSO-*d*_6_): δ 7.94–7.87 (m, 2H), 7.87–7.82 (m, 1H), 7.58 (dd, *J* = 8.8, 2.4 Hz, 1H), 7.40–7.31 (m, 2H), 7.09 (d, *J* = 8.8 Hz, 1H), 2.13 (s, 3H). ^13^C{^1^H} NMR (101 MHz, CDCl_3_): δ 164.7 (d, *J* = 253.2 Hz), 154.0, 144.7, 138.4 (d, *J* = 3.3 Hz), 138.3, 129.5 (d, *J* = 9.1 Hz), 124.0, 116.2 (d, *J* = 22.4 Hz), 115.2, 17.4. ^19^F{^1^H} NMR (376 MHz, DMSO-*d*_6_): δ −108.5. mp 196–198 °C. ν_max_: 1355, 1137 cm^–1^. HRMS: *m*/*z* calculated for C_12_H_12_N_2_O_2_FS^+^, 267.0604 [M + H]^+^. Found *m*/*z*, 267.0605, Δ = 0.4 ppm.

#### *tert*-Butyl 4-(5-((4-Fluorophenyl)sulfonamido)pyridin-2-yl)piperazine-1-carboxylate (**3h**)

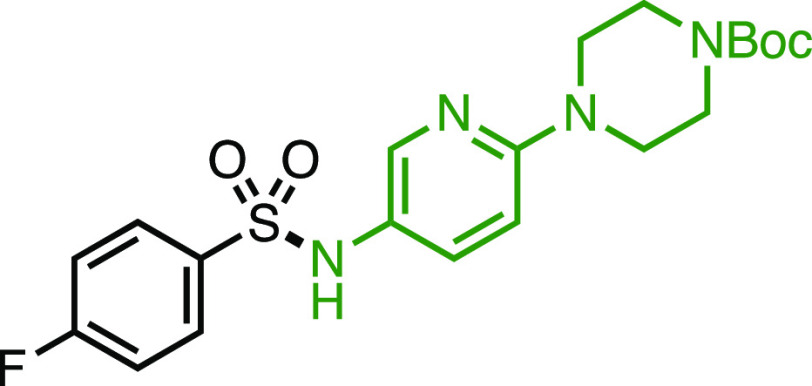



Following General Procedure 1, purification by flash column chromatography (30–40% EtOAc in petroleum ether) yielded **3h** as a yellow solid (81 mg, 37%).* ^1^H NMR (400 MHz, DMSO-*d*_6_): δ 9.84 (s, 1H), 7.77–7.66 (m, 3H), 7.46–7.34 (m, 2H), 7.20 (dd, *J* = 9.1, 2.8 Hz, 1H), 6.74 (d, *J* = 9.1 Hz, 1H), 3.41–3.34 (m, 8H), 1.40 (s, 9H). ^13^C{^1^H} NMR (101 MHz, CDCl_3_): δ 165.30 (d, *J* = 255.3 Hz), 157.9, 154.9, 144.8, 135.7, 135.08 (d, *J* = 3.3 Hz), 130.17 (d, *J* = 9.4 Hz), 122.8, 116.42 (d, *J* = 22.6 Hz), 107.1, 80.3, 45.1, 42.8, 28.5. ^19^F{^1^H} NMR (376 MHz, DMSO-*d*_6_): δ −107.2. mp 79–80 °C. ν_max_: 1660, 1364, 1153 cm^–1^. HRMS: *m*/*z* calculated for C_20_H_26_N_4_O_4_SF^+^, 437.1659 [M + H]^+^. Found *m*/*z*, 437.1653, Δ = −1.4 ppm.

*The reaction was repeated on a 1.0 mmol scale to produce **3h** as a white crystalline solid (237 mg, 54%).

#### 4-Fluoro-*N*-(4-(methylsulfinyl)phenyl)benzenesulfonamide (**3i**)

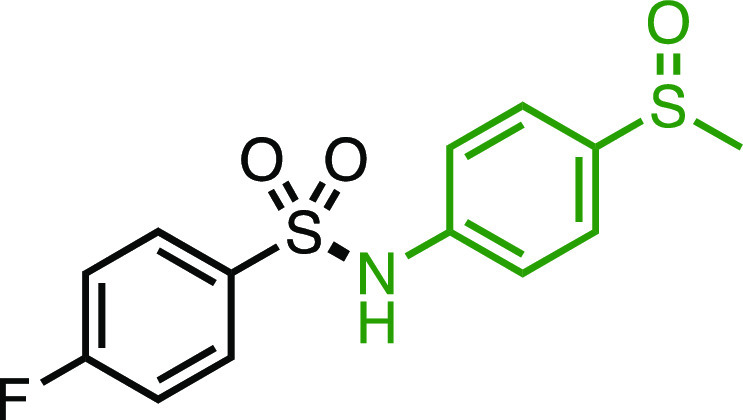



Following General Procedure 1, purification by flash column chromatography (0–4.5% MeOH in CH_2_Cl_2_) yielded **3i** as a pale yellow oil (64 mg, 41%). ^1^H NMR (400 MHz, DMSO-*d*_6_): δ 7.92–7.82 (m, 2H), 7.60–7.51 (m, 2H), 7.45–7.36 (m, 2H), 7.31–7.24 (m, 2H), 2.66 (s, 3H), exchangeable proton not visible. ^13^C{^1^H} NMR (101 MHz, DMSO-*d*_6_): δ 164.4 (d, *J* = 251.9 Hz), 140.9, 140.0, 135.7 (d, *J* = 3.0 Hz), 129.8 (d, *J* = 9.7 Hz), 125.1, 119.8, 116.7 (d, *J* = 22.9 Hz), 43.0. ^19^F{^1^H} NMR (376 MHz, DMSO-*d*_6_): δ −106.5. ν_max_: 1335, 1153, 1010 cm^–1^. HRMS: *m*/*z* calculated for C_13_H_13_NO_3_FS_2_^+^, 314.0321 [M + H]^+^. Found *m*/*z*, 314.0333, Δ = 3.8 ppm.

#### 4-Fluoro-*N*-(3-formylphenyl)benzenesulfonamide (**3j**)

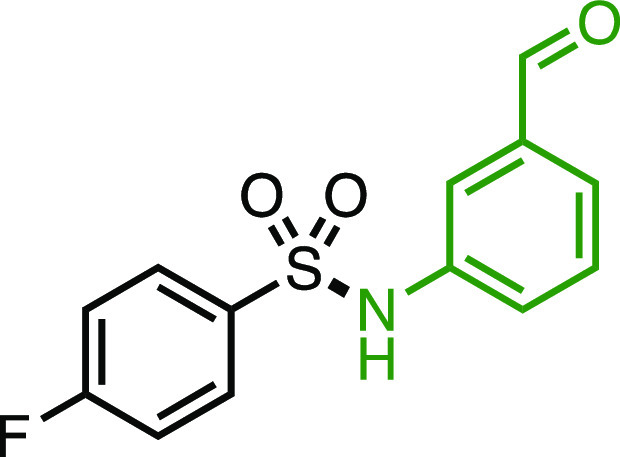



Following General Procedure 1, purification by flash column chromatography (0–30% EtOAc in petroleum ether) yielded **3j** as a colorless oil (48 mg, 34%). ^1^H NMR (400 MHz, DMSO-*d*_6_): δ 9.90 (s, 1H), 7.88–7.79 (m, 2H), 7.63–7.57 (m, 2H), 7.52–7.46 (m, 1H), 7.43–7.35 (m, 3H), exchangeable proton not visible. ^13^C{^1^H} NMR (101 MHz, DMSO-*d*_6_): δ 192.8, 164.5 (d, *J* = 251.9 Hz), 138.5, 137.1, 135.6 (d, *J* = 2.6 Hz), 130.3, 129.8 (d, *J* = 9.6 Hz), 126.2, 125.7, 119.3, 116.7 (d, *J* = 22.9 Hz). ^19^F{^1^H} NMR (376 MHz, DMSO-*d*_6_): δ −106.5. ν_max_: 1688, 1332, 1150 cm^–1^. HRMS: *m*/*z* calculated for C_13_H_11_NO_3_FS^+^, 280.0444 [M + H]^+^. Found *m*/*z*, 280.0445, Δ = 0.4 ppm. Data consistent with that available in the literature.^23^

#### 4-Fluoro-*N*-phenylbenzenesulfonamide (**3k**)

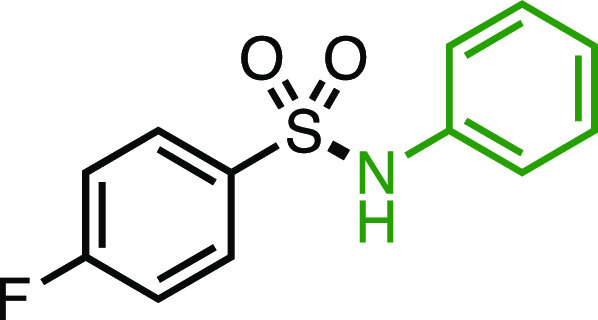



Following General Procedure 1, purification by flash column chromatography (0–50% EtOAc in petroleum ether) yielded **3k** as a colorless oil (79 mg, 63%). ^1^H NMR (400 MHz, DMSO-*d*_6_): δ 10.29 (s, 1H), 7.85–7.74 (m, 2H), 7.43–7.32 (m, 2H), 7.27–7.17 (m, 2H), 7.14–6.97 (m, 3H). ^13^C{^1^H} NMR (101 MHz, DMSO-*d*_6_): δ 164.3 (d, *J* = 251.7 Hz), 137.5, 135.8 (d, *J* = 3.0 Hz), 129.7 (d, *J* = 9.6 Hz), 129.2, 124.3, 120.3, 116.4 (d, *J* = 22.9 Hz). ^19^F{^1^H} NMR (376 MHz, DMSO-*d*_6_): δ −107.0. ν_max_: 1341, 1150 cm^–1^. HRMS: *m*/*z* calculated for C_12_H_11_NO_2_FS^+^, 252.0495 [M + H]^+^. Found *m*/*z*, 252.0489, Δ = −2.4 ppm. Data consistent with that in the literature.^7a^

#### *N*-(4-(2-Aminoethyl)phenyl)-4-fluorobenzenesulfonamide (**3l**)

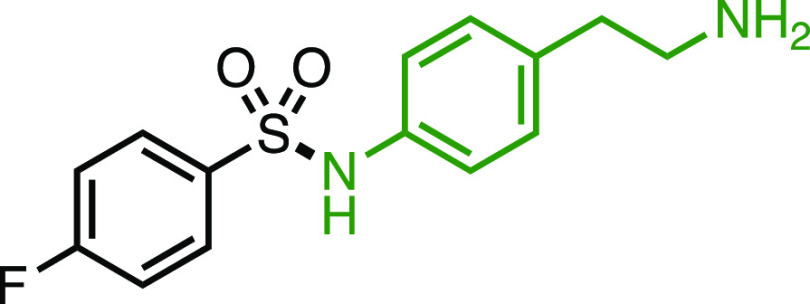



Following General Procedure 2, purification by flash column chromatography (0–10% MeOH, 1% NH_4_OH in CH_2_Cl_2_) yielded **3l** as a beige solid (95 mg, 64%). ^1^H NMR (400 MHz, DMSO-*d*_6_): δ 7.81–7.71 (m, 2H), 7.35–7.26 (m, 2H), 7.00–6.94 (m, 2H), 6.93–6.87 (m, 2H), 2.78–2.70 (m, 2H), 2.59–2.52 (m, 2H), exchangeable protons not visible. ^13^C{^1^H} NMR (101 MHz, DMSO-*d*_6_): δ 163.7 (d, *J* = 249.5 Hz), 139.3, 138.5 (d, *J* = 2.8 Hz), 133.0, 129.4 (d, *J* = 9.4 Hz), 129.1, 120.6, 115.9 (d, *J* = 22.5 Hz), 42.6, 37.1. ^19^F{^1^H} NMR (376 MHz, DMSO-*d*_6_): δ −109.1. mp 159–161 °C. ν_max_: 1292, 1110 cm^–1^. HRMS: *m*/*z* calculated for C_14_H_16_N_2_O_2_FS^+^, 295.0917 [M + H]^+^. Found *m*/*z*, 295.0927, Δ = 3.4 ppm.

#### *N*-(4-(Aminomethyl)phenyl)-4-fluorobenzenesulfonamide (**3m**)

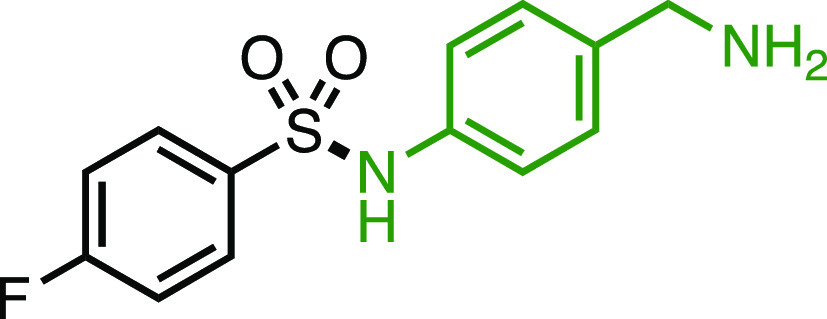



Following General Procedure 2, purification by flash column chromatography (0–10% MeOH, 1% NH_4_OH in CH_2_Cl_2_) yielded **3m** as a beige solid (93 mg, 67%). ^1^H NMR (400 MHz, DMSO-*d*_6_): δ 7.81–7.72 (m, 2H), 7.35–7.27 (m, 2H), 7.15–7.09 (m, 2H), 6.98–6.90 (m, 2H), 3.65 (s, 2H), exchangeable protons not visible. ^13^C{^1^H} NMR (101 MHz, DMSO-*d*_6_): δ 163.69 (d, *J* = 249.7 Hz), 139.6, 138.10 (d, *J* = 3.1 Hz), 135.0, 129.4 (d, *J* = 9.4 Hz), 128.1, 120.4, 115.9 (d, *J* = 22.3 Hz), 44.2. ^19^F{^1^H} NMR (376 MHz, DMSO-*d*_6_): δ −108.9. mp 151–153 °C. ν_max_: 1335, 1153, 1010 cm^–1^. HRMS: *m*/*z* calculated for C_13_H_12_N_2_O_2_SF^+^, 279.0604 [M + H]^+^. Found *m*/*z*, 279.0611, Δ = 2.5 ppm.

#### *N*-(1-Methyl-1*H*-pyrazol-3-yl)-5-(5-(trifluoromethyl)isoxazol-3-yl)thiophene-2-sulfonamide (**3n**)

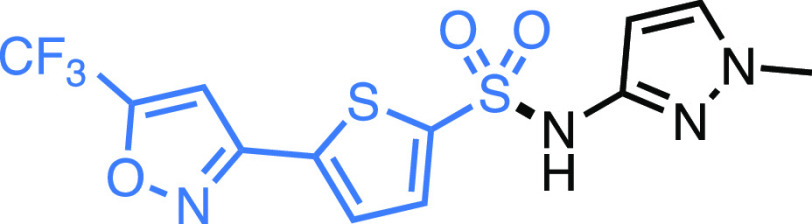



Following General Procedure 1, purification by flash column chromatography (0–50% EtOAc in in petroleum ether) yielded **3n** as a pale yellow solid (45 mg, 23%). ^1^H NMR (400 MHz, DMSO-*d*_6_): δ 8.13–8.10 (m, 1H), 7.83 (d, *J* = 4.0 Hz, 1H), 7.67 (d, *J* = 4.0 Hz, 1H), 7.56 (d, *J* = 2.3 Hz, 1H), 6.01 (d, *J* = 2.3 Hz, 1H), 3.68 (s, 3H), exchangeable proton not visible. ^13^C{^1^H} NMR (101 MHz, DMSO-*d*_6_): δ 157.8 (q, *J* = 42.4 Hz), 157.5, 144.5, 143.5, 132.9, 132.6, 132.0, 130.6, 117.6 (q, *J* = 270.3 Hz), 105.9 (q, *J* = 1.9 Hz), 97.8, 38.6. ^19^F{^1^H} NMR (376 MHz, CDCl_3_): δ −65.2. mp 130–132 °C. ν_max_: 1364, 1157 cm^–1^. HRMS: *m*/*z* calculated for C_12_H_10_N_4_O_3_S_2_F_3_^+^, 379.0146 [M + H]^+^. Found *m*/*z*, 379.0157, Δ = 2.9 ppm.

#### *N*-(1-Methyl-1*H*-pyrazol-3-yl)-5-(2-(methylthio)pyrimidin-4-yl)thiophene-2-sulfonamide (**3o**)

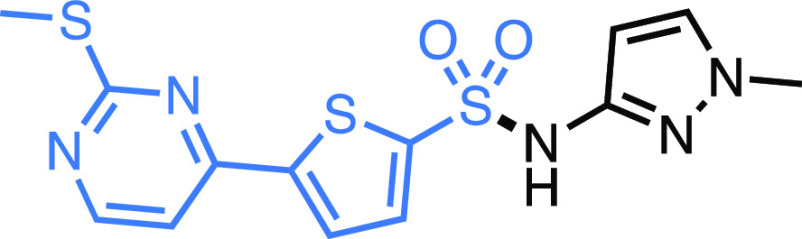



Following General Procedure 1, purification by flash column chromatography (0–55% EtOAc in petroleum ether) yielded **3o** as a yellow solid (36 mg, 19%). ^1^H NMR (400 MHz, DMSO-*d*_6_): δ 10.83 (s, 1H), 8.69 (d, *J* = 5.2 Hz, 1H), 8.02 (d, *J* = 4.1 Hz, 1H), 7.75 (d, *J* = 5.2 Hz, 1H), 7.61 (d, *J* = 4.1 Hz, 1H), 7.55 (d, *J* = 2.3 Hz, 1H), 6.00 (d, *J* = 2.3 Hz, 1H), 3.67 (s, 3H), 2.54 (s, 3H). ^13^C{^1^H} NMR (101 MHz, DMSO-*d*_6_): δ 171.8, 158.8, 157.2, 146.6, 144.6, 144.1, 132.9, 131.9, 128.6, 111.2, 97.6, 38.5, 13.5. mp 166–169 °C. ν_max_: 1350, 1163 cm^–1^. HRMS: *m*/*z* calculated for C_13_H_14_N_5_O_2_S_3_^+^, 368.0310 [M + H]^+^. Found *m*/*z*, 368.0326, Δ = 4.3 ppm.

#### 3,5-Dimethyl-*N*-(1-methyl-1*H*-pyrazol-3-yl)isoxazole-4-sulfonamide (**3p**)

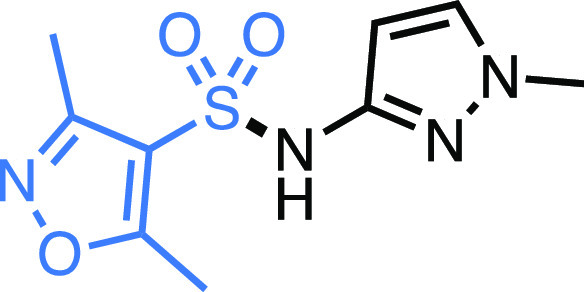



Following General Procedure 1, purification by flash column chromatography (50–100% EtOAc in petroleum ether) yielded **3p** as a white solid (79 mg, 61%). ^1^H NMR (400 MHz, CDCl_3_): δ 7.24 (d, *J* = 2.4 Hz, 1H), 6.22 (d, *J* = 2.4 Hz, 1H), 3.88 (s, 3H), 2.49 (s, 3H), 2.18 (s, 3H), exchangeable proton not visible. ^13^C{^1^H} NMR (101 MHz, CDCl_3_): δ 173.8, 157.7, 145.4, 132.2, 115.8, 98.1, 39.2, 12.8, 10.7. mp 117–119 °C. ν_max_: 1355, 1125 cm^–1^. HRMS: *m*/*z* calculated for C_9_H_13_N_4_O_3_S^+^, 257.0708 [M + H]^+^. Found *m*/*z*, 257.0707, Δ = −0.4 ppm.

#### *N*-(1-Methyl-1*H*-pyrazol-3-yl)-4-((5-(trifluoromethyl)pyridin-2-yl)oxy)benzenesulfonamide (**3q**)

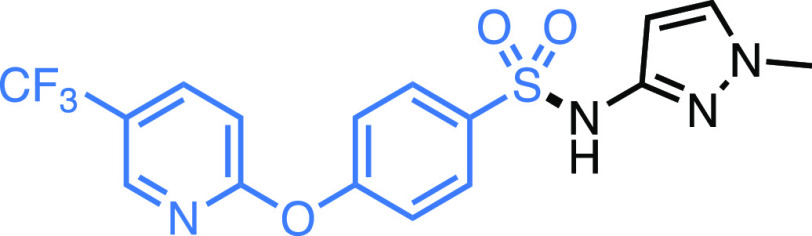



Following General Procedure 1, purification by flash column chromatography (100% CHCl_3_ → 0–50% EtOAc in petroleum ether) yielded **3q** as a pale yellow solid (74 mg, 37%). ^1^H NMR (400 MHz, DMSO-*d*_6_): δ 10.53 (s, 1H), 8.64–8.56 (m, 1H), 8.28 (dd, *J* = 8.7, 2.6 Hz, 1H), 7.88–7.81 (m, 2H), 7.51 (d, *J* = 2.3 Hz, 1H), 7.43–7.37 (m, 2H), 7.37–7.31 (m, 1H), 5.97 (d, *J* = 2.3 Hz, 1H), 3.66 (s, 3H). ^13^C{^1^H} NMR (101 MHz, DMSO-*d*_6_): δ 164.7, 156.2, 145.3 (q, *J* = 4.5 Hz), 145.2, 138.0 (q, *J* = 3.3 Hz), 136.9, 131.8, 128.8, 123.8 (q, *J* = 271.5 Hz), 122.0, 121.1 (q, *J* = 32.8 Hz), 112.5, 97.1, 38.5. ^19^F{^1^H} NMR (376 MHz, DMSO-*d*_6_): δ −61.1. mp 53–54 °C. ν_max_: 1357, 1155 cm^–1^. HRMS: *m*/*z* calculated for C_16_H_14_N_4_O_3_F_3_S^+^, 399.0739 [M + H]^+^. Found *m*/*z*, 399.0756, Δ = 4.3 ppm.

#### *N*-(1-Methyl-1*H*-pyrazol-3-yl)-4-(5-methyl-3-phenylisoxazol-4-yl)benzenesulfonamide (**3r**)

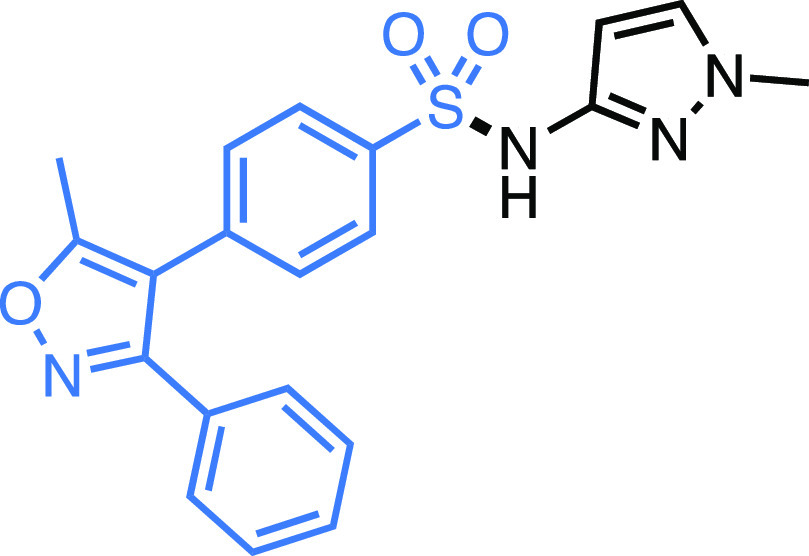



Following General Procedure 1, purification by flash column chromatography (0–70% EtOAc in petroleum ether) yielded **3r** as a white solid (104 mg, 53%). ^1^H NMR (400 MHz, DMSO-*d*_6_): δ 10.47 (s, 1H), 7.80–7.75 (m, 2H), 7.51 (d, *J* = 2.3 Hz, 1H), 7.49–7.35 (m, 5H), 7.33–7.27 (m, 2H), 5.90 (d, *J* = 2.3 Hz, 1H), 3.65 (s, 3H), 2.45 (s, 3H). ^13^C{^1^H} NMR (101 MHz, DMSO-*d*_6_): δ 167.7, 160.7, 145.0, 139.4, 134.2, 131.8, 130.1, 129.9, 128.8, 128.3, 128.2, 127.2, 114.2, 97.6, 38.5, 11.4. mp 185–187 °C. ν_max_: 1356, 1161 cm^–1^. HRMS: *m*/*z* calculated for C_20_H_19_N_4_O_3_S^+^, 395.1178 [M + H]^+^. Found *m*/*z*, 395.1196, Δ = 4.6 ppm.

#### 4-Methoxy-*N*-(1-methyl-1*H*-pyrazol-3-yl)benzenesulfonamide (**3s**)

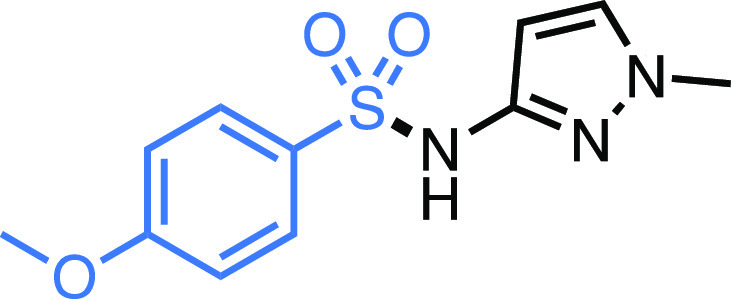



Following General Procedure 1, purification by flash column chromatography (30–55% EtOAc in petroleum ether) yielded **3s** as a white solid (82 mg, 62%). ^1^H NMR (400 MHz, DMSO-*d*_6_): δ 10.30 (s, 1H), 7.76–7.63 (m, 2H), 7.46 (d, *J* = 2.3 Hz, 1H), 7.13–6.98 (m, 2H), 5.89 (d, *J* = 2.3 Hz, 1H), 3.80 (s, 3H), 3.62 (s, 3H). ^13^C{^1^H} NMR (101 MHz, DMSO-*d*_6_): δ 162.3, 145.5, 131.9, 131.7, 128.9, 114.2, 97.0, 55.7, 38.4. mp 118–120 °C. ν_max_: 1352, 1152 cm^–1^. HRMS: *m*/*z* calculated for C_11_H_14_N_3_O_3_S^+^, 268.0756 [M + H]^+^. Found *m*/*z*, 268.0766, Δ = 3.7 ppm.

#### *N*-(1-Methyl-1*H*-pyrazol-3-yl)-4-phenoxybenzenesulfonamide (**3t**)

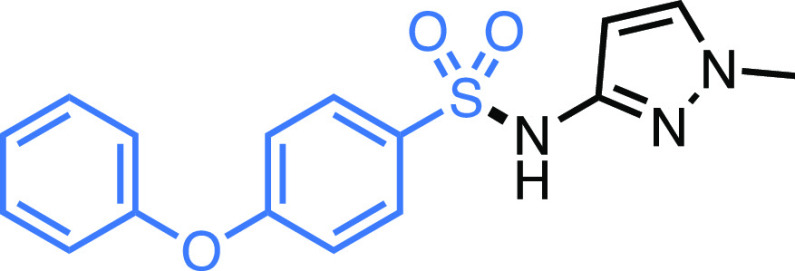



Following General Procedure 1, purification by flash column chromatography (0–40% EtOAc in petroleum ether) yielded **3t** as a white solid (90 mg, 55%). ^1^H NMR (400 MHz, CDCl_3_): δ 7.66–7.59 (m, 2H), 7.42–7.32 (m, 2H), 7.22–7.15 (m, 2H), 7.07–6.98 (m, 2H), 6.94–6.86 (m, 2H), 6.24 (d, *J* = 2.4 Hz, 1H), 3.85 (s, 3H). ^13^C{^1^H} NMR (101 MHz, CDCl_3_): δ 161.7, 155.3, 146.5, 133.3, 131.9, 130.3, 129.3, 125.0, 120.4, 117.6, 97.7, 39.1. mp 137–139 °C. ν_max_: 1357, 1153 cm^–1^. HRMS: *m*/*z* calculated for C_16_H_16_N_3_O_3_S^+^, 330.0912 [M + H]^+^. Found *m*/*z*, 330.0920, Δ = 2.4 ppm.

#### *N*-(1-Methyl-1*H*-pyrazol-3-yl)-4-(methylsulfonyl)benzenesulfonamide (**3u**)

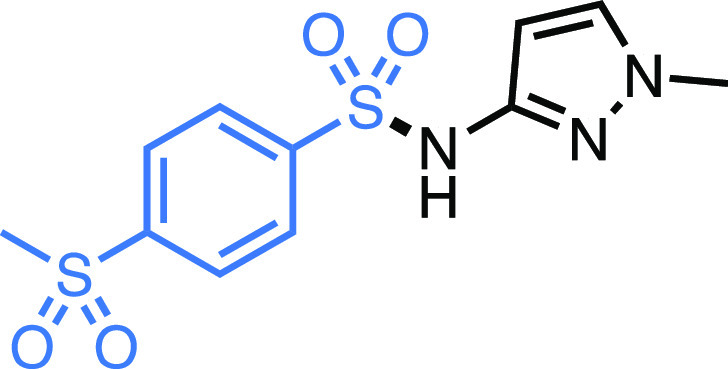



Following General Procedure 1, purification by flash column chromatography (0–70% EtOAc in petroleum ether) yielded **3u** as a white solid (89 mg, 56%). ^1^H NMR (400 MHz, DMSO-*d*_6_): δ 10.76 (s, 1H), 8.17–8.07 (m, 2H), 8.07–7.96 (m, 2H), 7.52 (d, *J* = 2.3 Hz, 1H), 5.95 (d, *J* = 2.3 Hz, 1H), 3.64 (s, 3H), 3.29 (s, 3H). ^13^C{^1^H} NMR (101 MHz, DMSO-*d*_6_): δ 144.7, 144.5, 144.2, 131.9, 128.0, 127.7, 97.5, 43.1, 38.5. mp 213–215 °C. ν_max_: 1308, 1166 cm^–1^. HRMS: *m*/*z* calculated for C_11_H_14_N_3_O_4_S_2_^+^, 316.0426 [M + H]^+^. Found *m*/*z*, 316.0416, Δ = −3.2 ppm.

#### 4-Methyl-*N*-(1-methyl-1*H*-pyrazol-3-yl)benzenesulfonamide (**3v**)

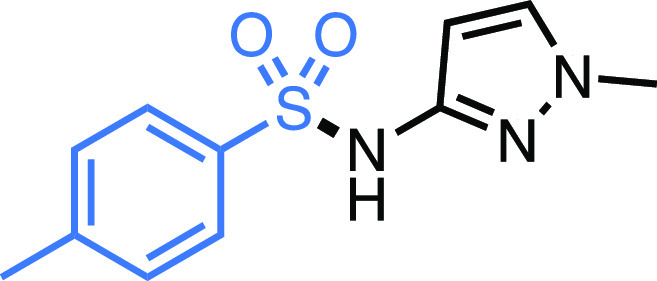



Following General Procedure 1, purification by flash column chromatography (100% CHCl_3_ → 0–50% EtOAc in petroleum ether) yielded **3v** as a white solid (71 mg, 57%). ^1^H NMR (400 MHz, DMSO-*d*_6_): δ 10.38 (s, 1H), 7.69–7.59 (m, 2H), 7.46 (d, *J* = 2.3 Hz, 1H), 7.38–7.27 (m, 2H), 5.89 (d, *J* = 2.3 Hz, 1H), 3.62 (s, 3H), 2.34 (s, 3H). ^13^C{^1^H} NMR (101 MHz, DMSO-*d*_6_): δ 145.3, 142.9, 137.3, 131.6, 129.5, 126.7, 97.0, 38.4, 21.0. mp 122–124 °C. ν_max_: 1354, 1160 cm^–1^. HRMS: *m*/*z* calculated for C_11_H_14_N_3_O_2_S^+^, 252.0807 [M + H]^+^. Found *m*/*z*, 252.0814, Δ = 2.5 ppm.

#### Methyl 4-(*N*-(1-Methyl-1*H*-pyrazol-3-yl)sulfamoyl)benzoate (**3w**)

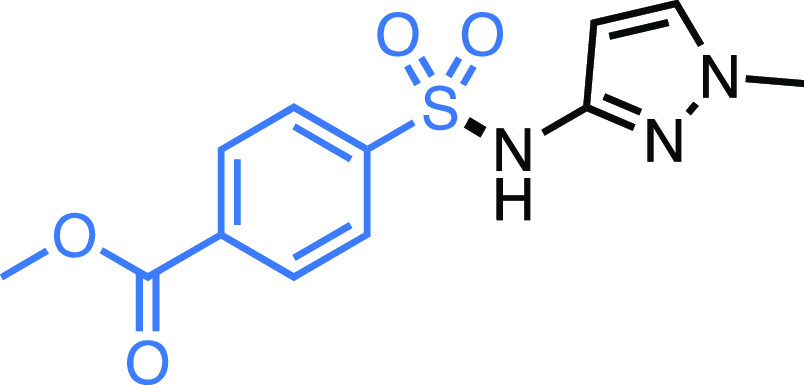



Following General Procedure 1, purification by flash column chromatography (50–70% EtOAc in petroleum ether) yielded **3w** as a white solid (64 mg, 44%). ^1^H NMR (400 MHz, DMSO-*d*_6_): δ 10.64 (s, 1H), 8.15–8.05 (m, 2H), 7.94–7.84 (m, 2H), 7.49 (d, *J* = 2.3 Hz, 1H), 5.92 (d, *J* = 2.3 Hz, 1H), 3.87 (s, 3H), 3.63 (s, 3H). ^13^C{^1^H} NMR (101 MHz, DMSO-*d*_6_): δ 165.2, 144.7, 144.1, 133.1, 131.8, 129.9, 127.2, 97.5, 52.6, 38.5. mp 188–190 °C. ν_max_: 1722, 1355, 1163 cm^–1^. HRMS: *m*/*z* calculated for C_12_H_14_N_3_O_4_S^+^, 296.0705 [M + H]^+^. Found *m*/*z*, 296.0710, Δ = 1.7 ppm.

#### *N*-(1-Methyl-1*H*-pyrazol-3-yl)-3,5-bis(trifluoromethyl)benzenesulfonamide (**3x**)

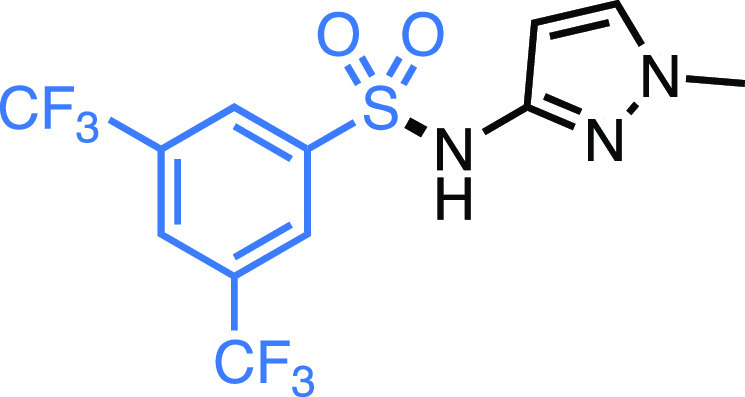



Following General Procedure 1, purification by flash column chromatography (20–80% EtOAc in petroleum ether) yielded **3x** as a pale yellow solid (87 mg, 47%). ^1^H NMR (400 MHz, DMSO-*d*_6_): δ 10.76 (s, 1H), 8.55–8.45 (m, 1H), 8.37–8.25 (m, 2H), 7.56 (d, *J* = 2.3 Hz, 1H), 5.99 (d, *J* = 2.3 Hz, 1H), 3.64 (s, 3H). ^13^C{^1^H} NMR (101 MHz, DMSO-*d*_6_): δ 144.1, 142.6, 132.2, 131.1 (q, *J* = 33.9 Hz), 127.6–127.3 (m), 126.9–126.8 (m), 122.6 (q, *J* = 273.8 Hz), 98.5, 38.5. ^19^F{^1^H} NMR (376 MHz, DMSO-*d*_6_): δ −62.6. mp 156–158 °C. ν_max_: 1360, 1162 cm^–1^. HRMS: *m*/*z* calculated for C_12_H_10_N_3_O_2_F_6_S ^+^, 374.0398 [M + H]^+^. Found *m*/*z*, 374.0411, Δ = 3.5 ppm.

#### Ethyl 5-((4-((5-(Trifluoromethyl)pyridin-2-yl)oxy)phenyl)sulfonamido)-1*H*-pyrazole-3-carboxylate (**3y**)

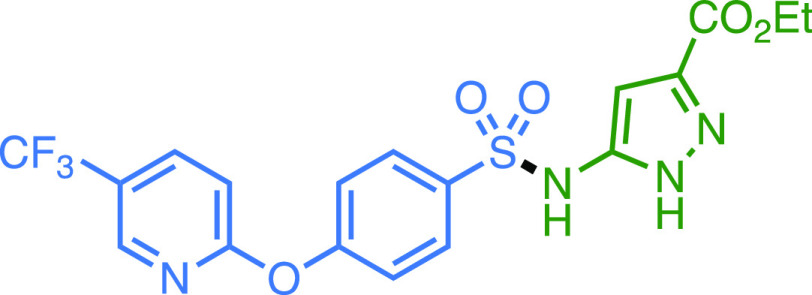



Following General Procedure 1, purification by flash column chromatography (50–70% EtOAc in petroleum ether) yielded **3y** as a white solid (86 mg, 38%). ^1^H NMR (400 MHz, DMSO-*d*_6_): δ 13.64 (s, 1H), 10.81 (s, 1H), 8.71–8.47 (m, 1H), 8.28 (dd, *J* = 8.7, 2.7 Hz, 1H), 7.90–7.79 (m, 2H), 7.46–7.28 (m, 3H), 6.50 (s, 1H), 4.28 (q, *J* = 7.1 Hz, 2H), 1.28 (t, *J* = 7.1 Hz, 3H). ^13^C{^1^H} NMR (101 MHz, DMSO-*d*_6_): δ 164.7, 156.4, 145.4 (q, *J* = 4.2 Hz), 138.1 (q, *J* = 3.1 Hz), 136.6, 133.9, 129.0, 128.8, 123.8 (q, *J* = 271.7 Hz), 122.2, 121.2 (q, *J* = 32.7 Hz), 112.6, 104.8, 100.2, 61.0, 14.1. ^19^F{^1^H} NMR (376 MHz, DMSO-*d*_6_): δ −60.7. mp 151–153 °C. ν_max_: 1702, 1323, 1159 cm^–1^. HRMS: *m*/*z* calculated for C_18_H_16_N_4_O_5_SF_3_^+^, 457.0793 [M + H]^+^. Found *m*/*z*, 457.0797, Δ = 0.9 ppm.

#### 4-(5-Methyl-3-phenylisoxazol-4-yl)-*N*-(5-methylpyridin-2-yl)benzenesulfonamide (**3z**)

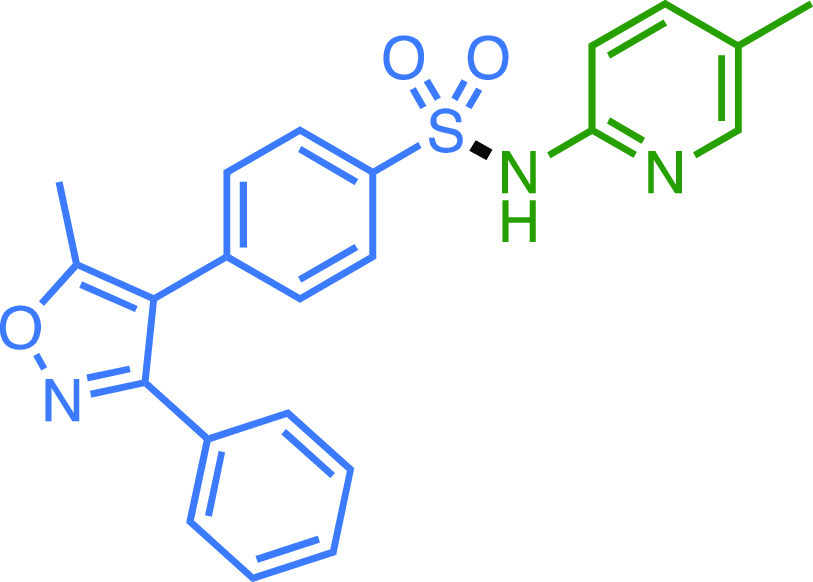



Following General Procedure 1, purification by flash column chromatography (0–60% EtOAc in petroleum ether) yielded **3z** as a white solid (65 mg, 32%). ^1^H NMR (400 MHz, DMSO-*d*_6_): δ 7.89–7.80 (m, 3H), 7.59 (dd, *J* = 8.8, 2.4 Hz, 1H), 7.49–7.32 (m, 5H), 7.32–7.25 (m, 2H), 7.13 (d, *J* = 8.8 Hz, 1H), 2.44 (s, 3H), 2.15 (s, 3H), exchangeable proton not visible. ^13^C{^1^H} NMR (101 MHz, CDCl_3_): δ 167.3, 161.2, 154.0, 144.6, 141.5, 138.2, 134.5, 130.2, 129.8, 128.8, 128.7, 128.6, 127.2, 124.0, 115.4, 114.7, 17.4, 11.9. mp 217–219 °C. ν_max_: 1361, 1141 cm^–1^. HRMS: *m*/*z* calculated for C_22_H_20_N_3_O_3_S^+^, 406.1225 [M + H]^+^. Found *m*/*z*, 406.1231, Δ = 1.5 ppm.

#### Methyl 4-(*N*-(1*H*-Pyrrolo[2,3-*b*]pyridin-5-yl)sulfamoyl)benzoate (**3aa**)

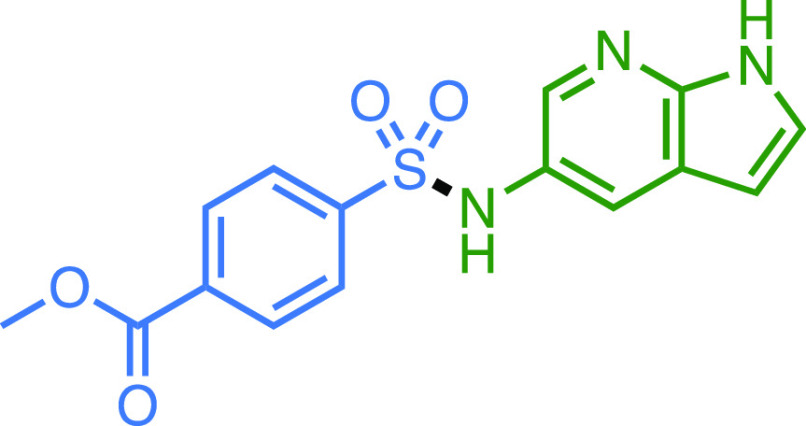



Following General Procedure 1, purification by flash column chromatography (50–70% EtOAc in petroleum ether) yielded **3aa** as an orange solid (102 mg, 61%). ^1^H NMR (400 MHz, DMSO-*d*_6_): δ 11.66 (s, 1H), 10.20 (s, 1H), 8.09–8.03 (m, 2H), 7.83–7.81 (m, 1H), 7.80–7.76 (m, 2H), 7.63–7.58 (m, 1H), 7.47–7.41 (m, 1H), 6.39 (dd, *J* = 3.4, 1.8 Hz, 1H), 3.85 (s, 3H). ^13^C{^1^H} NMR (101 MHz, DMSO-*d*_6_): δ 165.1, 146.5, 143.2, 138.9, 133.1, 130.0, 127.5, 127.2, 125.9, 123.4, 119.4, 100.0, 52.6. mp 188–190 °C. ν_max_: 1725, 1333, 1160 cm^–1^. HRMS: *m*/*z* calculated for C_15_H_14_N_3_O_4_S^+^, 332.0705 [M + H]^+^. Found *m*/*z*, 332.0710, Δ = 1.5 ppm.

#### Ethyl 5-((4-Methoxyphenyl)sulfonamido)-1*H*-pyrazole-3-carboxylate (**3ab**)

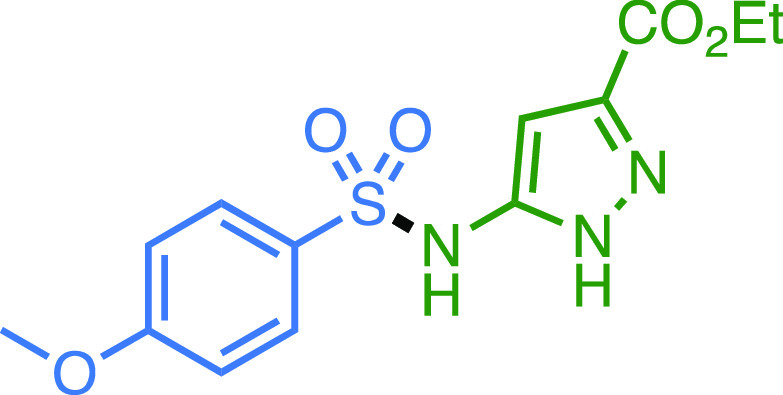



Following General Procedure 1, purification by flash column chromatography (40–80% EtOAc in petroleum ether) yielded **3ab** as a white solid (104 mg, 65%). ^1^H NMR (400 MHz, DMSO-*d*_6_): δ 13.55 (s, 1H), 10.58 (s, 1H), 7.79–7.59 (m, 2H), 7.21–6.98 (m, 2H), 6.43 (s, 1H), 4.26 (q, *J* = 7.0 Hz, 2H), 3.81 (s, 3H), 1.27 (t, *J* = 7.0 Hz, 3H). ^13^C{^1^H} NMR (101 MHz, DMSO-*d*_6_): δ 162.5, 158.6, 146.7, 133.7, 131.6, 128.9, 114.4, 100.0, 61.0, 55.7, 14.1. mp 129–131 °C. ν_max_: 1722, 1341, 1159 cm^–1^. HRMS: *m*/*z* calculated for C_13_H_16_N_3_O_5_S^+^, 326.0811 [M + H]^+^. Found *m*/*z*, 326.0824, Δ = 4.0 ppm.

#### Methyl 4-(*N*-(1*H*-Pyrazol-3-yl)sulfamoyl)benzoate (**3ac**)

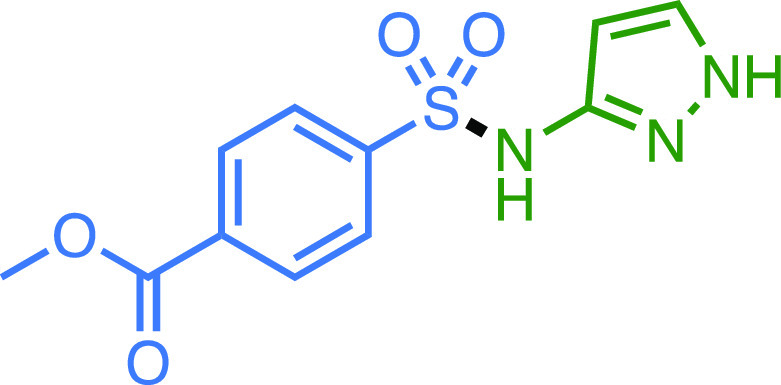



Following General Procedure 1, purification by flash column chromatography (50–80% EtOAc in petroleum ether) yielded **3ac** as a white solid (53 mg, 37%). ^1^H NMR (400 MHz, DMSO-*d*_6_): δ 12.39 (s, 1H), 10.59 (s, 1H), 8.16–8.04 (m, 2H), 7.96–7.84 (m, 2H), 7.61–7.49 (m, 1H), 6.02–5.88 (m, 1H), 3.87 (s, 3H). ^13^C{^1^H} NMR (101 MHz, DMSO-*d*_6_): δ 165.2, 145.2, 144.2, 133.1, 129.8, 129.6, 127.2, 97.4, 52.6. mp 214–216 °C. ν_max_: 1725, 1333, 1160 cm^–1^. HRMS: *m*/*z* calculated for C_15_H_14_N_3_O_4_S^+^, 332.0705 [M + H]^+^. Found *m*/*z*, 332.0710, Δ = 1.5 ppm.

#### 3,5-Dimethyl-*N*-(1*H*-pyrazol-3-yl)isoxazole-4-sulfonamide (**3ad**)

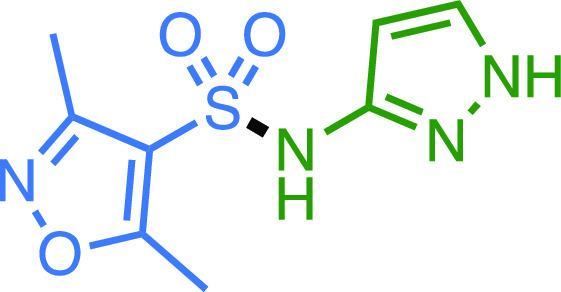



Following General Procedure 1, purification by flash column chromatography (50–100% EtOAc in petroleum ether) yielded **3ad** as a white solid (56 mg, 46%). ^1^H NMR (400 MHz, DMSO-*d*_6_): δ 12.52 (s, 1H), 10.49 (s, 1H), 7.61 (d, *J* = 2.4 Hz, 1H), 5.98 (d, *J* = 2.4 Hz, 1H), 2.44 (s, 3H), 2.22 (s, 3H). ^13^C{^1^H} NMR (101 MHz, DMSO-*d*_6_): δ 173.0, 157.5, 144.7, 129.8, 116.0, 98.3, 12.2, 10.3. mp 165–167 °C. ν_max_: 1348, 1178 cm^–1^. HRMS: *m*/*z* calculated for C_8_H_11_N_4_O_3_S^+^, 243.0552 [M + H]^+^. Found *m*/*z*, 243.0546, Δ = −2.5 ppm.

#### *N*-(1*H*-Pyrazol-3-yl)-3,5-bis(trifluoromethyl)benzenesulfonamide (**3ae**)

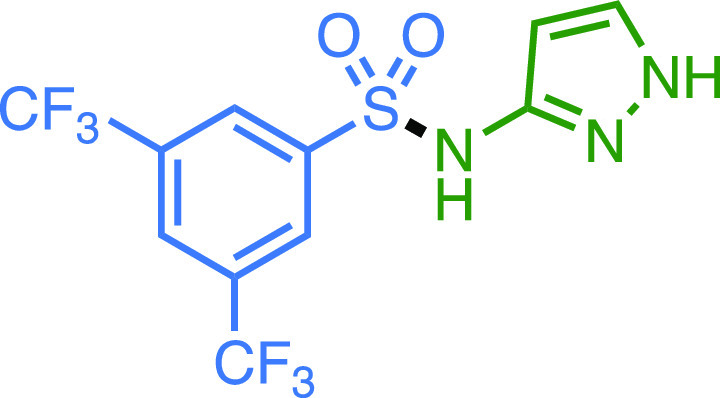



Following General Procedure 1, purification by flash column chromatography (30–40% EtOAc in petroleum ether) yielded **3ae** (29 mg, 16%) as a white solid. ^1^H NMR (400 MHz, DMSO-*d*_6_): δ 12.53 (s, 1H), 10.75 (s, 1H), 8.47 (s, 1H), 8.31 (s, 2H), 7.61 (d, *J* = 2.4 Hz, 1H), 6.03 (d, *J* = 2.4 Hz, 1H). ^13^C{^1^H} NMR (101 MHz, DMSO-*d*_6_): δ 144.7, 142.9, 131.2 (q, *J* = 33.9 Hz), 130.0, 127.5–127.3 (m), 127.0–126.7 (m), 122.6 (q, *J* = 273.5 Hz), 98.2. ^19^F{^1^H} NMR (376 MHz, DMSO-*d*_6_): δ −62.5. mp 216–218 °C. ν_max_: 1358, 1156 cm^–1^. HRMS: *m*/*z* calculated for C_11_H_8_N_3_O_2_SF_6_^+^, 360.0241 [M + H]^+^. Found *m*/*z*, 360.0236, Δ = −1.4 ppm.

#### Ethyl 5-((4-(Methylsulfonyl)phenyl)sulfonamido)-1*H*-pyrazole-3-carboxylate (**3ag**)

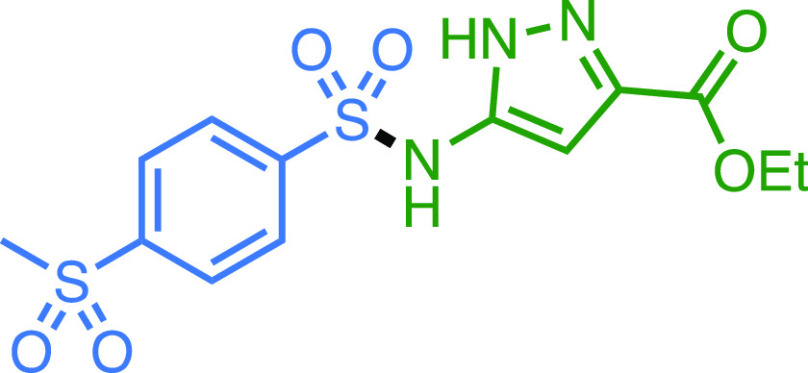



Following General Procedure 3 on a 0.2 mmol scale, flash column chromatography (5% MeOH in DCM) afforded **3ag** as a white crystalline solid (24.5 mg, 66%). ^1^H NMR (400 MHz, DMSO-*d*_6_): δ 13.67 (s, 1H), 11.04 (s, 1H), 8.19–8.08 (m, 2H), 8.02 (app d, *J* = 8.2 Hz, 2H), 6.50 (s, 1H), 4.27 (q, *J* = 7.1 Hz, 2H), 3.29 (s, 3H), 1.28 (t, *J* = 7.1 Hz, 3H). ^13^C{^1^H} NMR (101 MHz, DMSO-*d*_6_): δ 158.4, 158.0, 145.8, 144.4, 133.9, 128.1, 127.7, 100.5, 61.0, 43.0, 14.1. mp 138–140 °C. IR: ν_max_, 3118, 2921, 2851, 1708, 1562, 1536, 1235 cm^–1^. HRMS: *m*/*z* calculated for C_13_H_16_N_3_O_6_S_2_^+^, 374.0475 [M + H]^+^. Found *m*/*z*, 374.0457, Δ = −4.8 ppm. *R*_f_ (5% MeOH in DCM) = 0.28.

#### *N*-(6-Chloroimidazo[1,2-*b*]pyridazin-3-yl)-4-fluorobenzenesulfonamide (**3ah**)

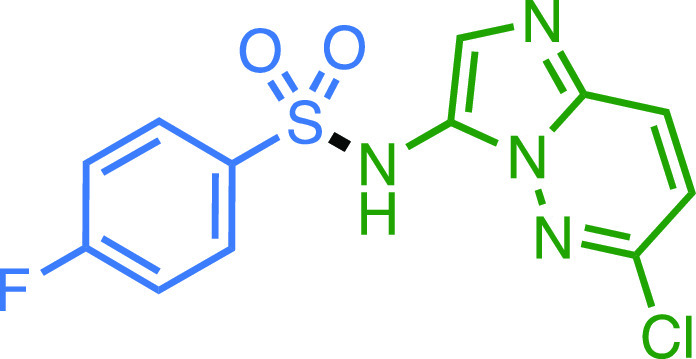



Following General Procedure 3 on a 0.5 mmol scale, flash column chromatography (0–5% MeOH in DCM) afforded **3ah** as a white crystalline solid (102 mg, 62%). ^1^H NMR (500 MHz, MeOD-*d*_4_): δ 7.93 (d, *J* = 9.5 Hz, 1H), 7.80 (ddd, *J* = 10.1, 5.1, 2.6 Hz, 2H), 7.62 (s, 1H), 7.23–7.17 (m, 3H). ^13^C{^1^H} NMR (126 MHz, MeOD-*d*_4_): δ 166.8 (d, *J* = 253.3 Hz), 148.9, 138.1 (d, *J* = 3.2 Hz), 137.2, 131.3 (d, *J* = 9.6 Hz), 131.1, 128.4, 124.6, 121.1, 117.1 (d, *J* = 23.1 Hz). ^19^F{^1^H} NMR (376 MHz, MeOD-*d*_4_): δ −107.5. mp 200–206 °C. IR: ν_max_, 3097, 1175, 1126, 1080 cm^–1^. HRMS: *m*/*z* calculated for C_12_H_9_ClFN_4_O_2_S^+^, 327.0113 [M + H]^+^. Found *m*/*z*, 327.0104, Δ = −3.0 ppm. *R*_f_ (5% MeOH in DCM) = 0.19.

#### *N*-(6-Chloropyridin-3-yl)-4-fluorobenzenesulfonamide (**3ai**)

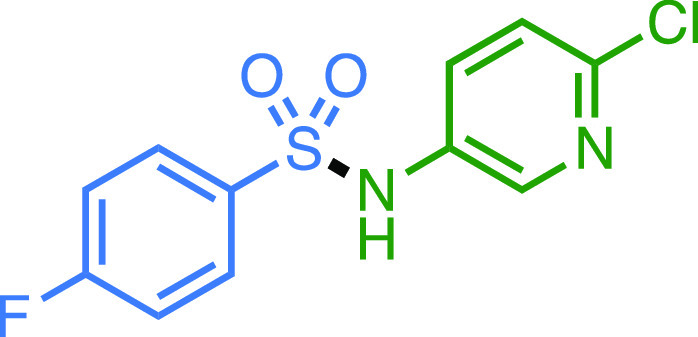



Following General Procedure 3 on a 0.2 mmol scale, flash column chromatography (20% EtOAc in petroleum ether) afforded **3ai** as a white crystalline solid (39.0 mg, 68%). ^1^H NMR (400 MHz, CDCl_3_): δ 8.09 (d, *J* = 2.6 Hz, 1H), 7.79 (ddd, *J* = 9.9, 5.0, 2.6 Hz, 2H), 7.73 (s, 1H), 7.60 (dd, *J* = 8.6, 2.6 Hz, 1H), 7.26 (d, *J* = 8.6 Hz, 1H), 7.19–7.11 (m, 2H). ^13^C{^1^H} NMR (101 MHz, CDCl_3_): δ 165.7 (d, *J* = 256.9 Hz), 148.1, 142.9, 134.5 (d, *J* = 3.3 Hz), 132.6, 132.6, 130.1 (d, *J* = 9.5 Hz), 125.0, 116.9 (d, *J* = 22.8 Hz). mp 137–139 °C. IR: ν_max_, 3109, 2890, 1589, 1494, 1458, 1325, 1171, 1155, 1088 cm^–1^. HRMS: *m*/*z* calculated for C_11_H_9_ClFN_2_O_2_S^+^, 287.0052 [M + H]^+^. Found *m*/*z*, 287.0052, Δ = 0.1 ppm.

#### *N*-(4-(Methylsulfinyl)phenyl)-5-(5-(trifluoromethyl)isoxazol-3-yl)thiophene-2-sulfonamide (**3aj**)

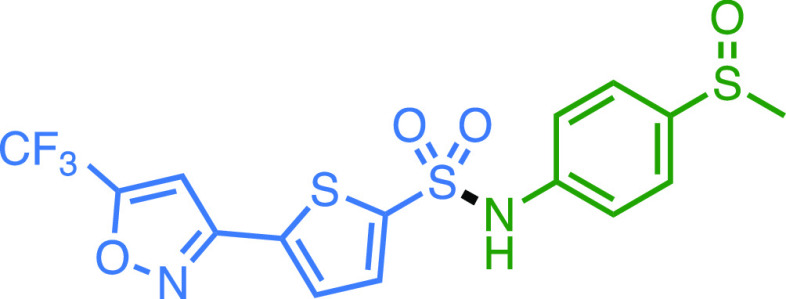



Following General Procedure 3 on a 0.2 mmol scale, flash column chromatography (0–2% MeOH in DCM) afforded **3aj** as a white crystalline solid (93 mg, 53%). ^1^H NMR (500 MHz, DMSO-*d*_6_): δ 11.11 (s, 1H), 8.12 (q, *J* = 1.0 Hz, 1H), 7.84 (d, *J* = 4.0 Hz, 1H), 7.76 (d, *J* = 4.0 Hz, 1H), 7.66–7.57 (m, 2H), 7.39–7.31 (m, 2H), 2.68 (s, 3H).^13^C{^1^H} NMR (126 MHz, DMSO-*d*_6_): δ 157.7 (q, *J* = 42.2 Hz), 157.4, 142.6, 141.5, 139.6, 133.4, 133.2, 130.8, 125.2, 120.3, 117.6 (q, *J* = 270.2 Hz), 106.0, 43.01. ^19^F{^1^H} NMR (376 MHz, CDCl_3_): δ −63.2. mp 222–224 °C. IR: ν_max_, 3134, 3098, 3046, 2924, 2867, 1338, 1316, 1151 cm^–1^. HRMS: *m*/*z* calculated for C_15_H_11_F_3_N_2_O_4_S_3_^+^, 436.9906 [M + H]^+^. Found *m*/*z*, 436.9897, Δ = −2.0 ppm.

## Data Availability

The data underlying this study are available in the published article and its Supporting Information.

## Supporting Information Available

Details of the experimental setup, optimization data, and NMR spectra (PDF)
