# Supplementary material for: Nitro-sulfinate Reductive Coupling to Access (Hetero)aryl Sulfonamides
Source: J Org Chem. 2024 Jan 19;89(3):1898–909. doi: 10.1021/acs.joc.3c02557 (PMC10845164; doi:10.1021/acs.joc.3c02557)
Supplement: Supplementary file 1 — jo3c02557_si_001.pdf [file jo3c02557_si_001.pdf]

Supporting Information

Nitro-~Sulfinate Reductive Coupling to Access (Hetero)aryl Sulfonamides

Sandra E. Gatarz,[a] Oliver M. Griffiths,[a] Henrique A. Esteves,[a] Wenhua Jiao,[b] Peter Morse,[b] Ethan L. Fisher,[b] David C. Blakemore[b] and Steven V. Ley[a]*

[a] *Yusuf Hamied Department of Chemistry, University of Cambridge, Cambridge CB2 1EW, United Kingdom*

[b] *Medicine Design, Pfizer, Inc. Groton, Connecticut 06340, USA*

E-mail: svl1000@cam.ac.uk

Table of contents

1. S_NAr Side-Reactions and Data 2

2. Control experiments for mechanism investigation 3

3. ^{1}H, ^{13}C{^{1}H} and ^{19}F{^{1}H} NMR Spectra 5

1. S$_N$Ar Side-Reactions and Data

One limitation in the reaction scope under the sodium bisulfite reducing conditions regarding the nitroarene was observed with chloro-substituted nitroarenes. This was due to S$_N$Ar side-reactivity between arylsulfinates and chloro-substituted nitroarene yielding sulfones instead of the intended sulfonamides. Most sulfones were identified by ^{19}F NMR and LCMS. 2 examples were isolated and characterised as shown below.

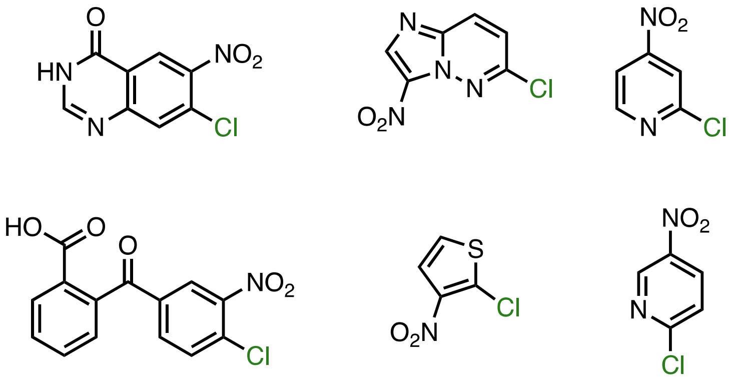

Figure S1. Chloro-substituted nitroarenes that were observed to undergo S$_N$Ar side-reactions.

Isolated S$_N$Ar by-products

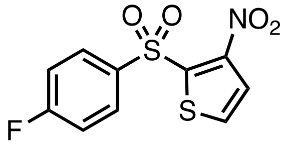

*2-((4-Fluorophenyl)sulfonyl)-3-nitrothiophene (**S1**).* Following General Procedure 1. Purification by flash column chromatography (0 – 20% EtOAc in petroleum ether) yielded **S1** as a yellow solid (129 mg, 89%). ^{1}H NMR (400 MHz, DMSO-d_6) δ 8.24 (d, J = 5.5 Hz, 1H), 8.16 – 8.06 (m, 2H), 7.80 (d, J = 5.5 Hz, 1H), 7.59 – 7.49 (m, 2H). ^{13}C{^{1}H} NMR (101 MHz, DMSO-d_6) δ 165.44 (d, J = 254.8 Hz), 145.9, 140.6, 135.31 (d, J = 2.8 Hz), 134.7, 131.80 (d, J = 10.1 Hz), 126.4, 117.00 (d, J = 23.3 Hz). ^{19}F{^{1}H} NMR (376 MHz, DMSO-d_6) δ −103.8. m.p. 138 – 140 °C. ν_{max} 1323, 1142 cm^{-1}. HRMS: m/z calculated for $C_{10}H_7NO_4S_2F^+$, 287.9801 [M+H]$^+$. Found m/z 287.9806, Δ = 1.7 ppm.

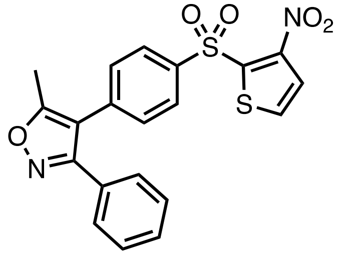

*5-Methyl-4-(4-((3-nitrothiophen-2-yl)sulfonyl)phenyl)-3-phenylisoxazole (**S2**).* Following General Procedure 1. Purification by flash column chromatography (0 – 100% $CHCl_3$ in petroleum ether) yielded **S2** as a white solid (65 mg, 32%). ^{1}H NMR (400 MHz, DMSO-d_6) δ 8.26 (d, J = 5.5 Hz, 1H), 8.06 – 8.01 (m, 2H), 7.81 (d, J = 5.5 Hz, 1H), 7.55 – 7.36 (m, 5H), 7.35 – 7.28 (m, 2H), 2.48 (s, 3H). ^{13}C{^{1}H} NMR (101 MHz, DMSO-d_6) δ 168.0, 160.7, 145.9, 140.4, 138.0, 136.2, 134.7, 130.5, 129.9, 128.9, 128.7, 128.2, 128.1, 126.3, 113.9, 11.5. m.p. 190 – 192 °C. HRMS: m/z calculated for $C_{20}H_{15}N_2O_5S_2^+$, 427.0422 [M+H]$^+$. Found m/z 427.0442, Δ = 4.7 ppm.

2. Control experiments for mechanism investigation

The monomeric state of nitrosobenzene used for control experiments in 0.2 M solution of DMSO-d_6 at room temperature was confirmed by ^{1}H NMR. Additionally, nitrosobenzene in DMSO-d_6 gave a characteristic blue-green solution indicating monomer formation (Figure S2).

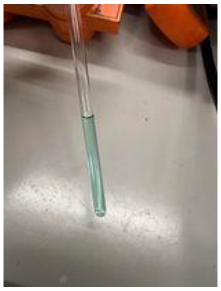

Figure S2. A picture of nitrosobenzene (0.2 M) in DMSO-d_6 in an NMR tube.

Reaction with nitrosobenzene

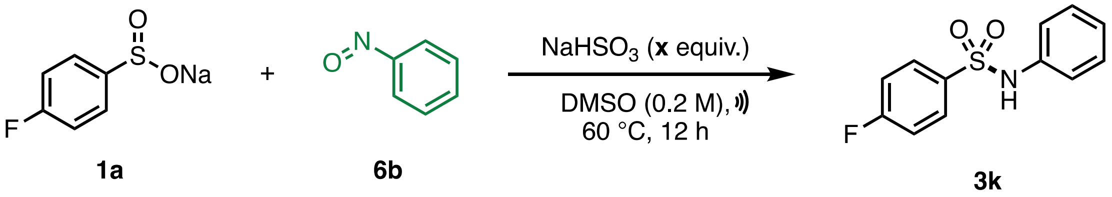

Entry	$NaHSO_3$	Note	NMR Yield[a]
1	1 eq	-	5%
2	2 eq	-	14%
3	3.5 eq	-	2%
4	3.5 eq	PhNO added portionwise over 1 h	72%

Reaction conditions: **1a** (1.0 mmol), **6b** (0.5 mmol), DMSO (2.5 mL), 60 °C, 12 h, under nitrogen. [a]NMR yield determined by $^{19}F\{^1H\}$ NMR against internal standard $PhCF_3$.

Reaction with N-phenylhydroxylamine

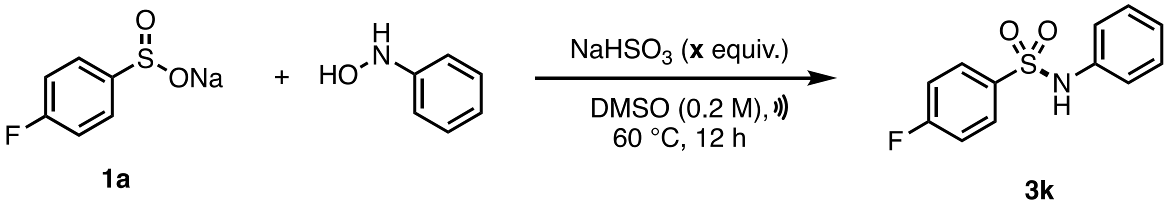

Entry	$NaHSO_3$	Note	NMR Yield[a]
1	1 eq		10%
2	2 eq		15%
3	3.5 eq		10%
4	3.5 eq	ArNHOH added portionwise over 1 h	10%

Reaction conditions: x (1.0 mmol), x (0.5 mmol), DMSO (2.5 mL), 60 °C, 12 h, under nitrogen. [a]NMR yield determined by $^{19}F\{^1H\}$ NMR against internal standard $PhCF_3$.

Reaction with aniline

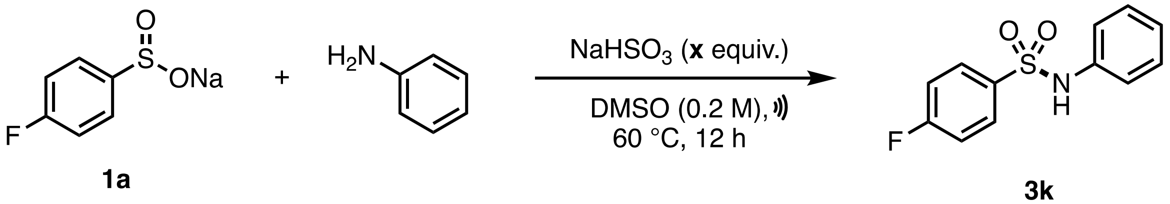

Entry	$NaHSO_3$	NMR Yield[a]
1	1 eq	4%
2	2 eq	8%
3	3.5 eq	15%
4	-	no reaction occurred

Reaction conditions: x (1.0 mmol), x (0.5 mmol), DMSO (2.5 mL), 60 °C, 12 h, under nitrogen. [a]NMR yield determined by $^{19}F\{^1H\}$ NMR against internal standard $PhCF_3$.

3. ^{1}H, $^{13}C\{^{1}H\}$ and $^{19}F\{^{1}H\}$ NMR Spectra

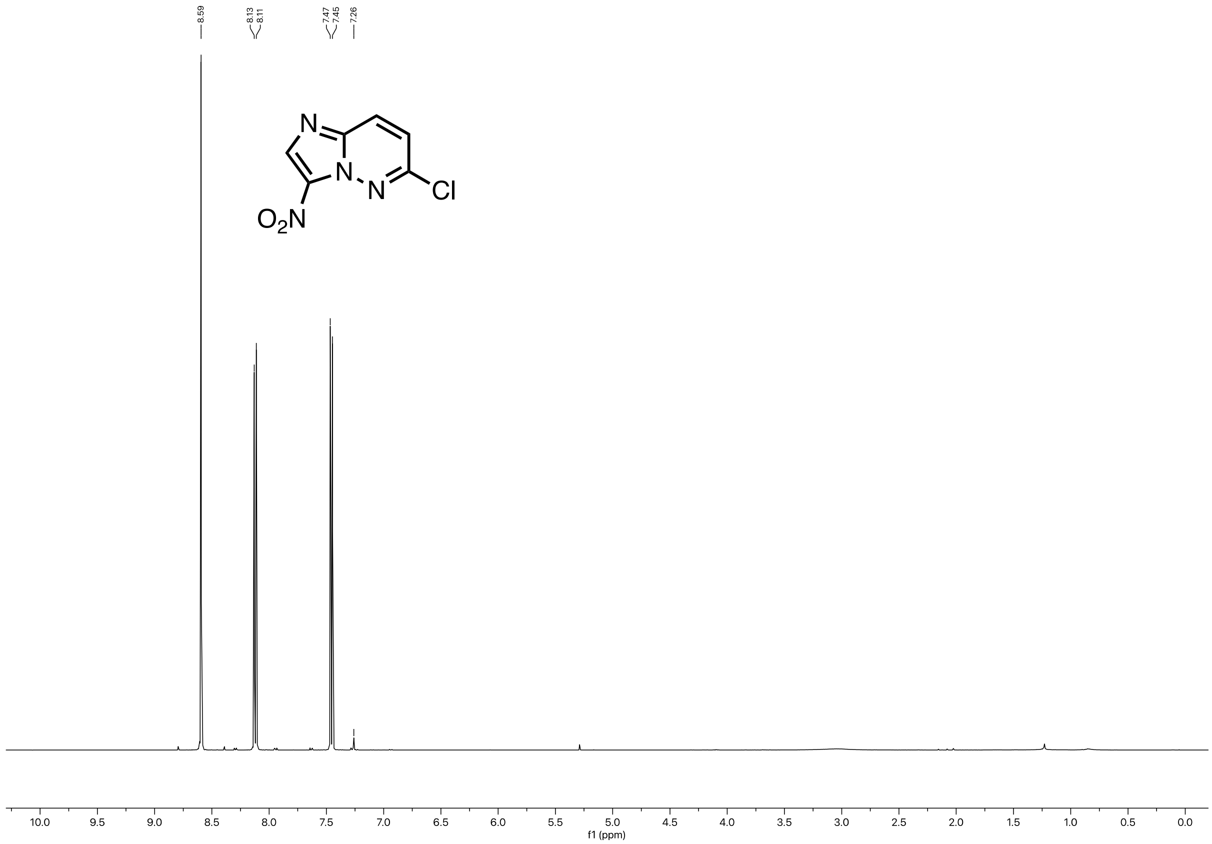

^{1}H NMR of **2ah** (500 MHz, $CDCl_3$).

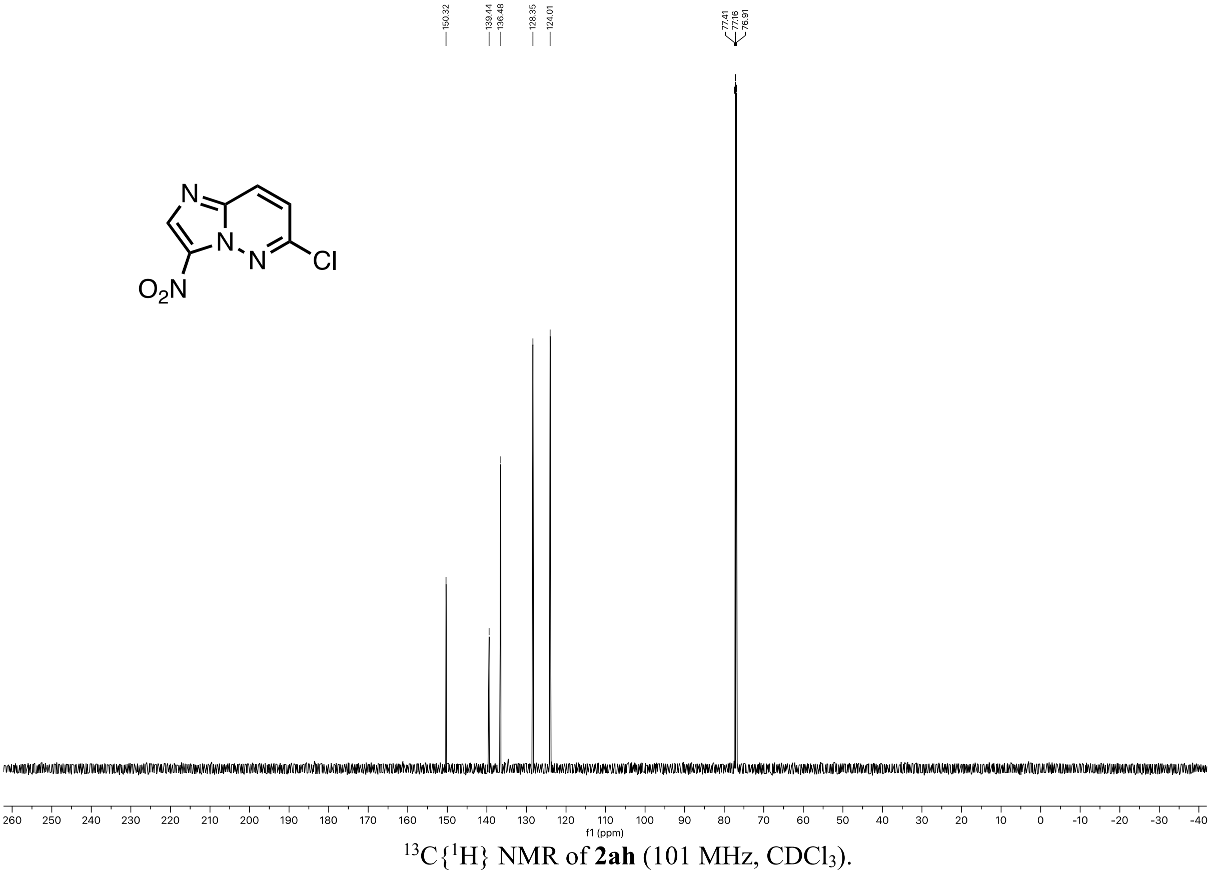

$^{13}C\{^1H\}$ NMR of **2ah** (101 MHz, $CDCl_3$).

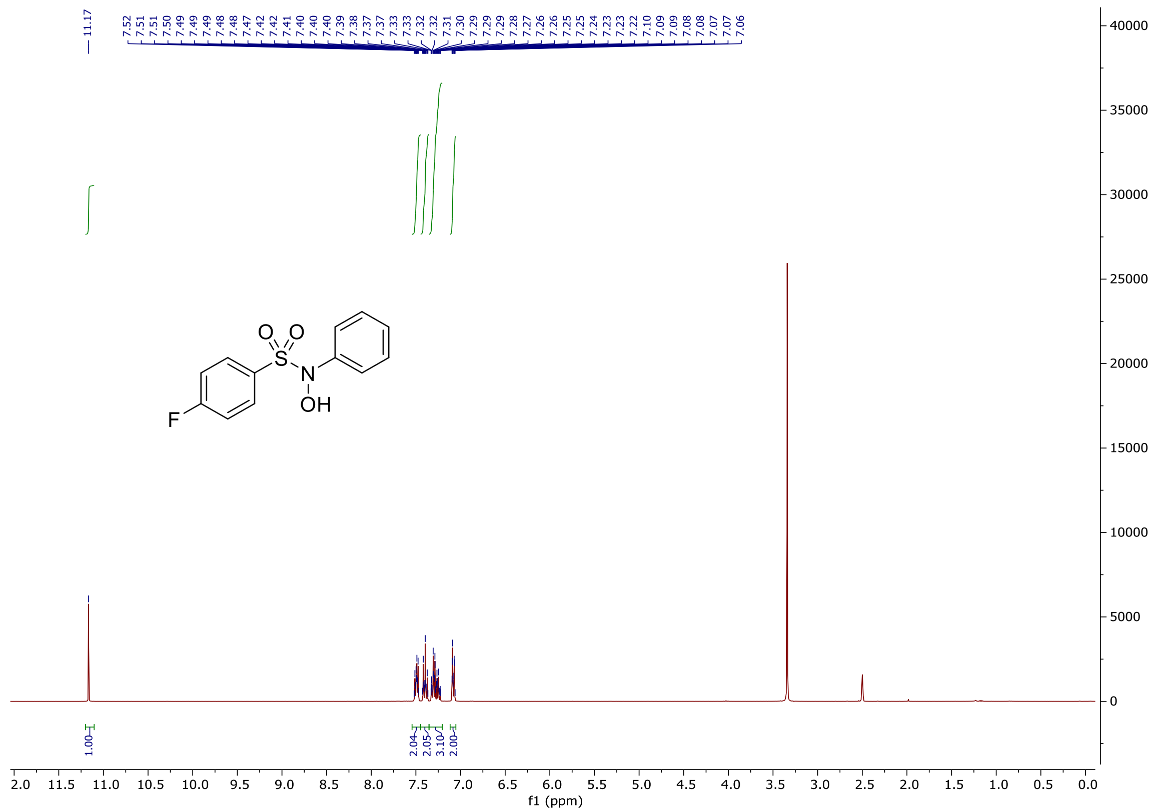

^{1}H NMR of **7** (400 MHz, DMSO-d_6).

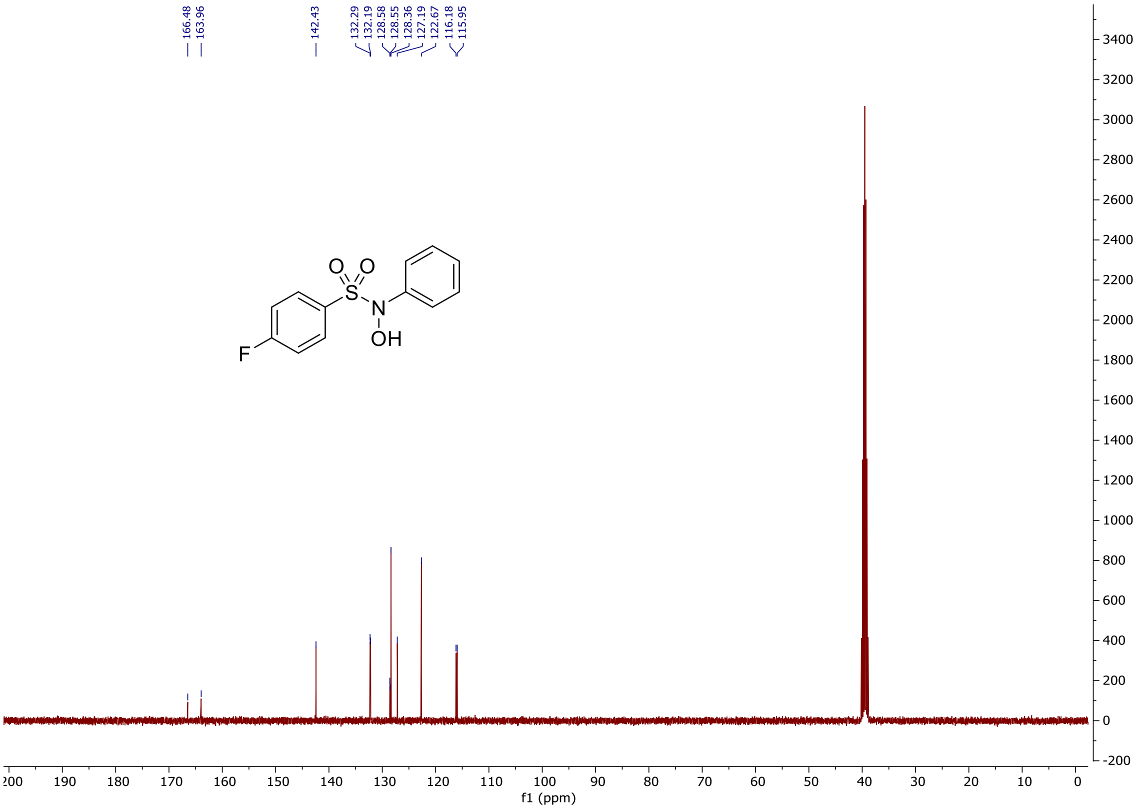

^{13}C{^{1}H} NMR of **7** (101 MHz, DMSO-d_6).

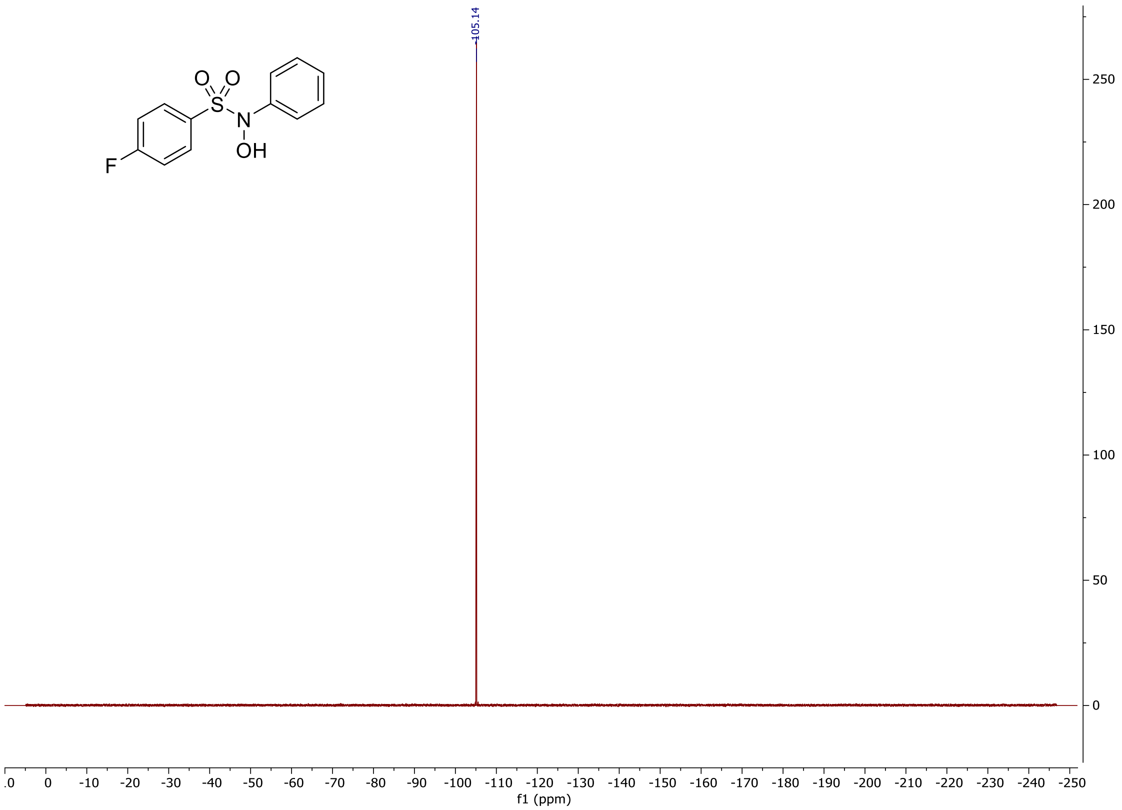

$^{19}F\{^1H\}$ NMR of **7** (376 MHz, DMSO-d_6).

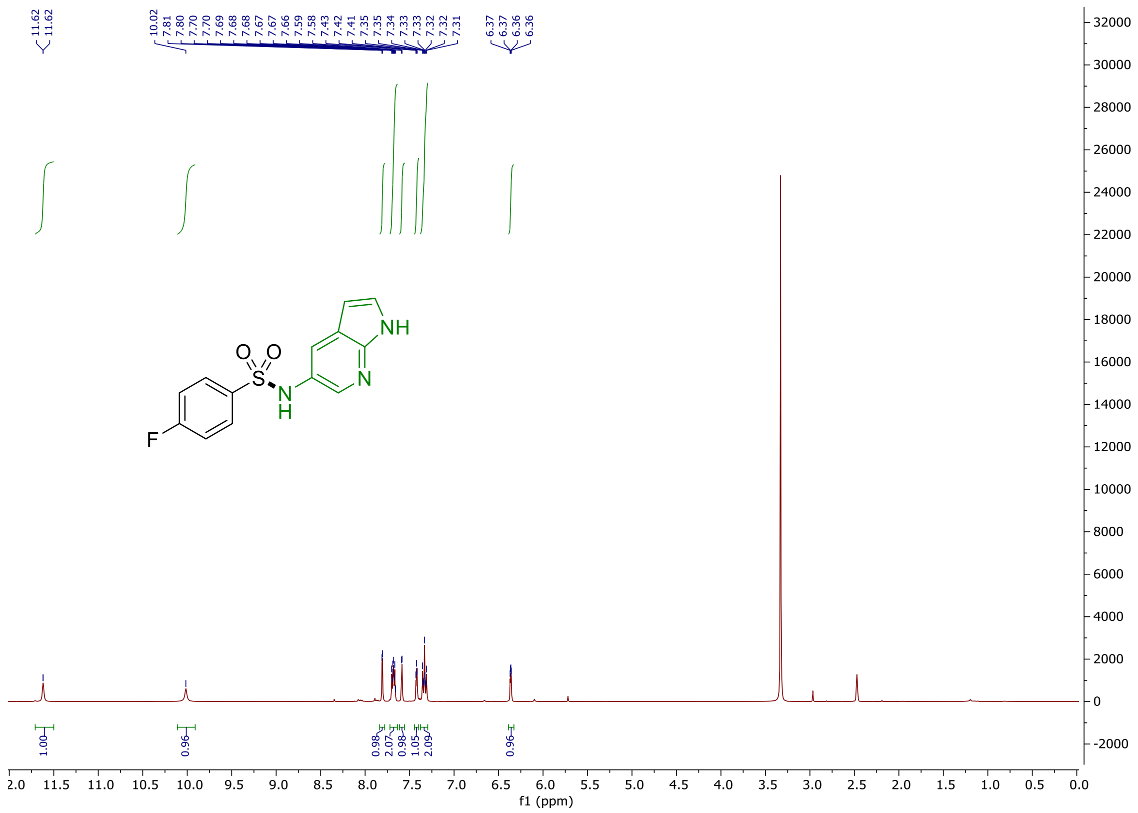

^1H NMR of **3b** (400 MHz, DMSO-d_6)

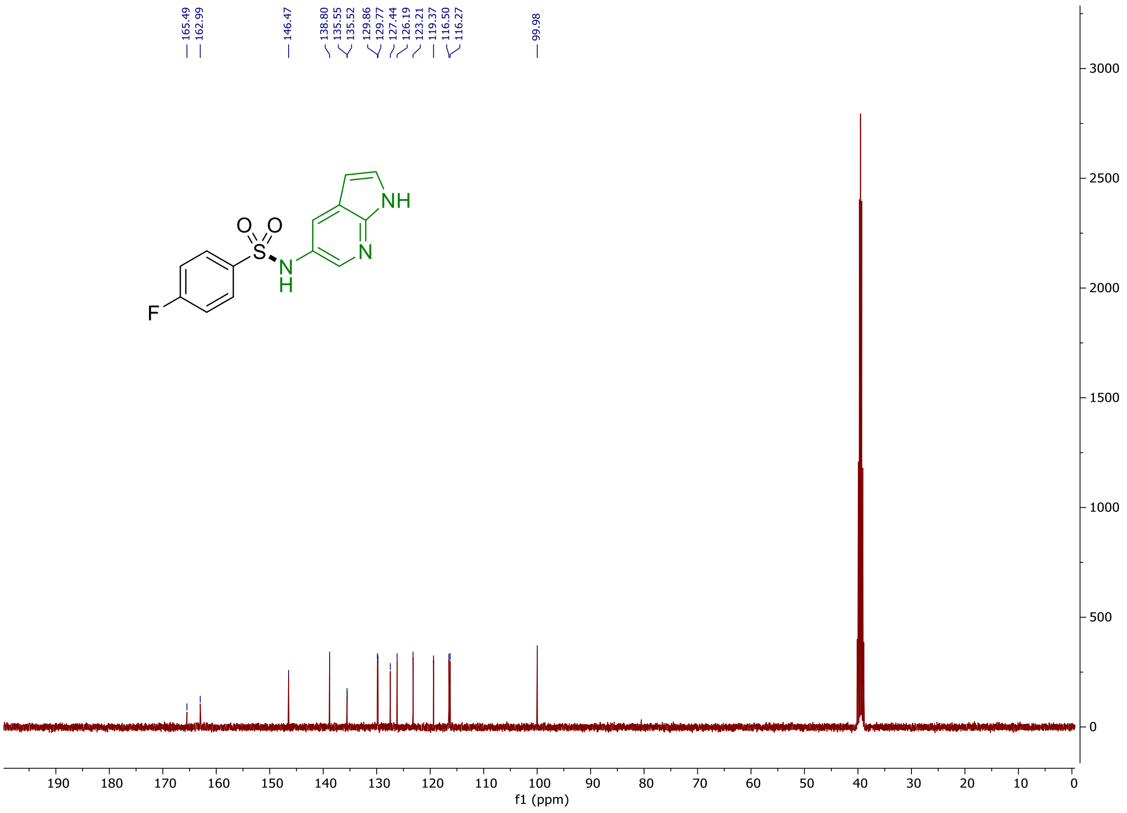

^{13}C NMR of **3b** (101 MHz, DMSO-d_6)

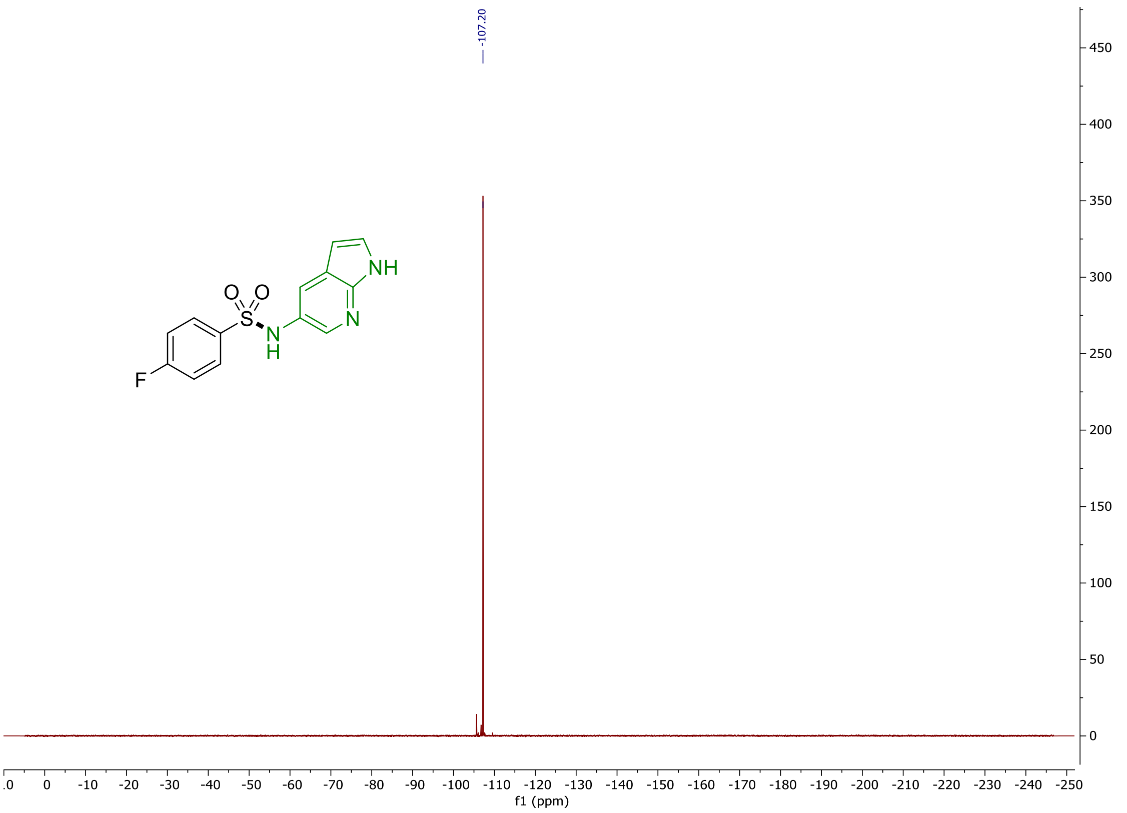

^{19}F NMR of **3b** (376 MHz, DMSO-d_6)

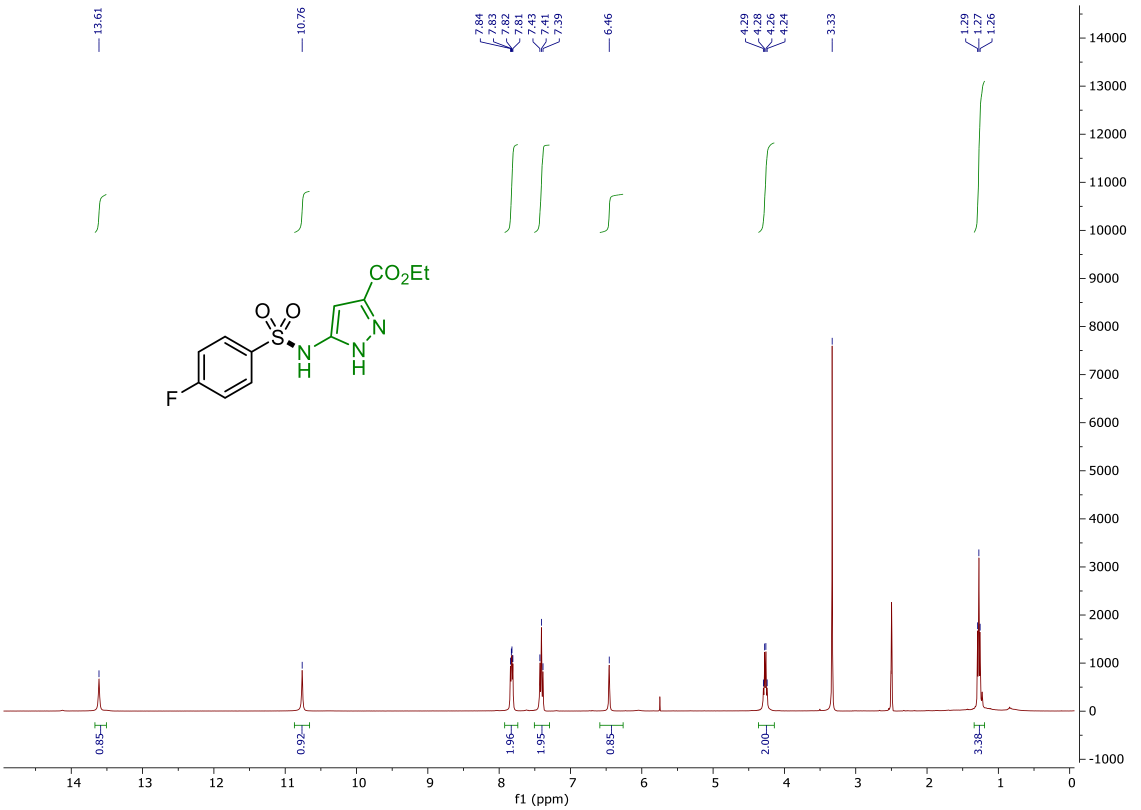

^{1}H NMR of **3c** (400 MHz, DMSO-d$_6$)

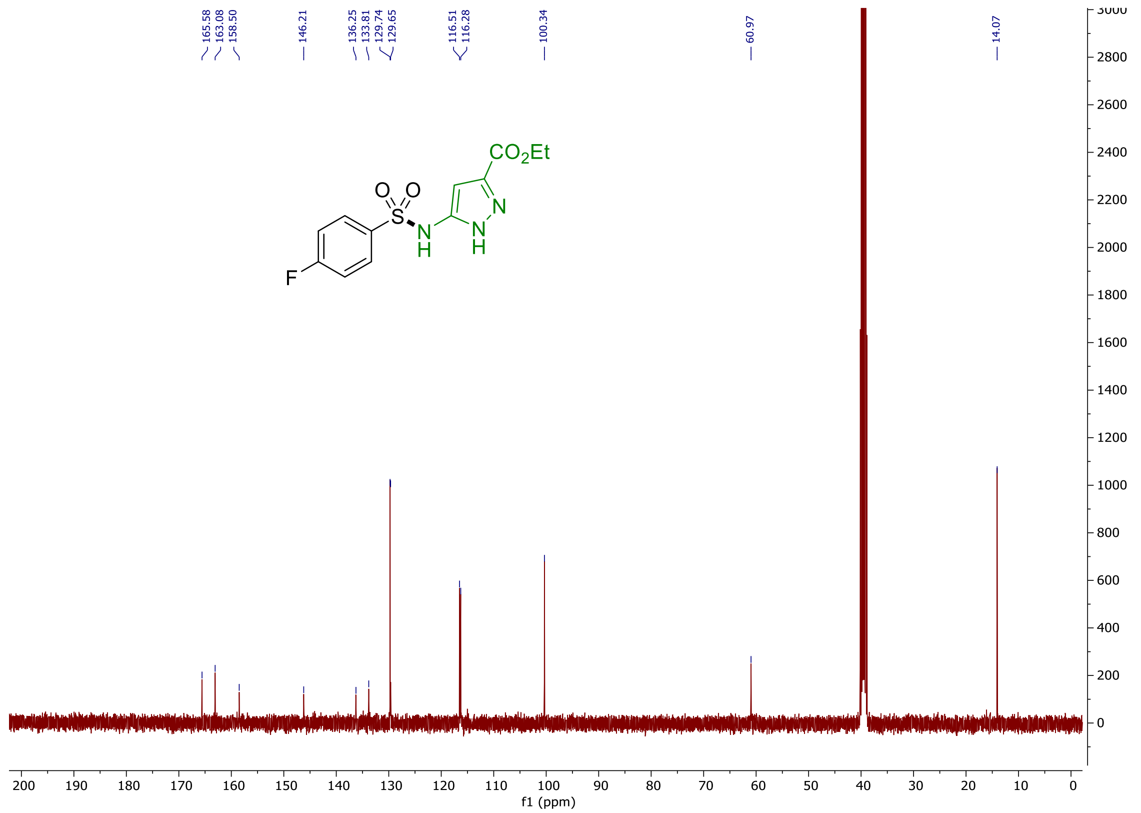

^{13}C NMR of **3c** (101 MHz, DMSO-d_6)

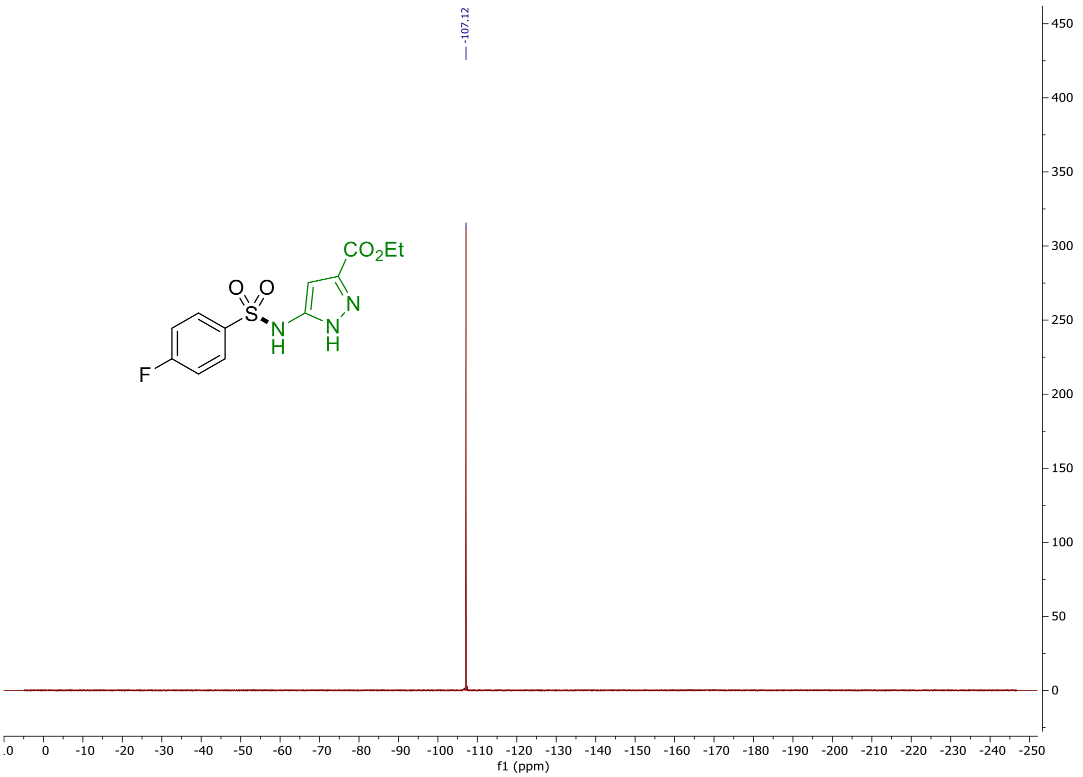

^{19}F NMR of **3c** (376 MHz, DMSO-d_6)

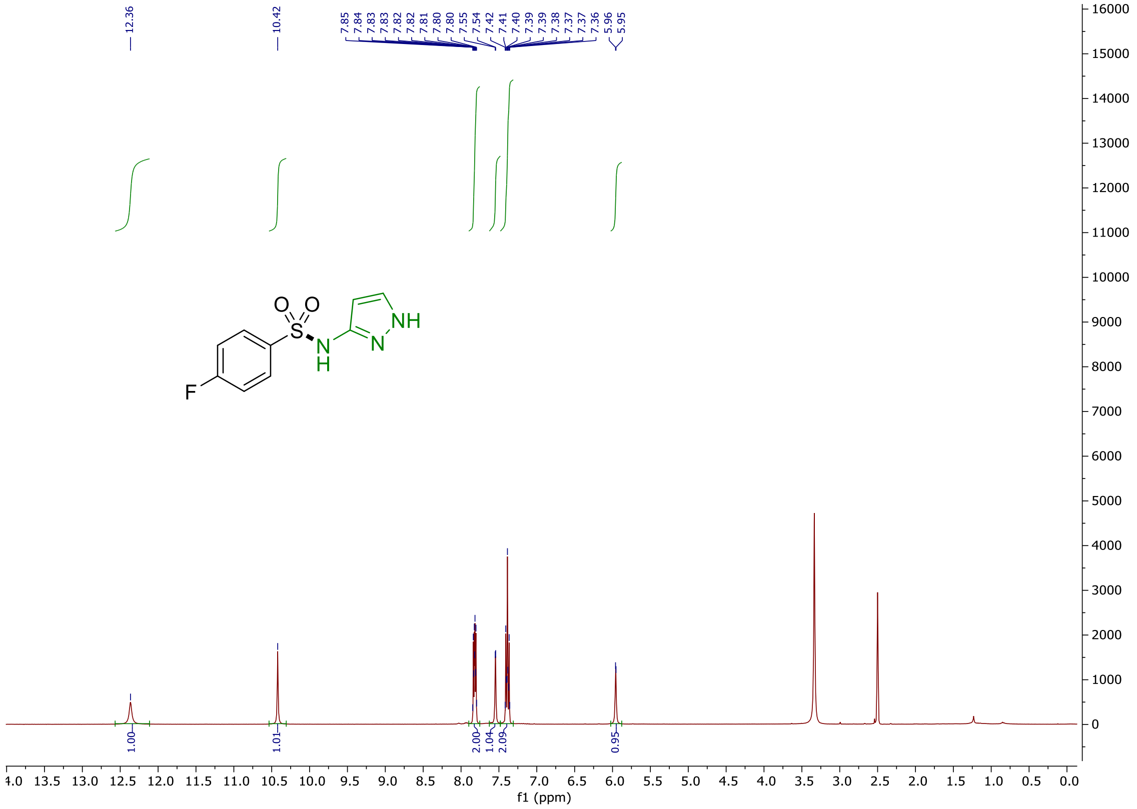

^{1}H NMR of **3d** (400 MHz, DMSO-d_6)

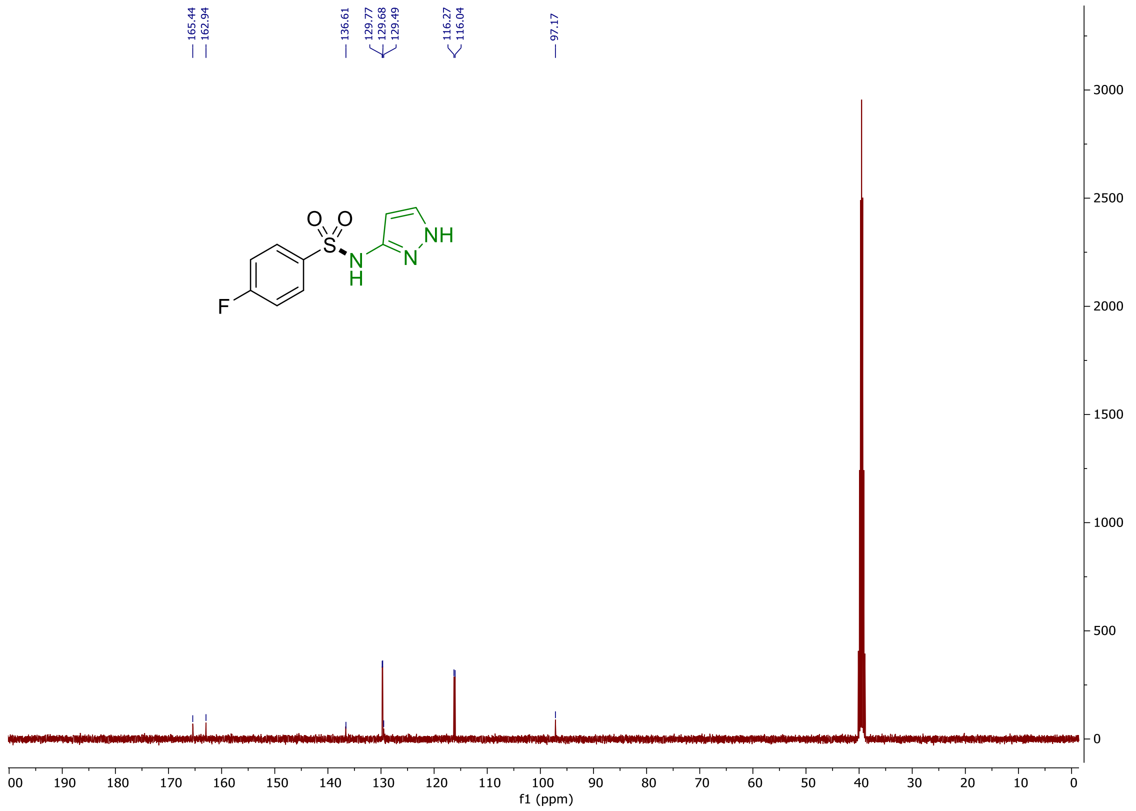

^{13}C NMR of **3d** (101 MHz, DMSO-d_6)

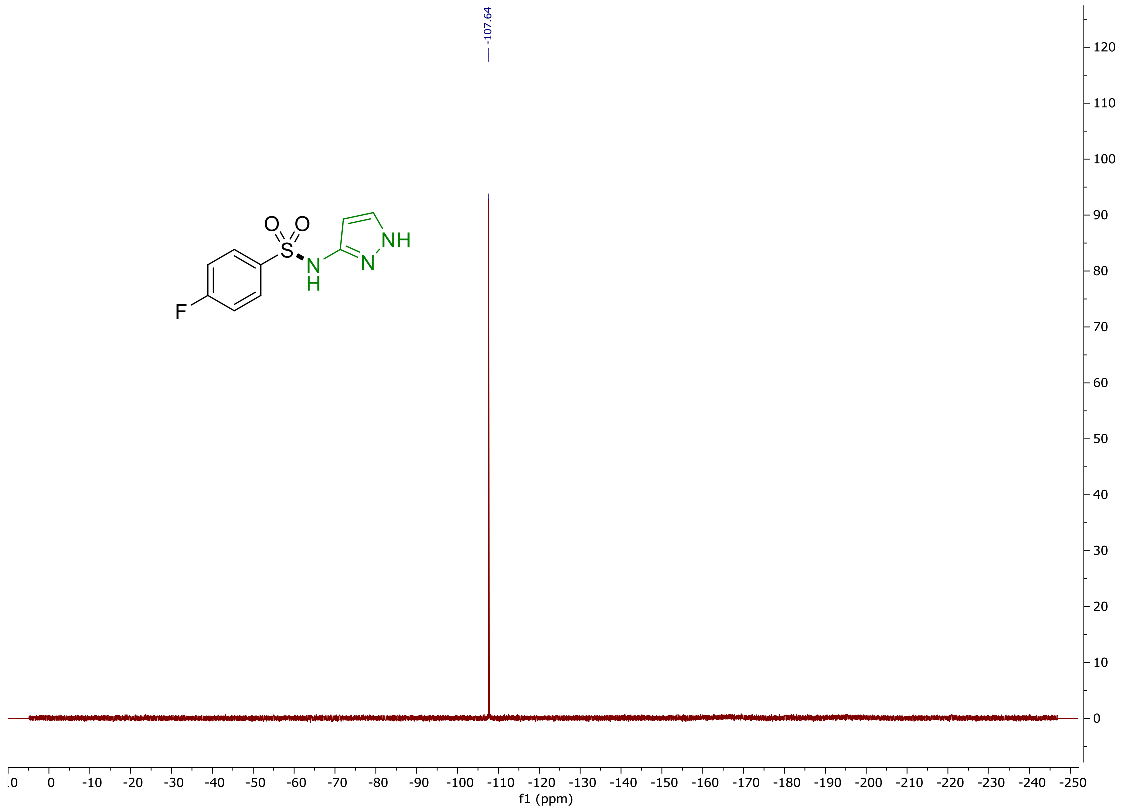

^{19}F NMR of **3d** (376 MHz, DMSO-d_6)

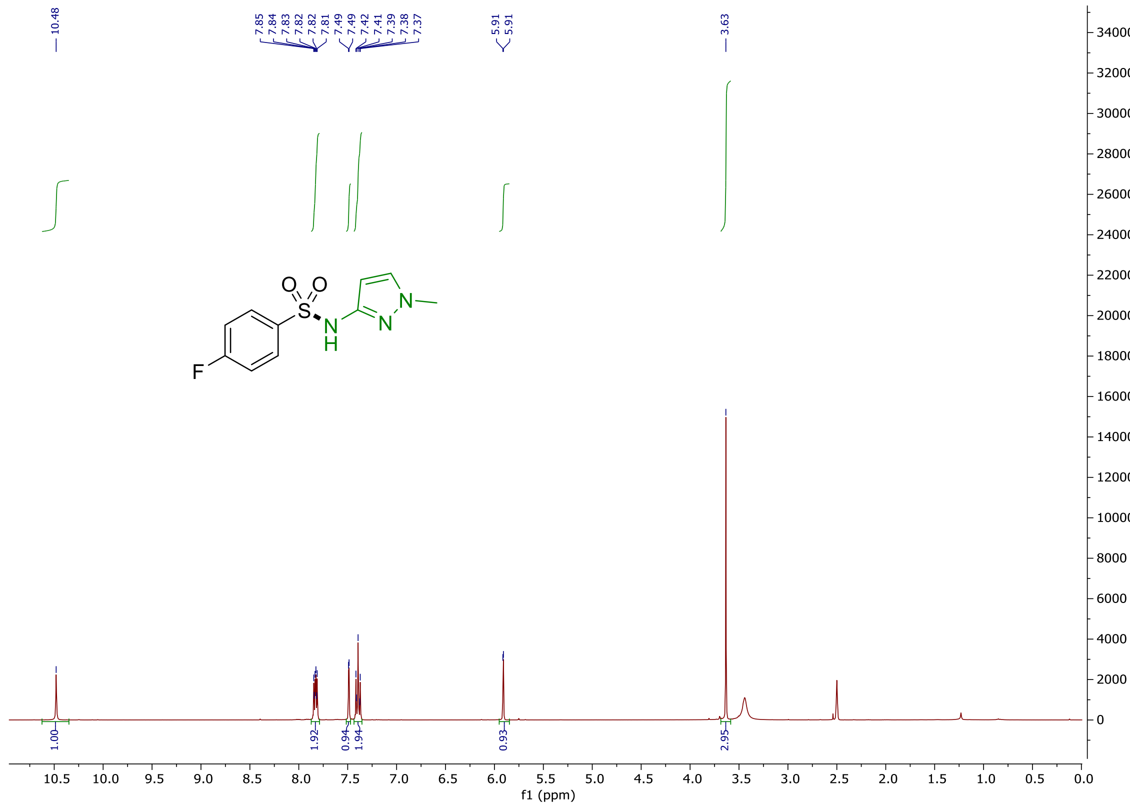

^{1}H NMR of **3e** (400 MHz, DMSO-d_6)

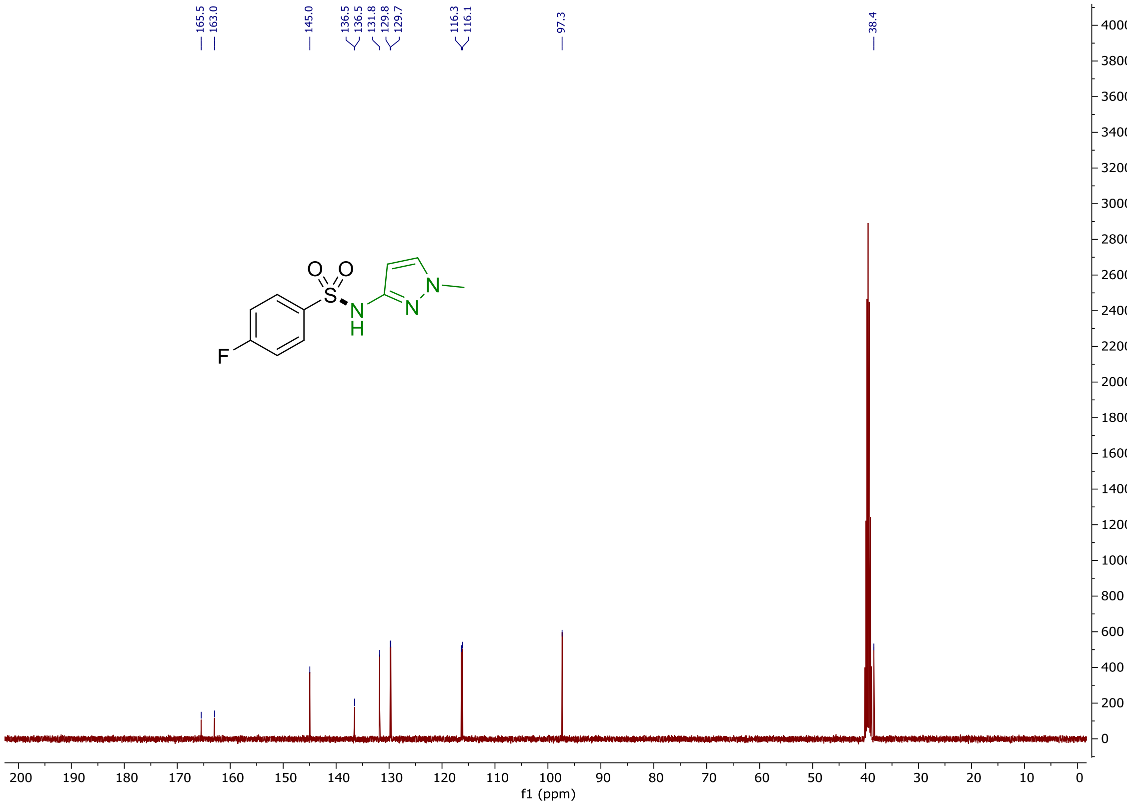

^{13}C NMR of **3e** (101 MHz, DMSO-d_6)

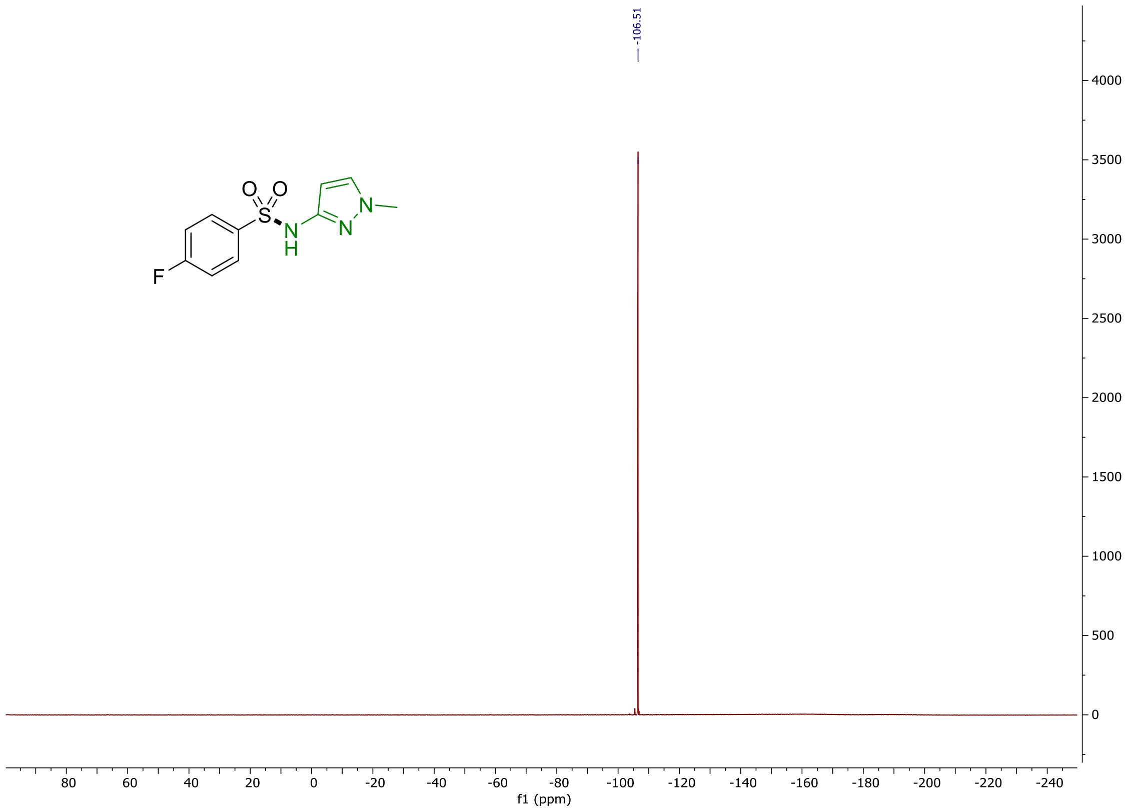

^{19}F NMR of **3e** (376 MHz, DMSO-d_6)

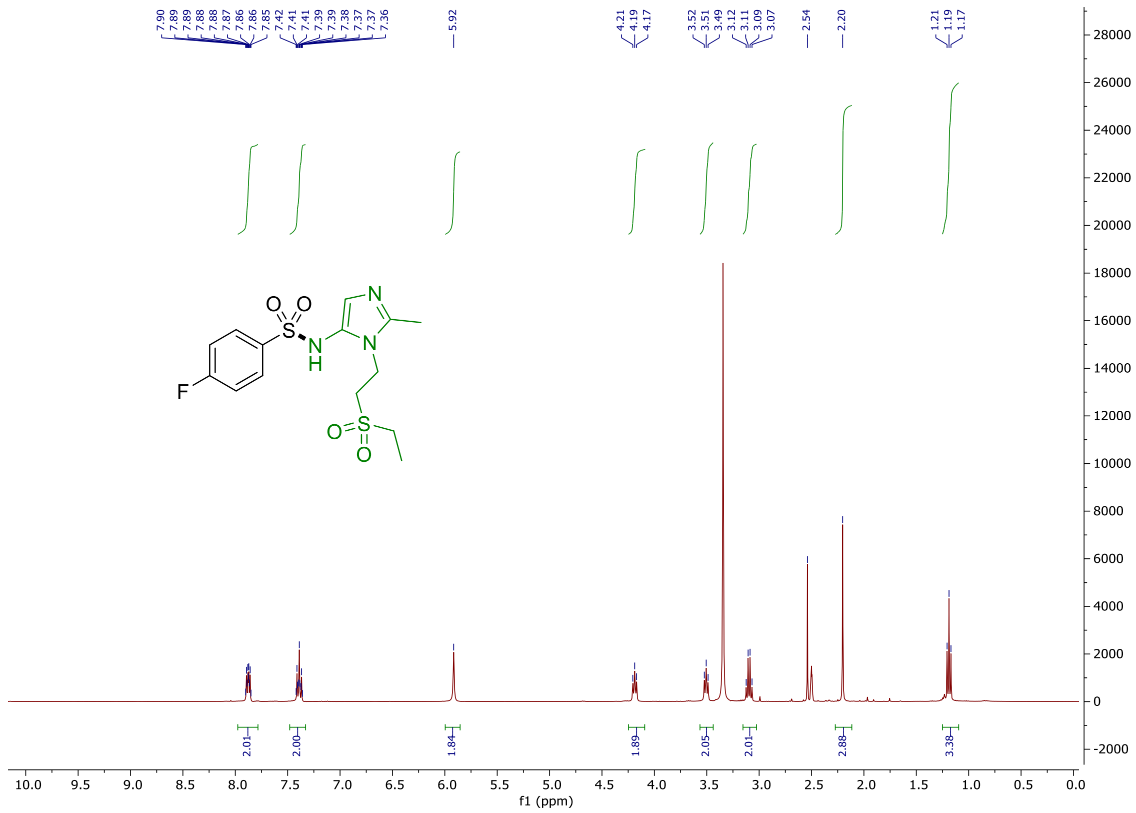

^{1}H NMR of **3f** (400 MHz, DMSO-d_6)

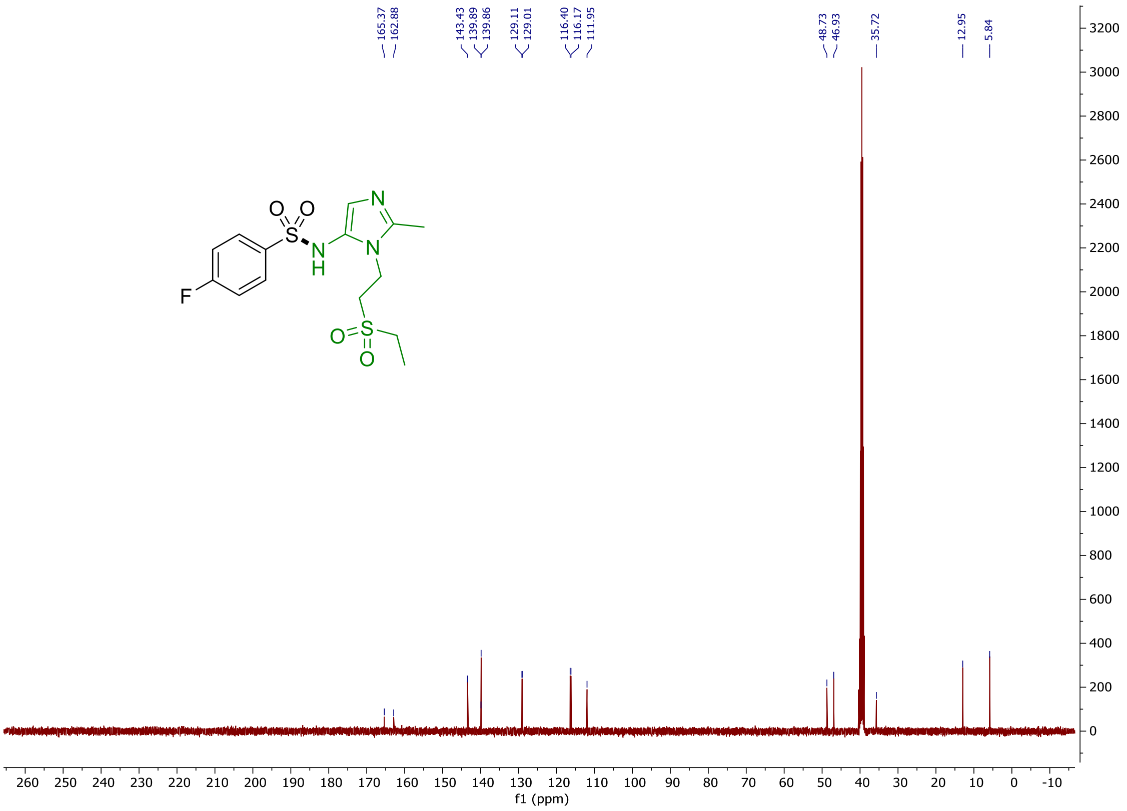

^{13}C NMR of **3f** (101 MHz, DMSO-d_6)

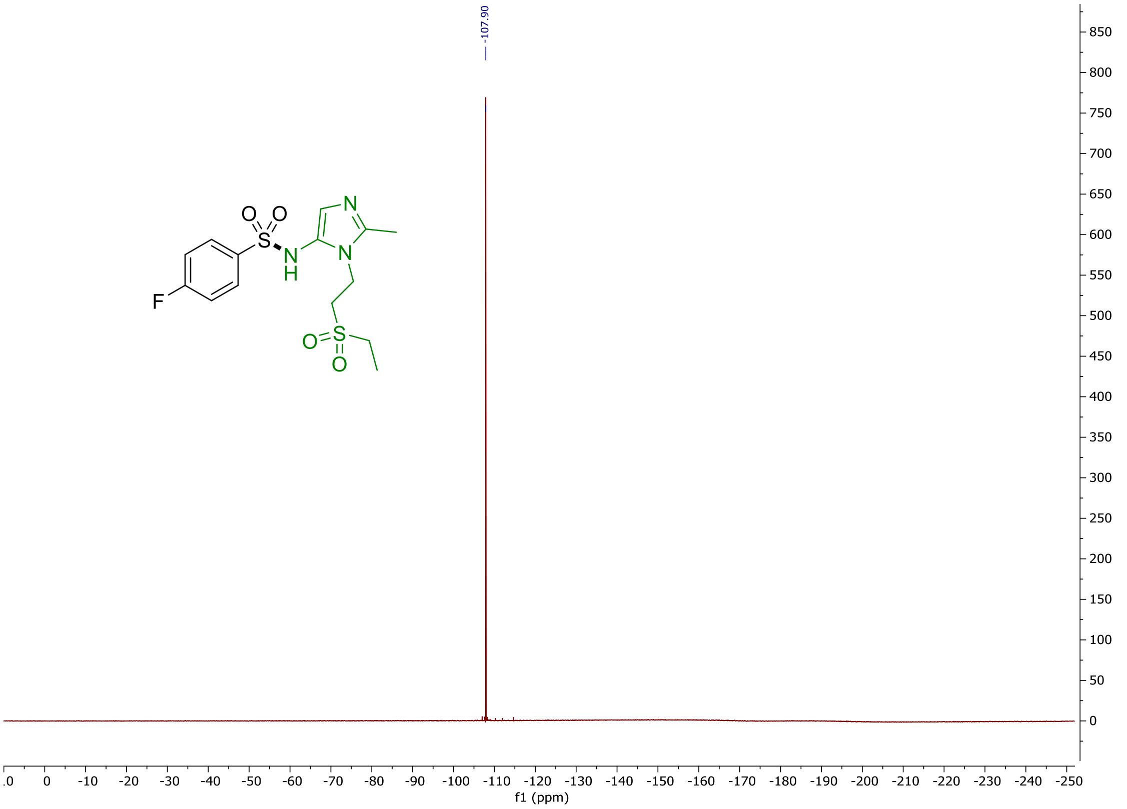

^{19}F NMR of **3f** (376 MHz, DMSO-d_6)

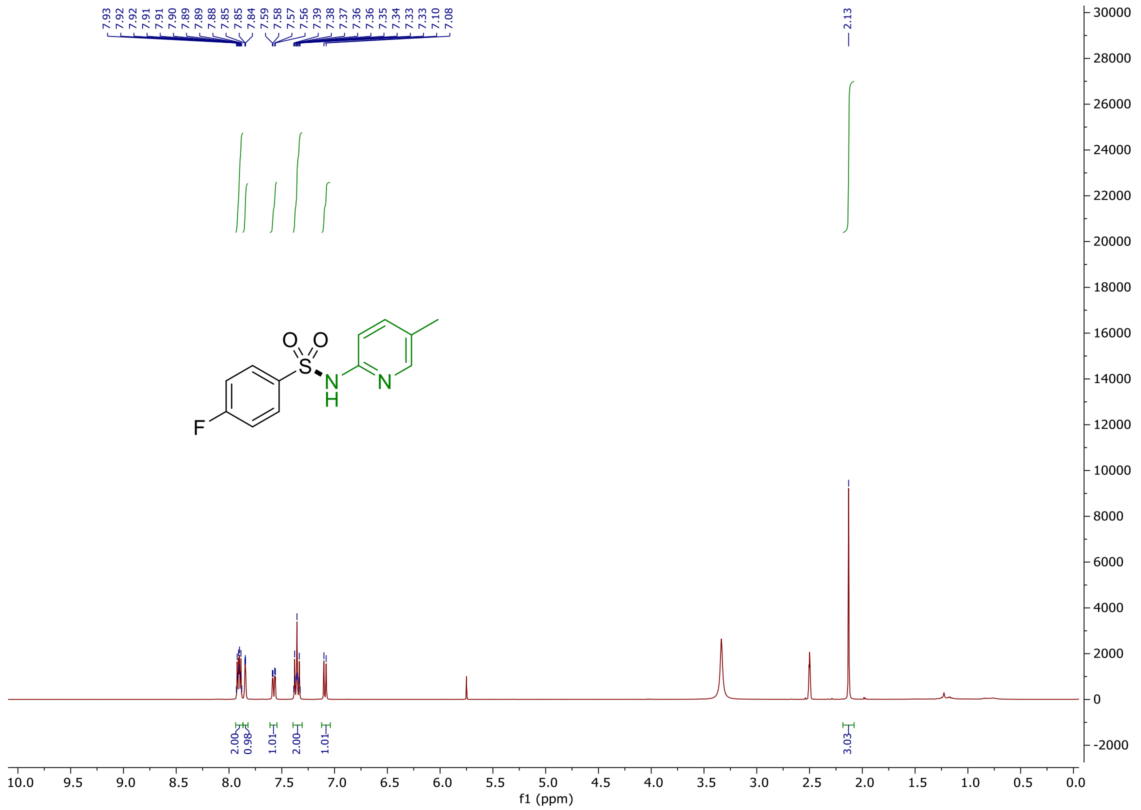

^{1}H NMR of **3g** (400 MHz, DMSO-d_6)

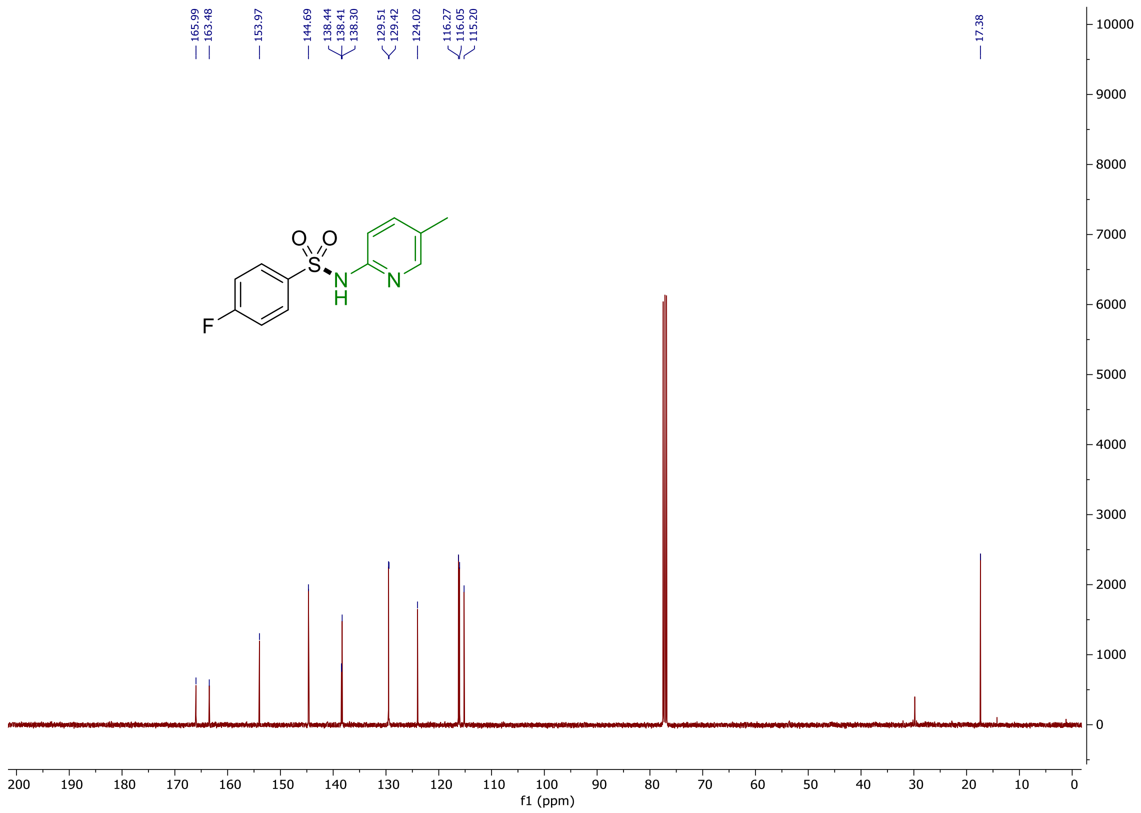

^{13}C NMR of **3g** (101 MHz, DMSO-d_6)

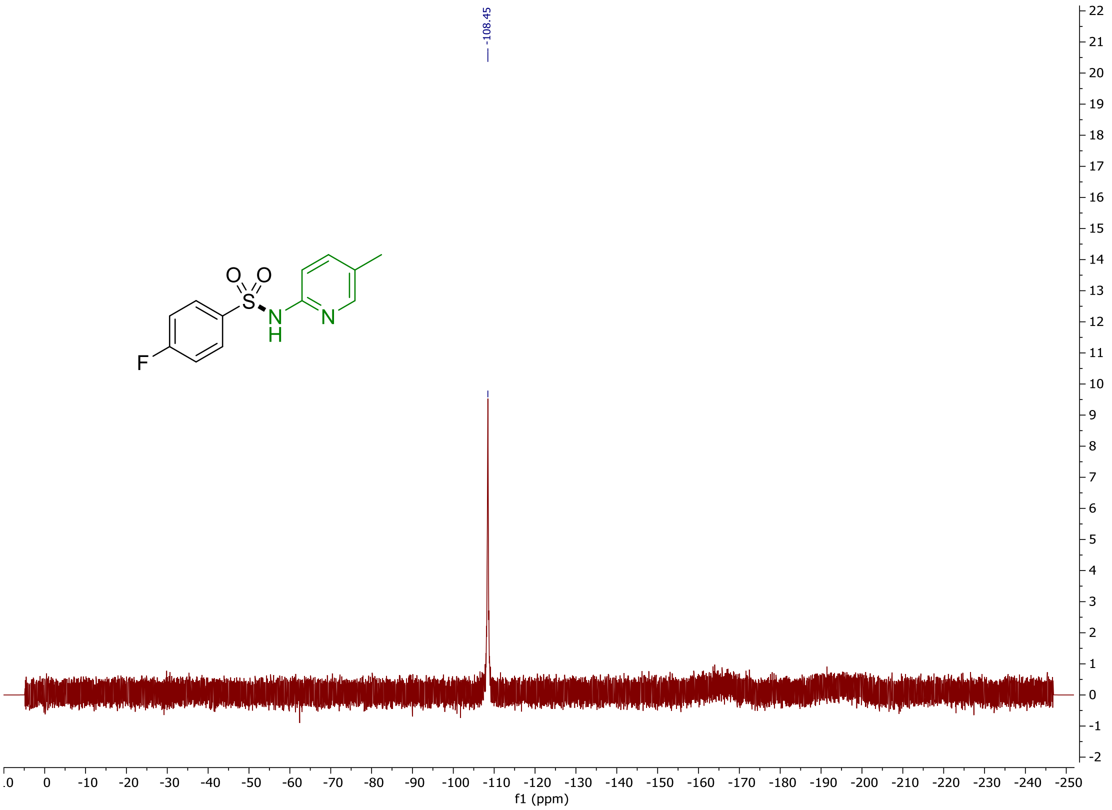

^{19}F NMR of **3g** (376 MHz, DMSO-d_6)

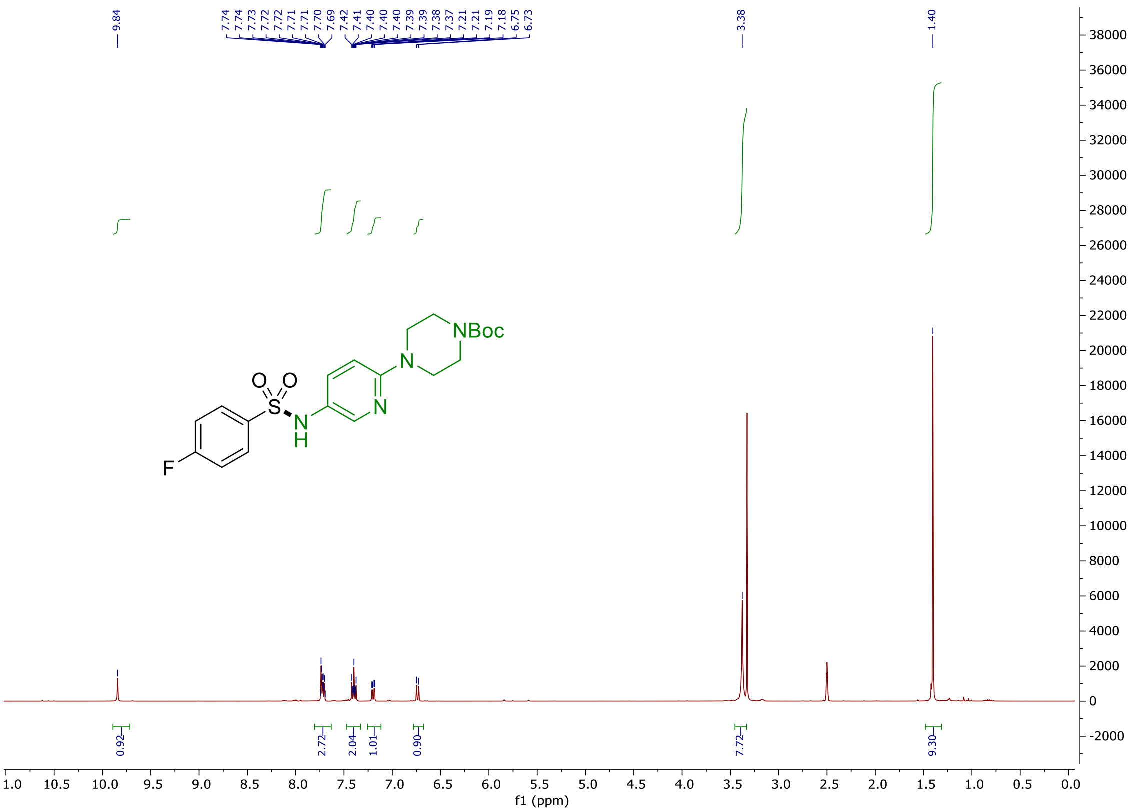

^{1}H NMR of **3h** (400 MHz, DMSO-d_6)

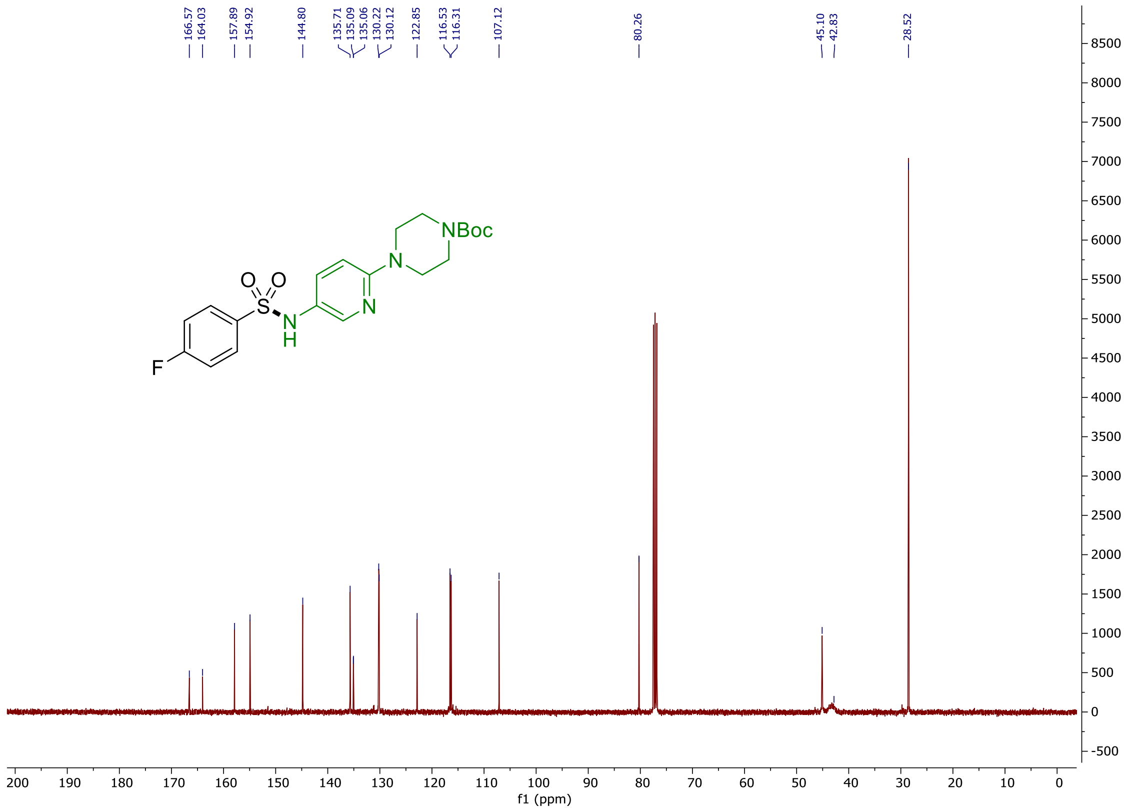

^{13}C NMR of **3h** (101 MHz, DMSO-d_6)

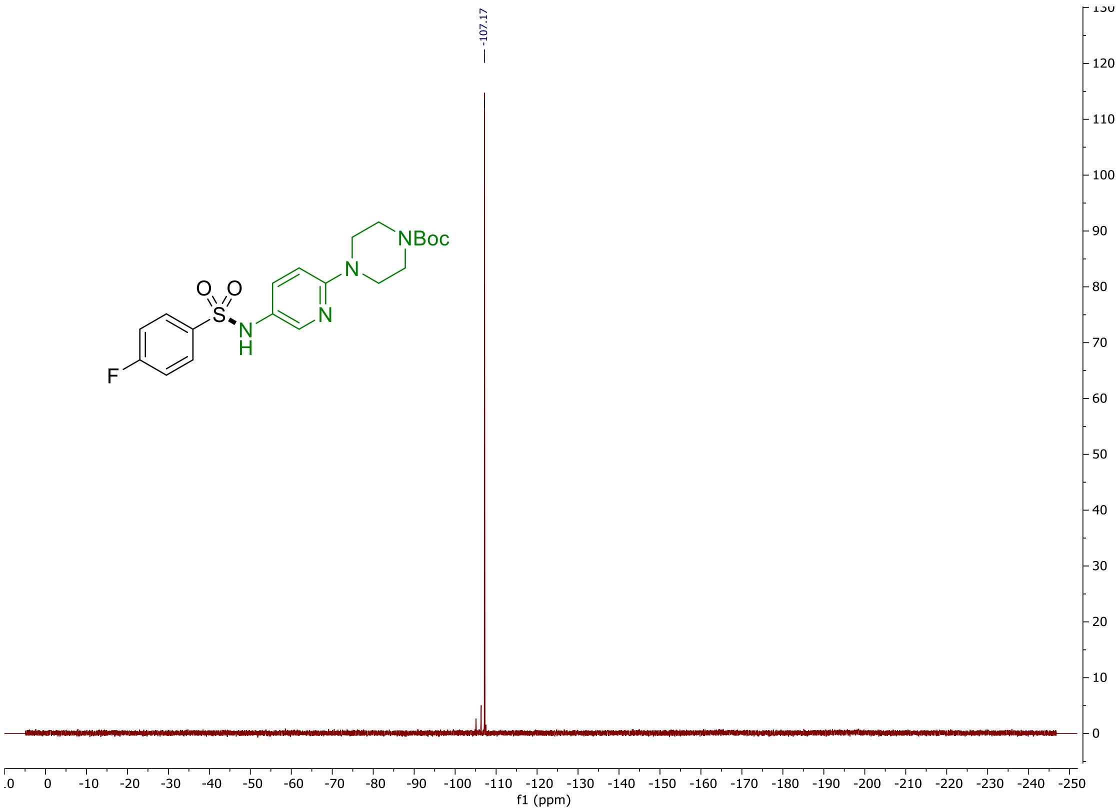

^{19}F NMR of **3h** (376 MHz, DMSO-d_6)

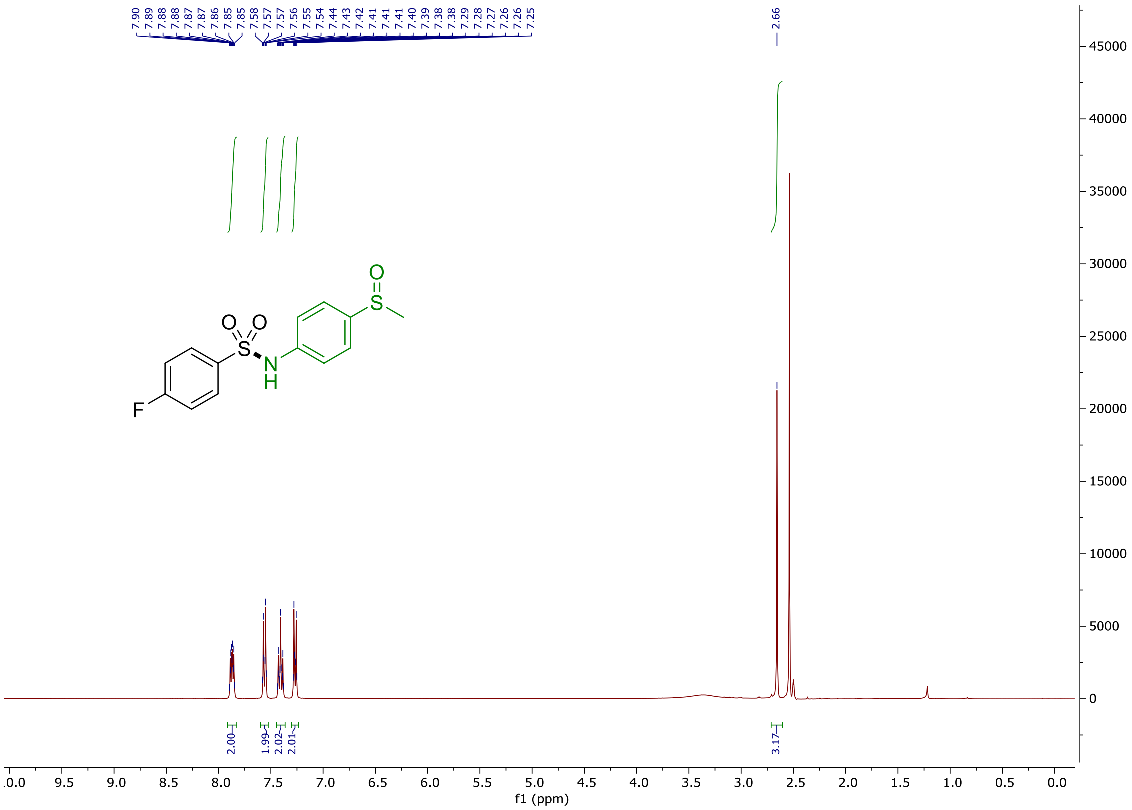

^{1}H NMR of **3i** (400 MHz, DMSO-d_6)

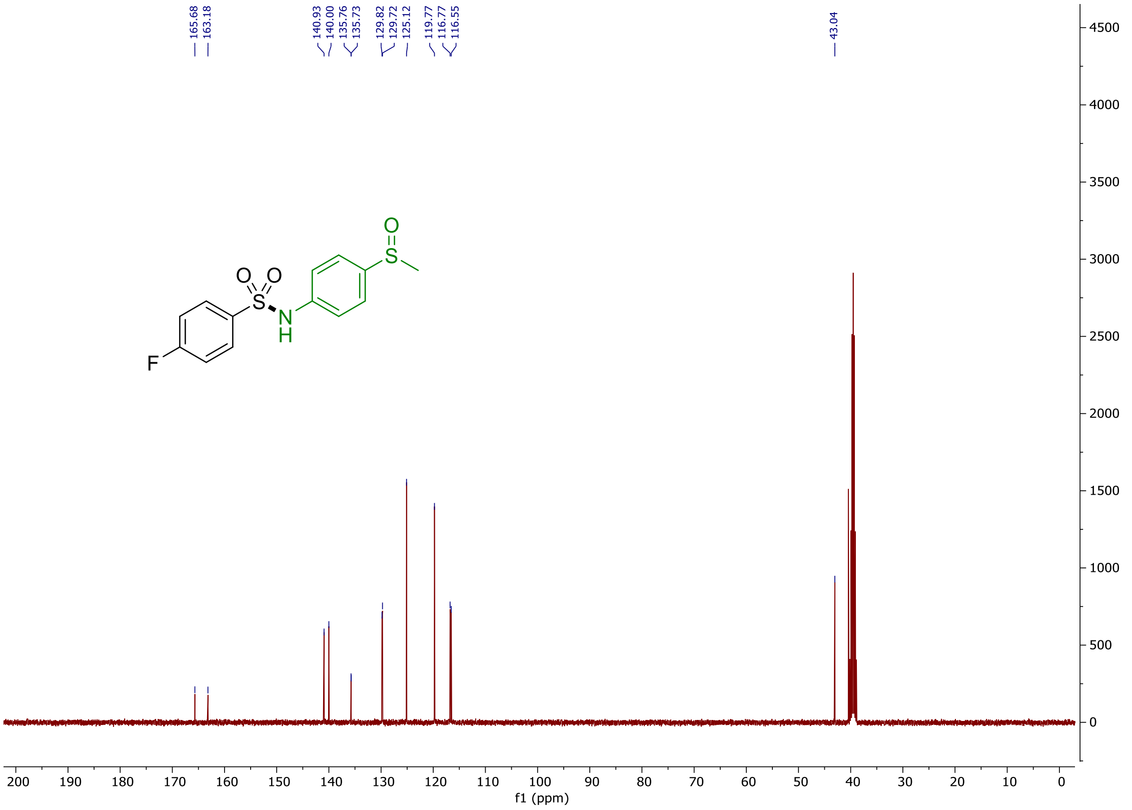

^{13}C NMR of **3i** (101 MHz, DMSO-d_6)

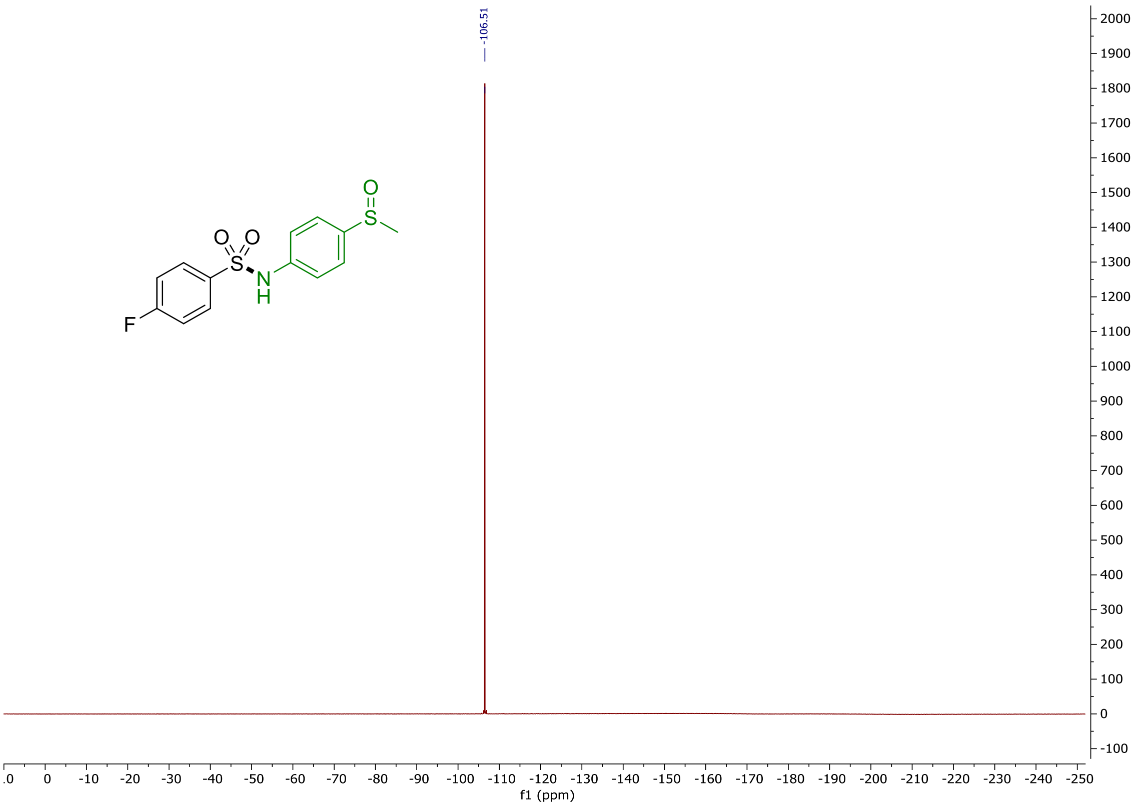

^{19}F NMR of **3i** (376 MHz, DMSO-d_6)

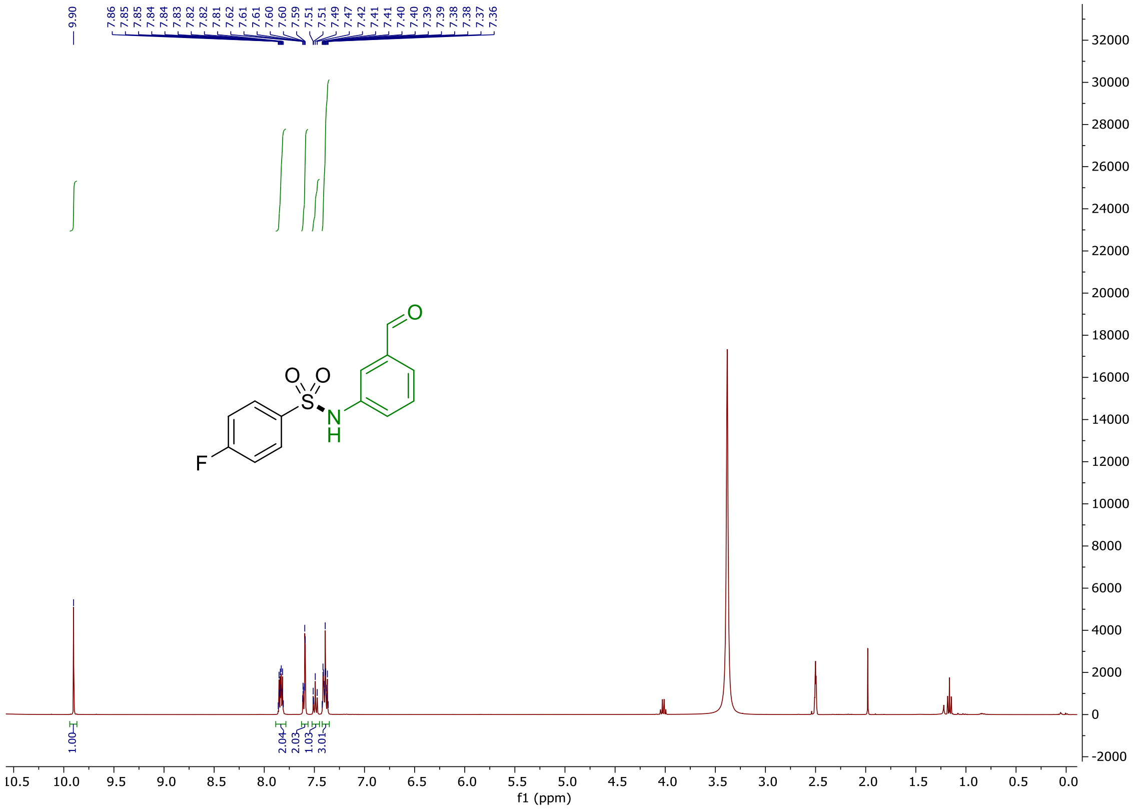

^{1}H NMR of **3j** (400 MHz, DMSO-d_6)

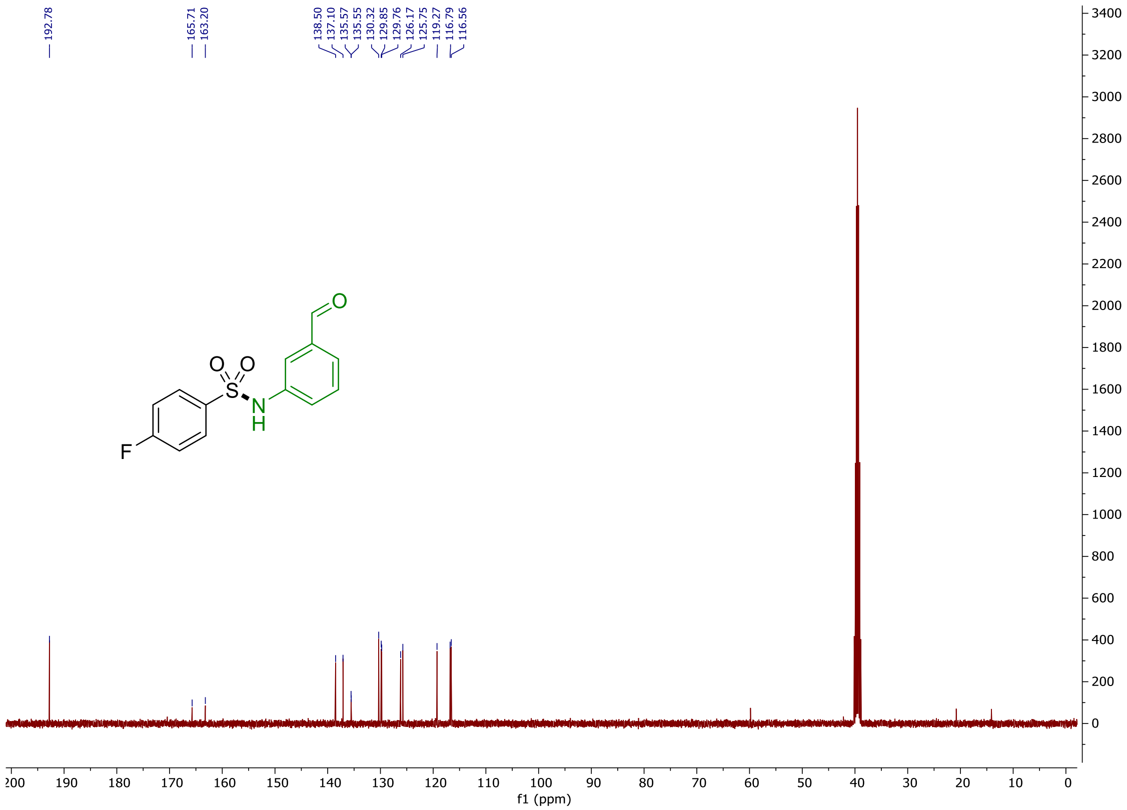

^{13}C NMR of **3j** (101 MHz, DMSO-*d*$_6$)

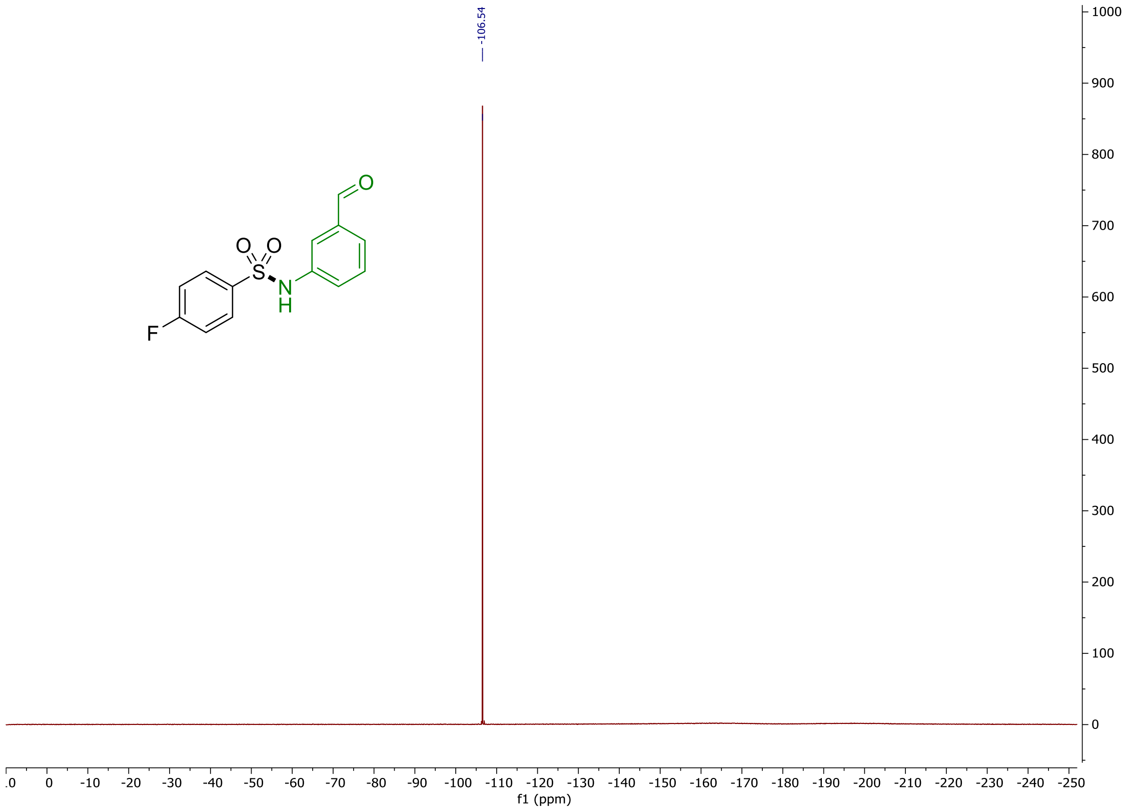

^{19}F NMR of **3j** (376 MHz, DMSO-*d*$_6$)

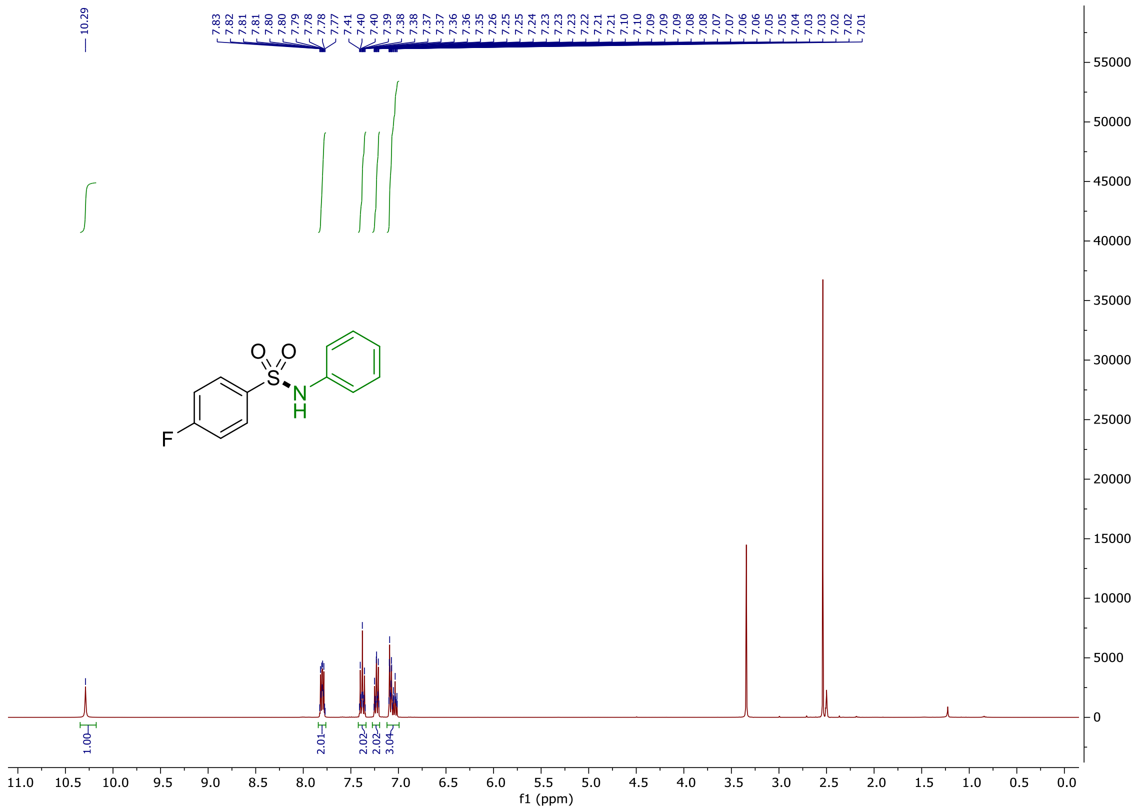

^{1}H NMR of **3k** (400 MHz, DMSO-d_6)

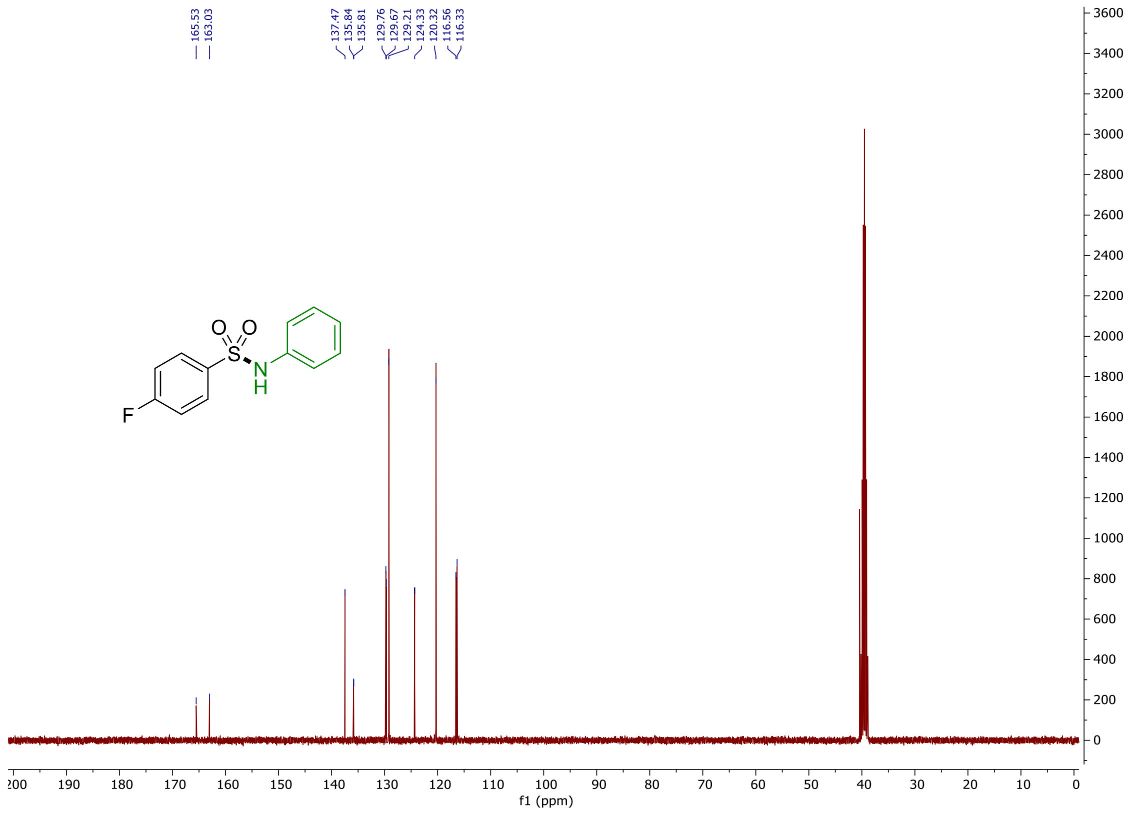

^{13}C NMR of **3k** (101 MHz, DMSO-d_6)

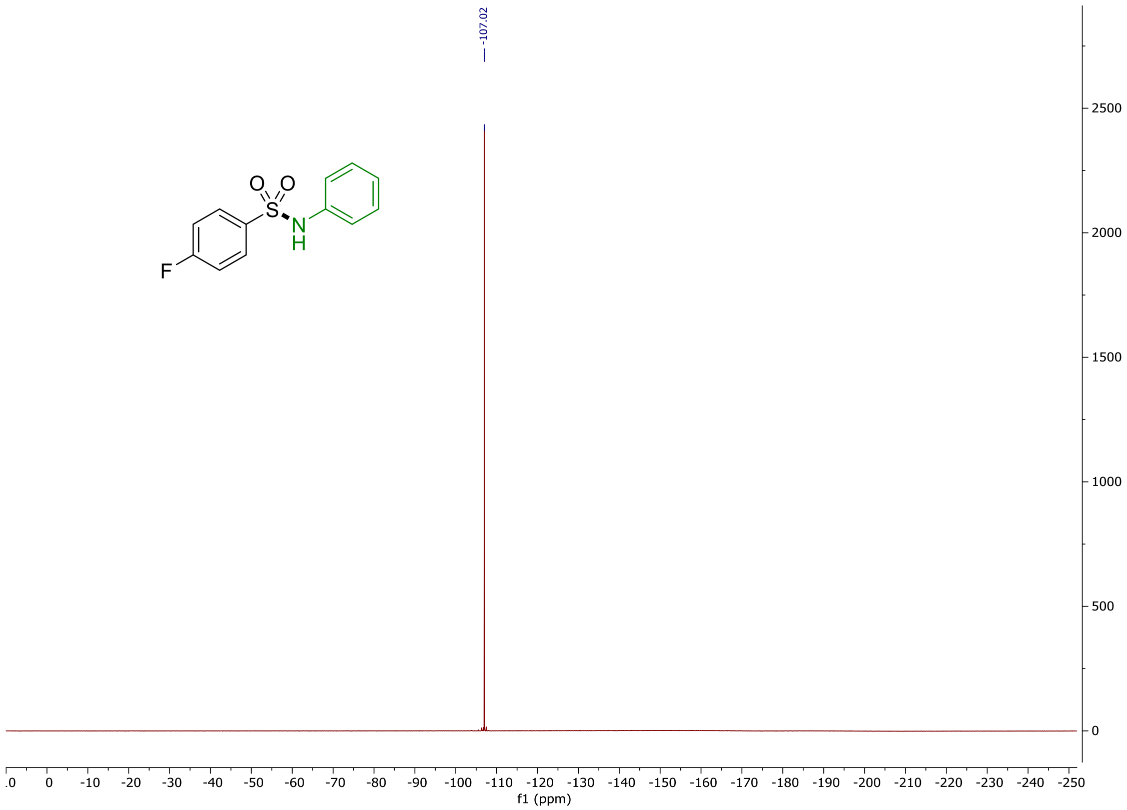

^{19}F NMR of **3k** (376 MHz, DMSO-d_6)

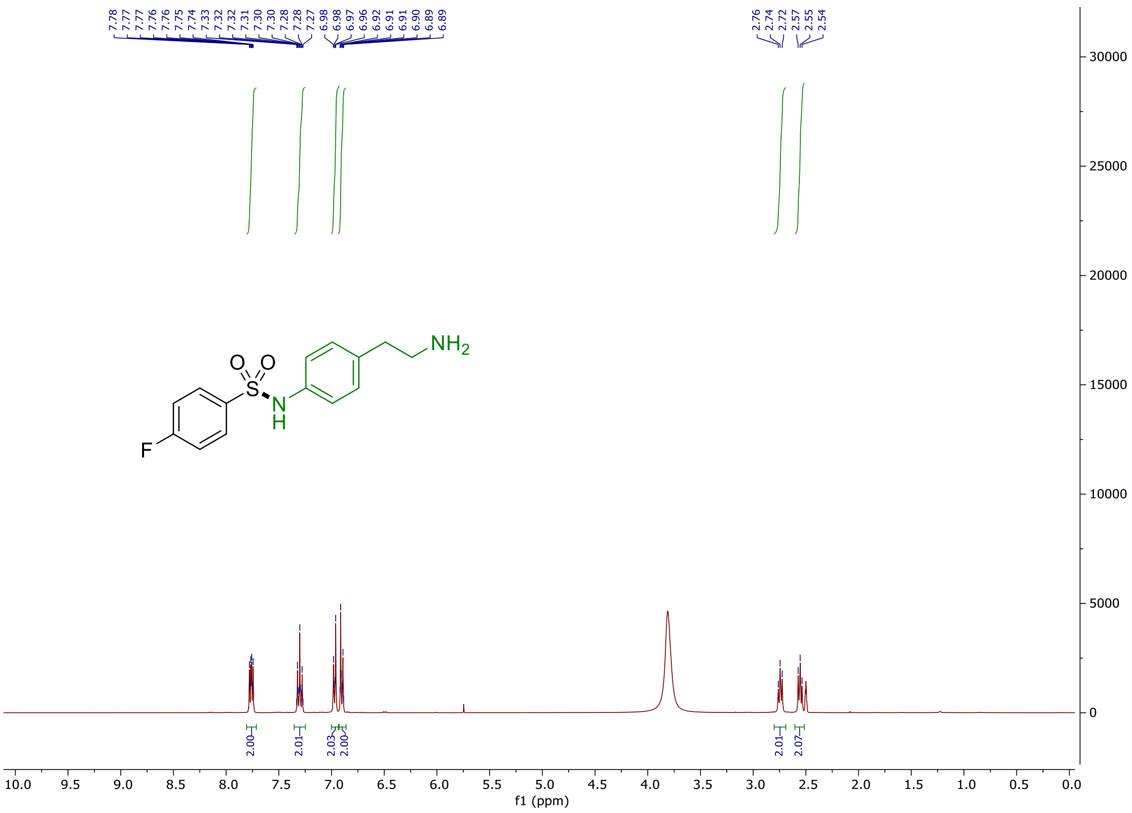

^{1}H NMR of **3l** (400 MHz, DMSO-d_6)

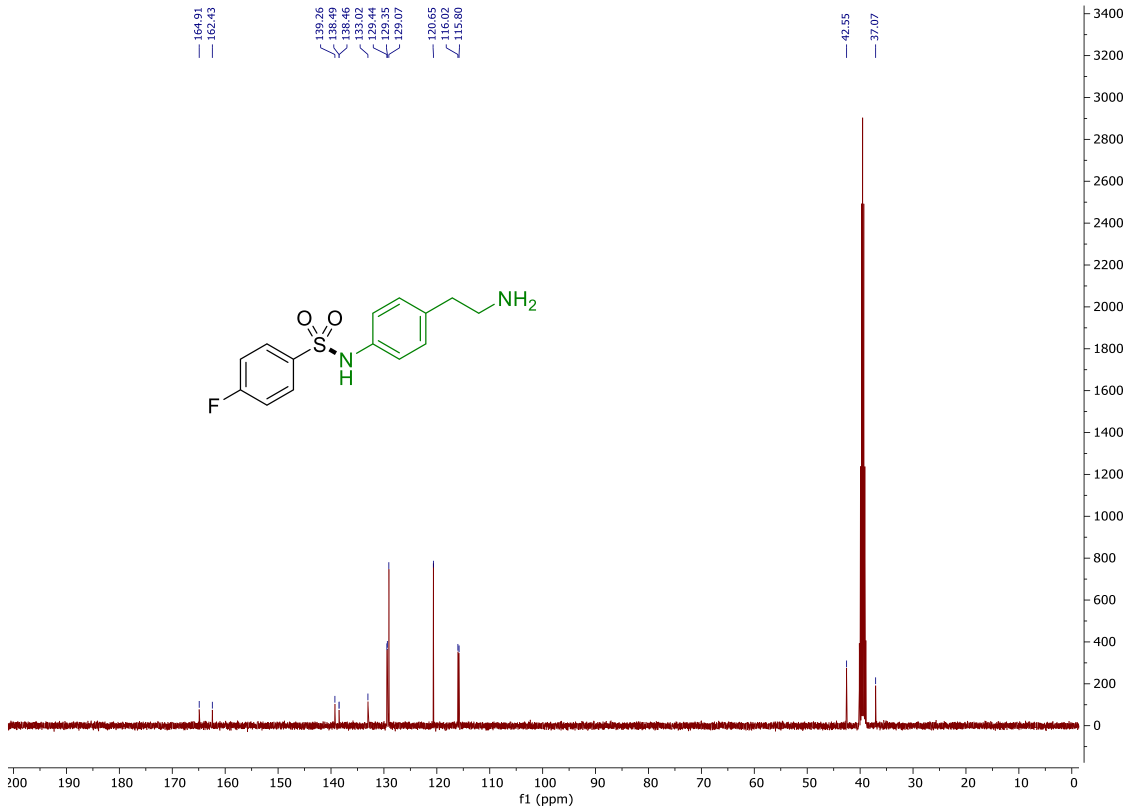

^{13}C NMR of **3l** (101 MHz, DMSO-d_6)

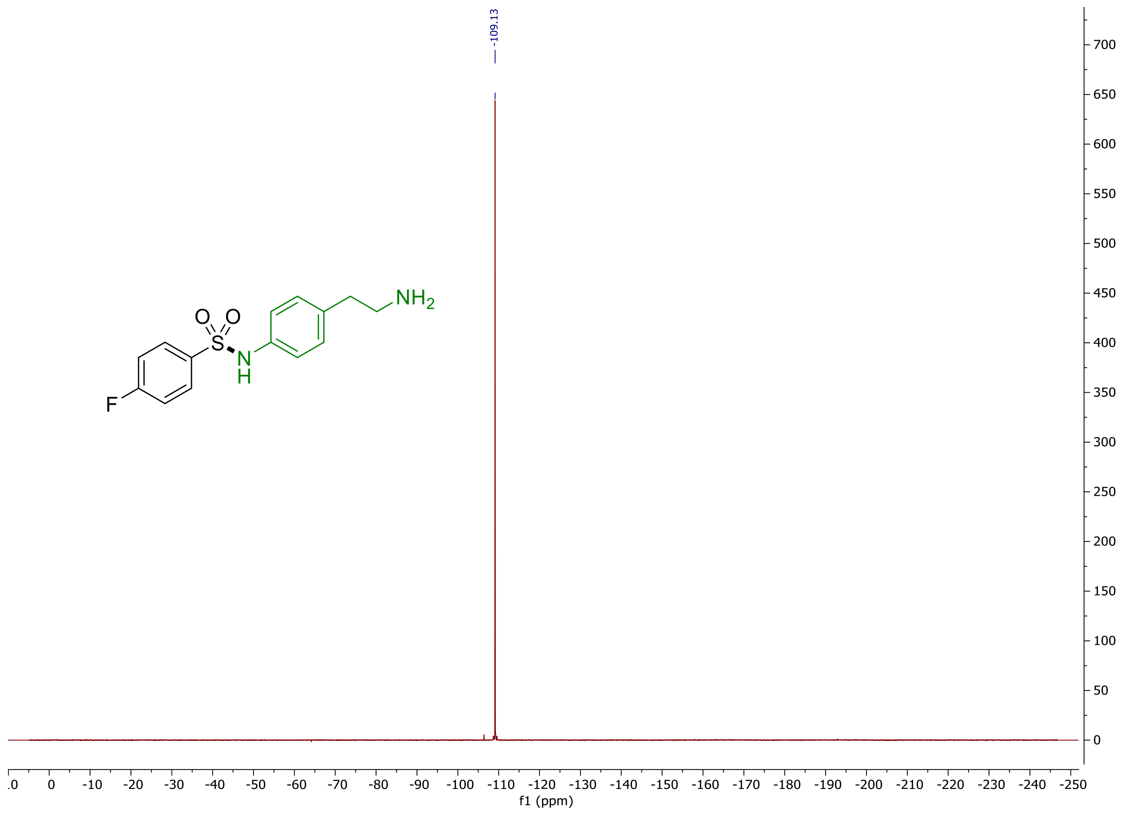

^{19}F NMR of **3l** (376 MHz, DMSO-d_6)

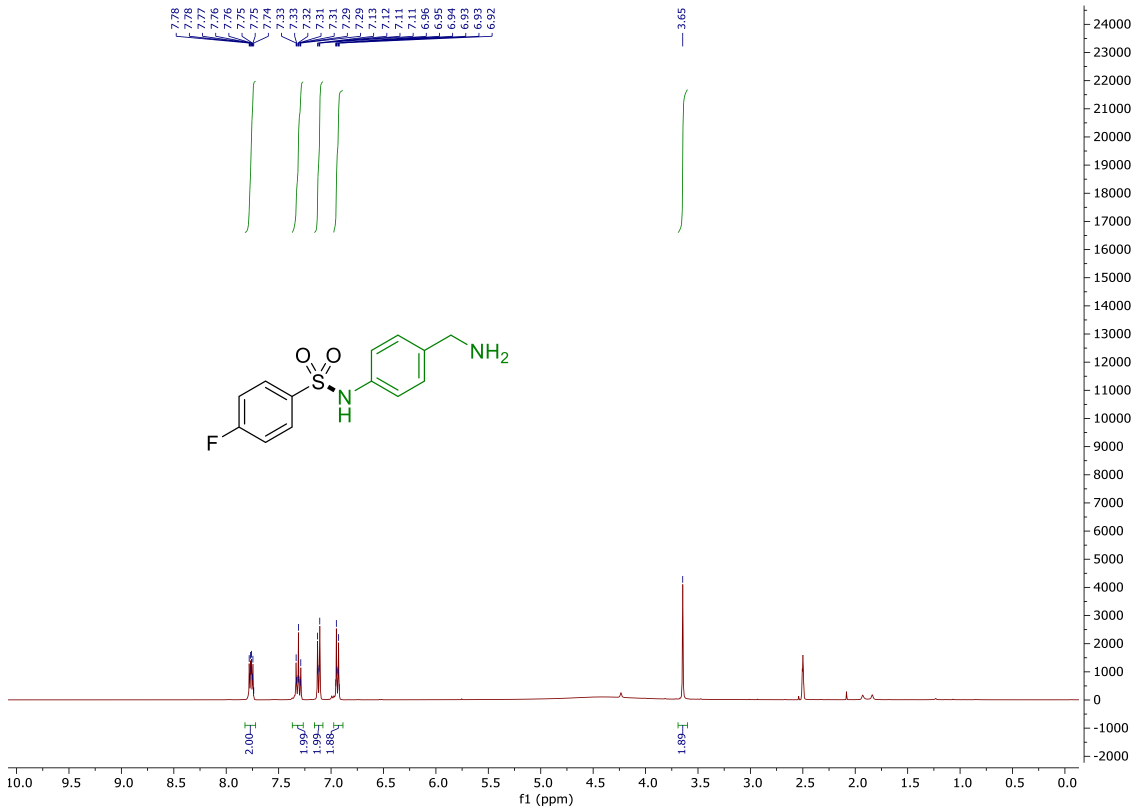

^{1}H NMR of **3m** (400 MHz, DMSO-d_6)

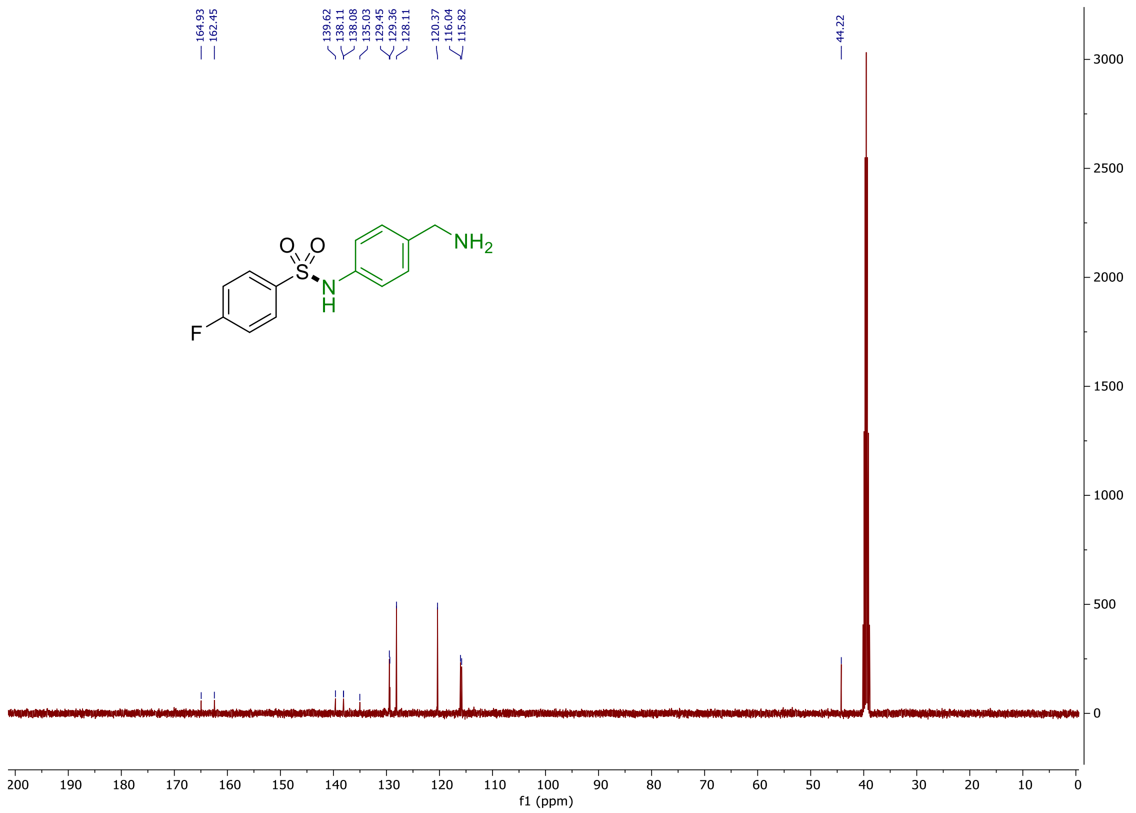

^{13}C NMR of **3m** (101 MHz, DMSO-d_6)

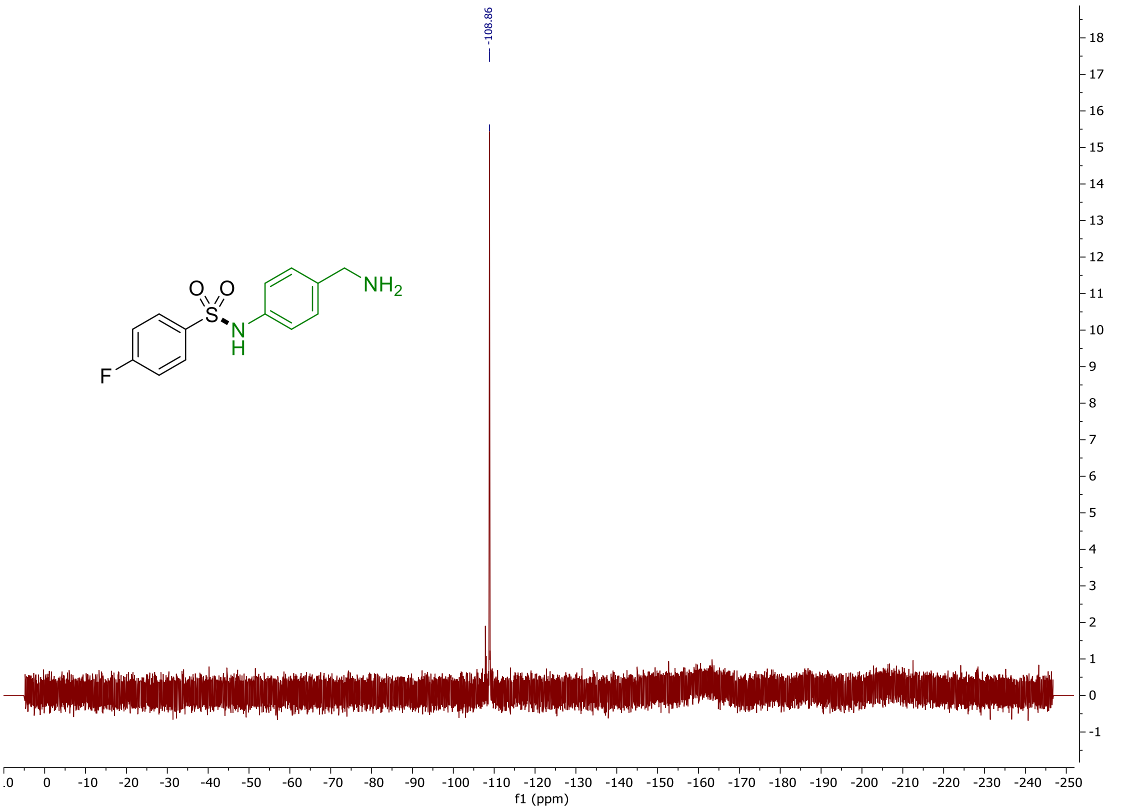

^{19}F NMR of **3m** (376 MHz, DMSO-d_6)

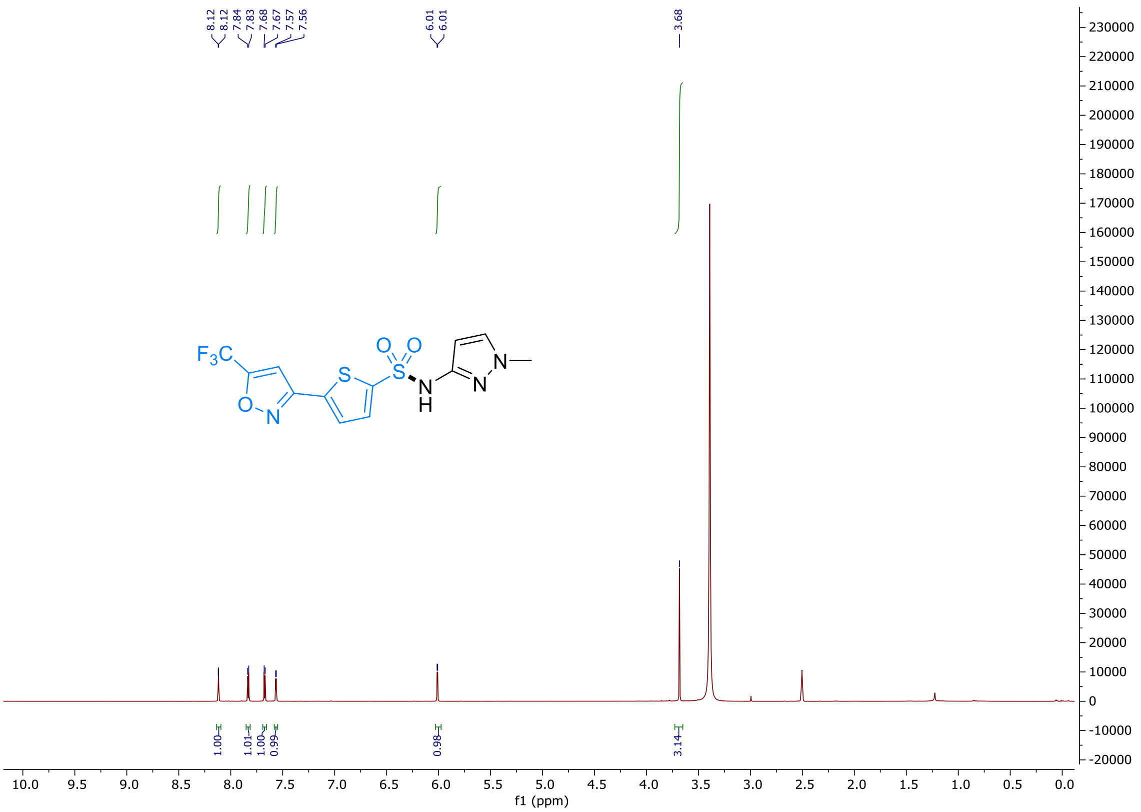

^{1}H NMR of **3n** (400 MHz, DMSO-d_6)

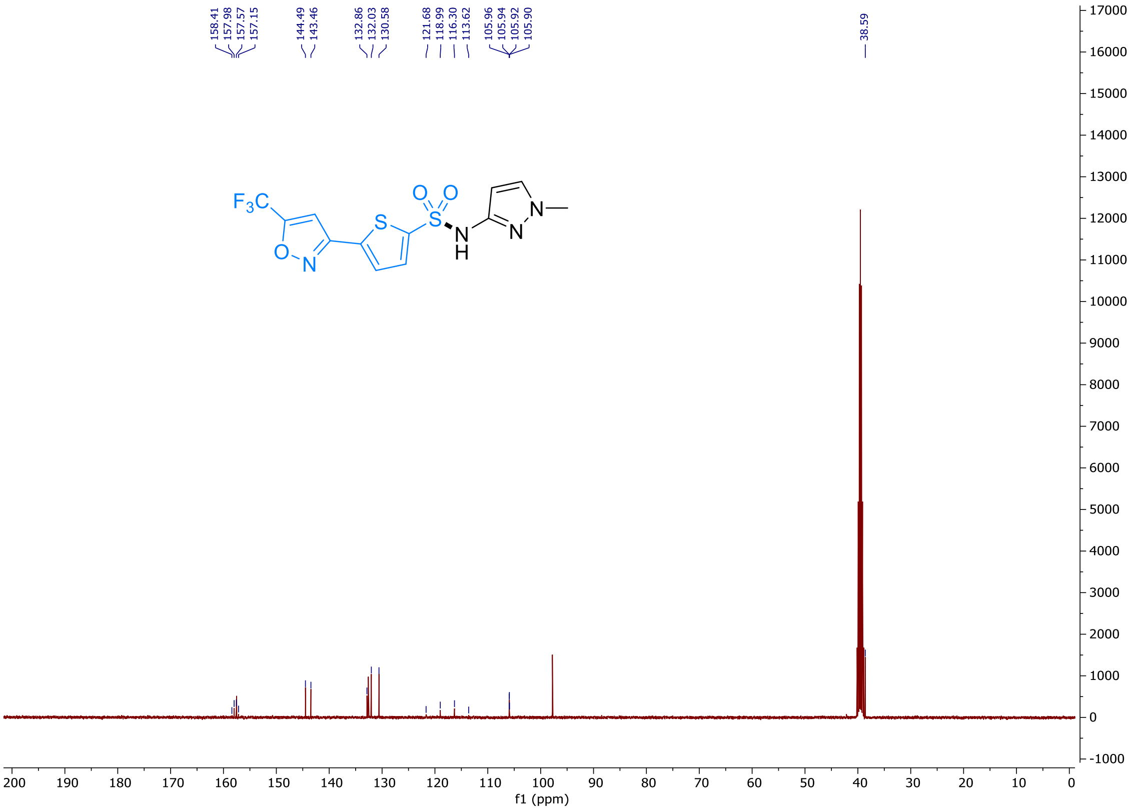

^{13}C NMR of **3n** (101 MHz, DMSO-d_6)

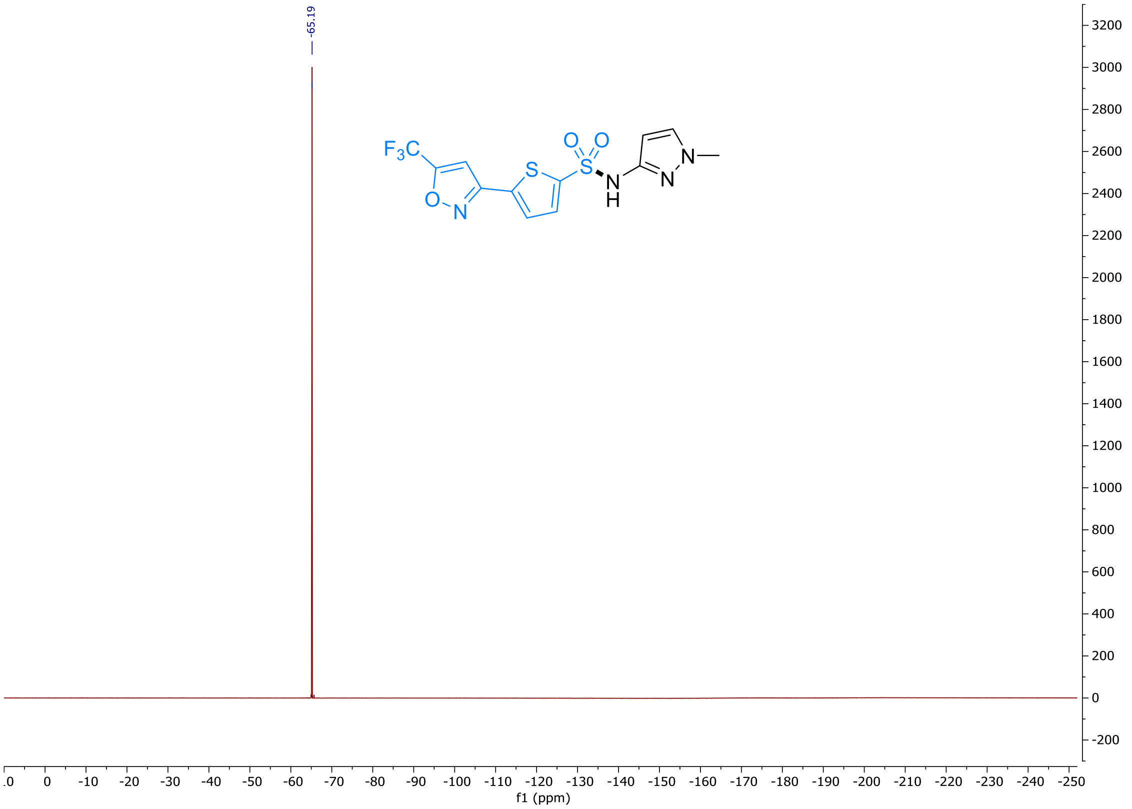

^{19}F NMR of **3n** (376 MHz, DMSO-d_6)

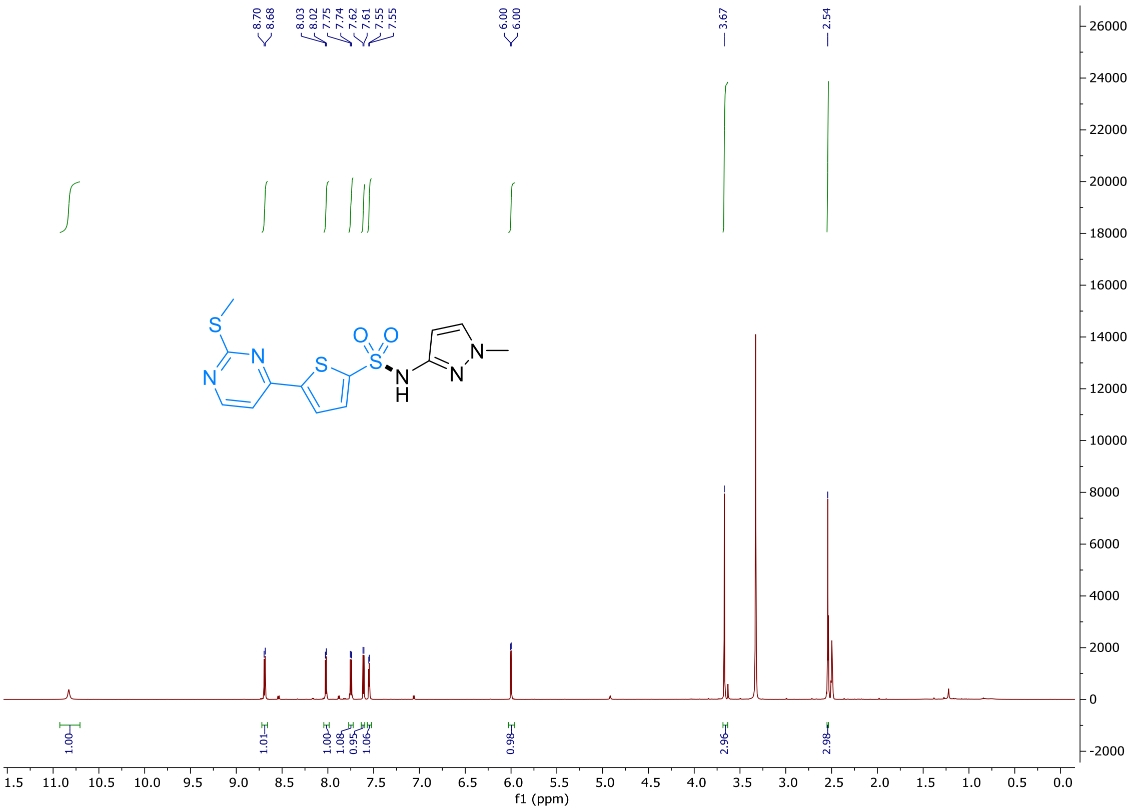

^{1}H NMR of **3o** (400 MHz, DMSO-d_6)

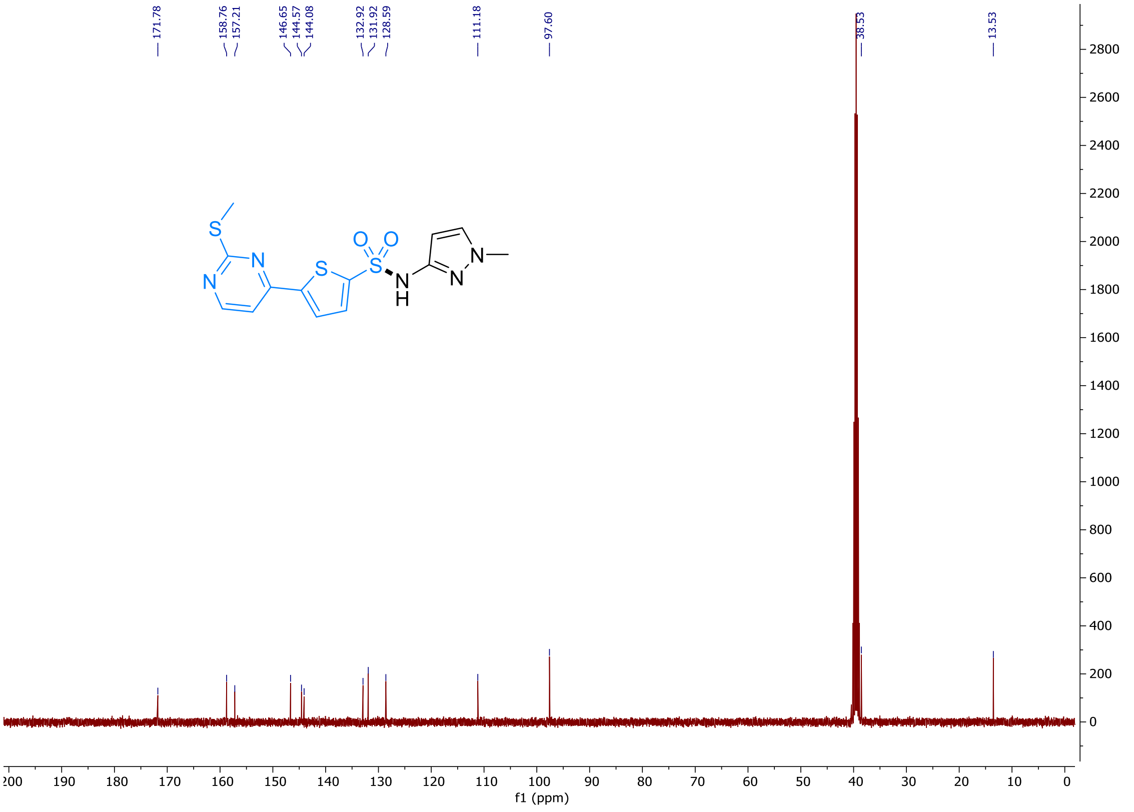

^{13}C NMR of **3o** (101 MHz, DMSO-d_6)

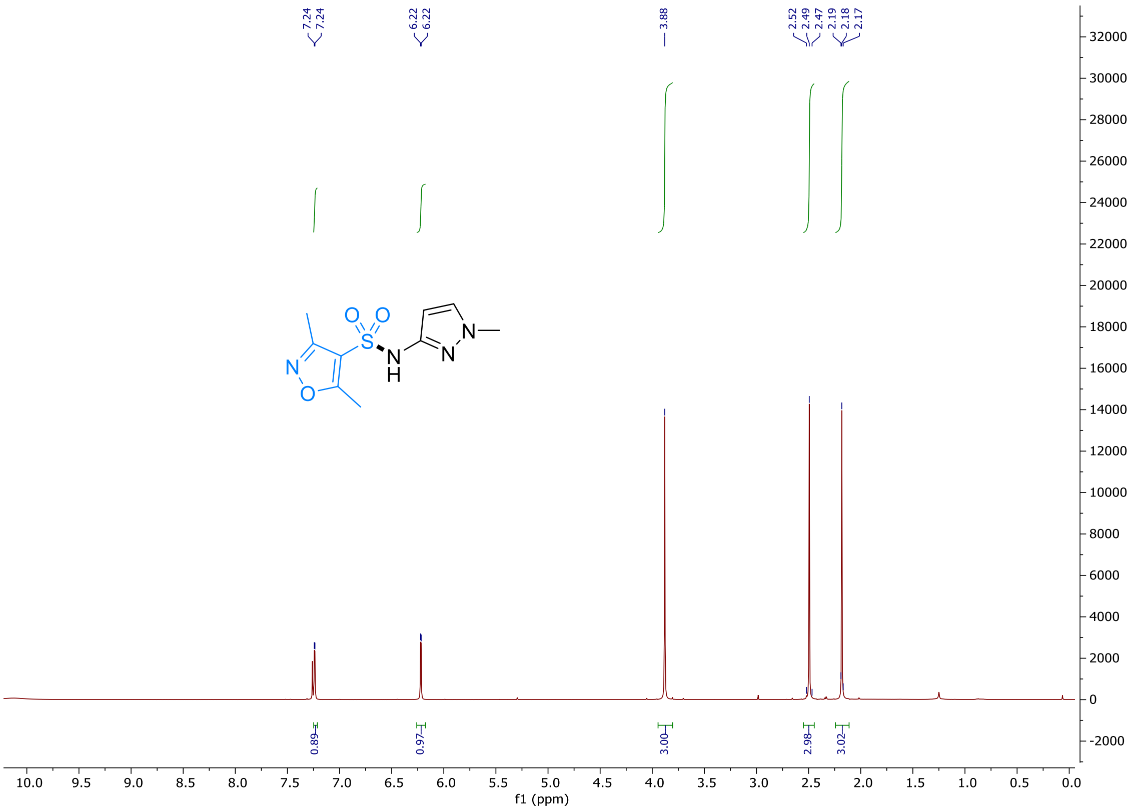

^{1}H NMR of **3p** (400 MHz, $CDCl_3$)

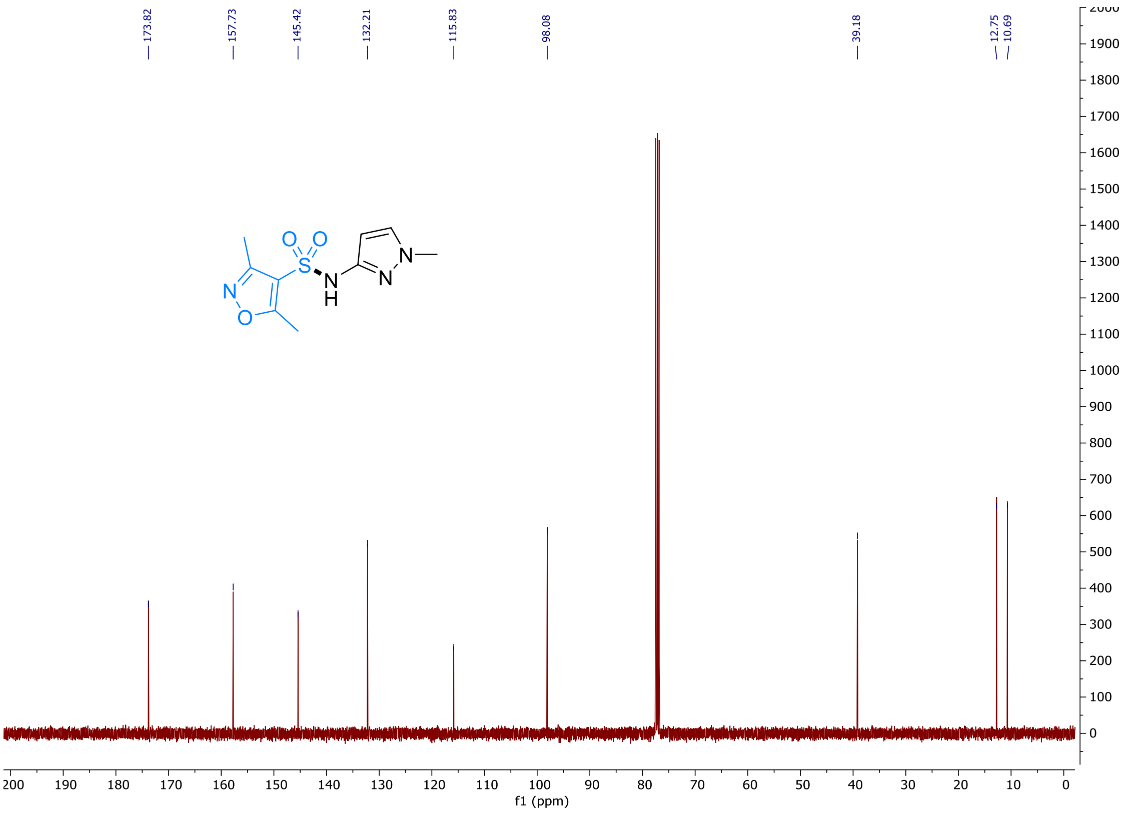

^{13}C NMR of **3p** (101 MHz, $CDCl_3$)

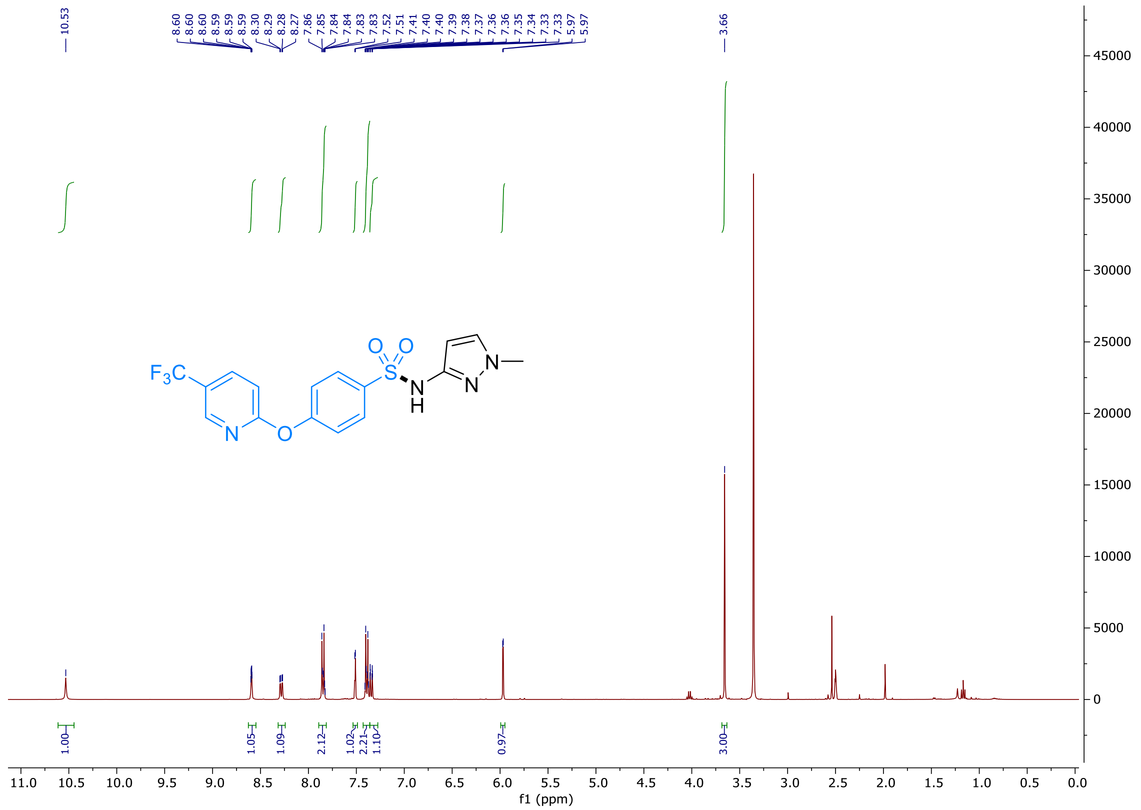

^{1}H NMR of **3q** (400 MHz, DMSO-d_6)

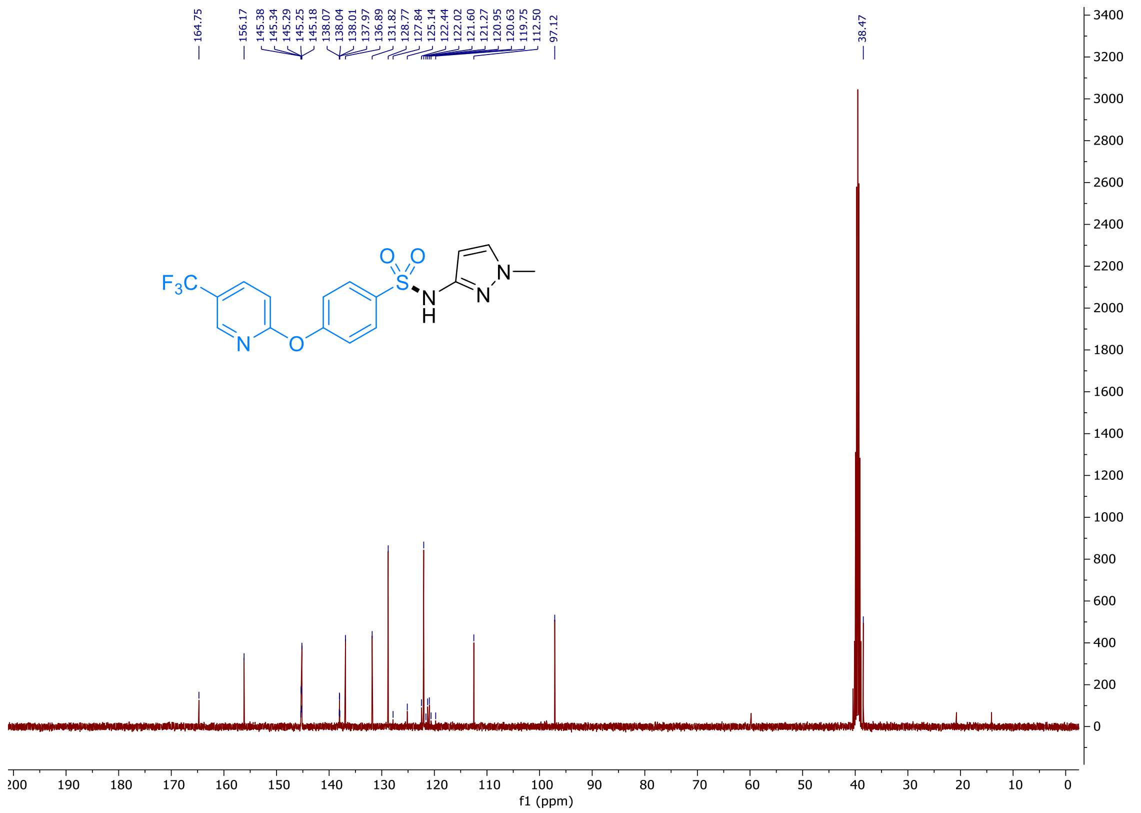

^{13}C NMR of **3q** (101 MHz, DMSO-d_6)

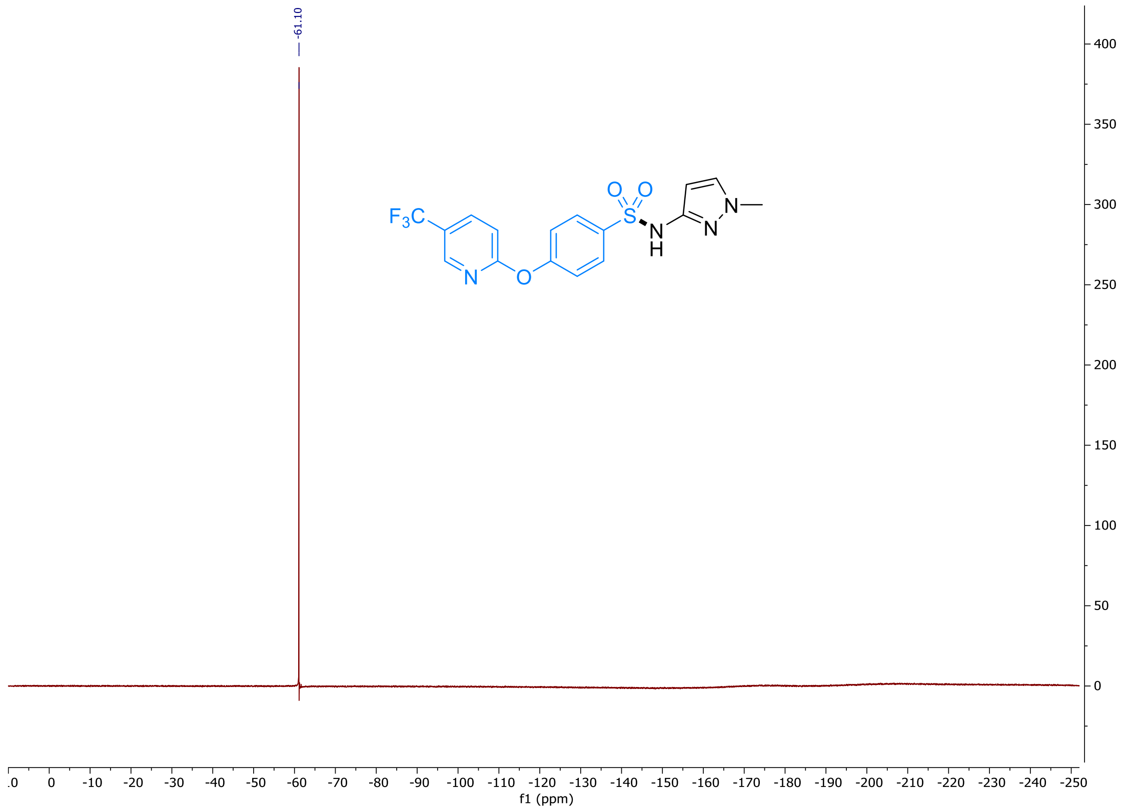

^{19}F NMR of **3q** (376 MHz, DMSO-d_6)

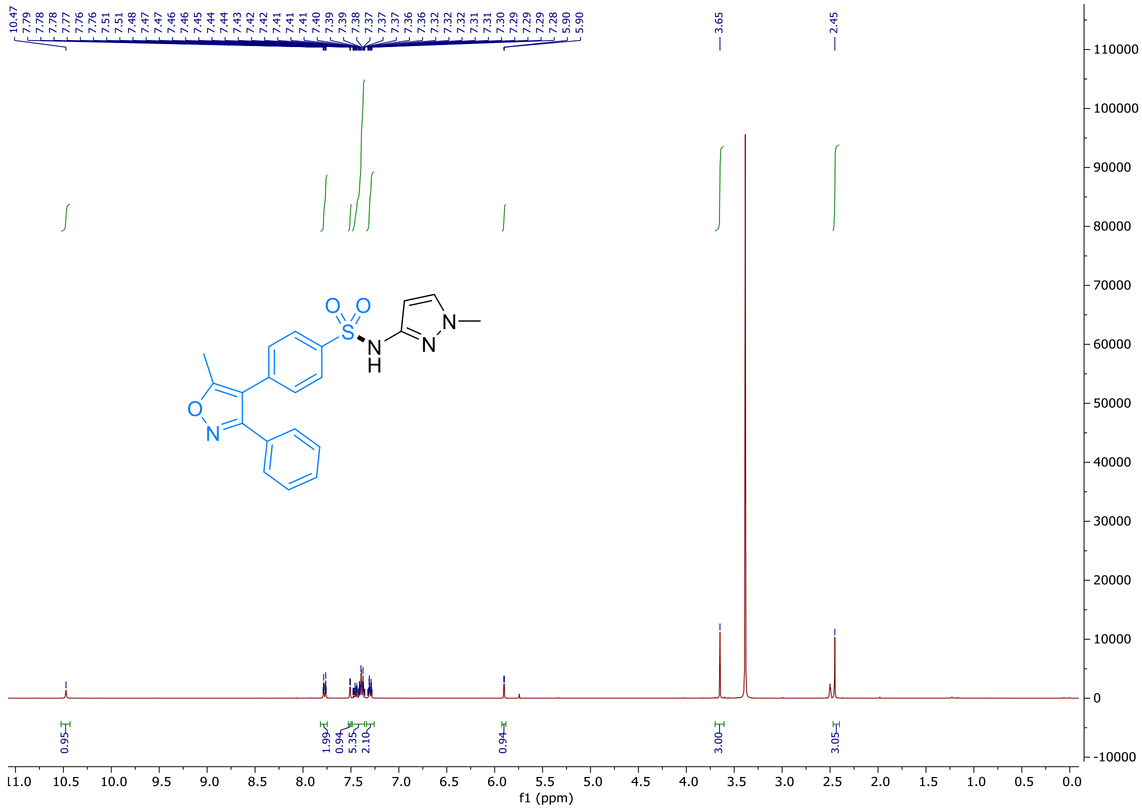

^{1}H NMR of **3r** (400 MHz, DMSO-d_6)

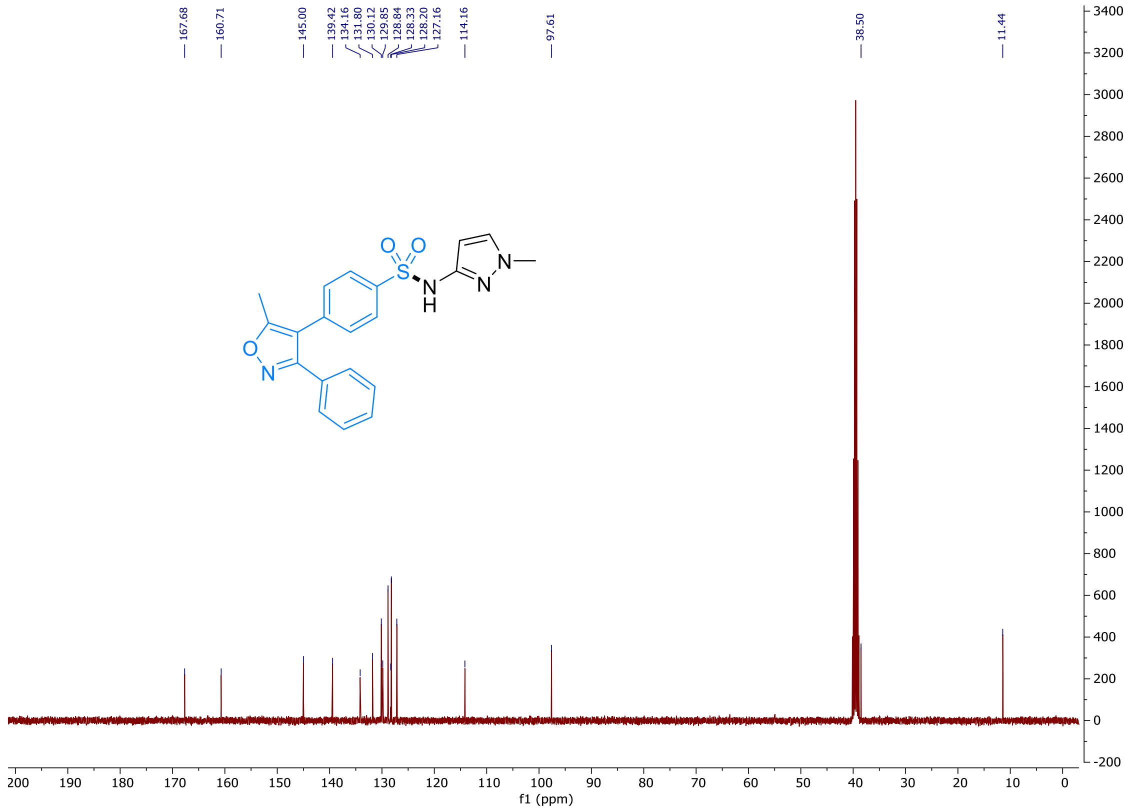

^{13}C NMR of **3r** (101 MHz, DMSO-d_6)

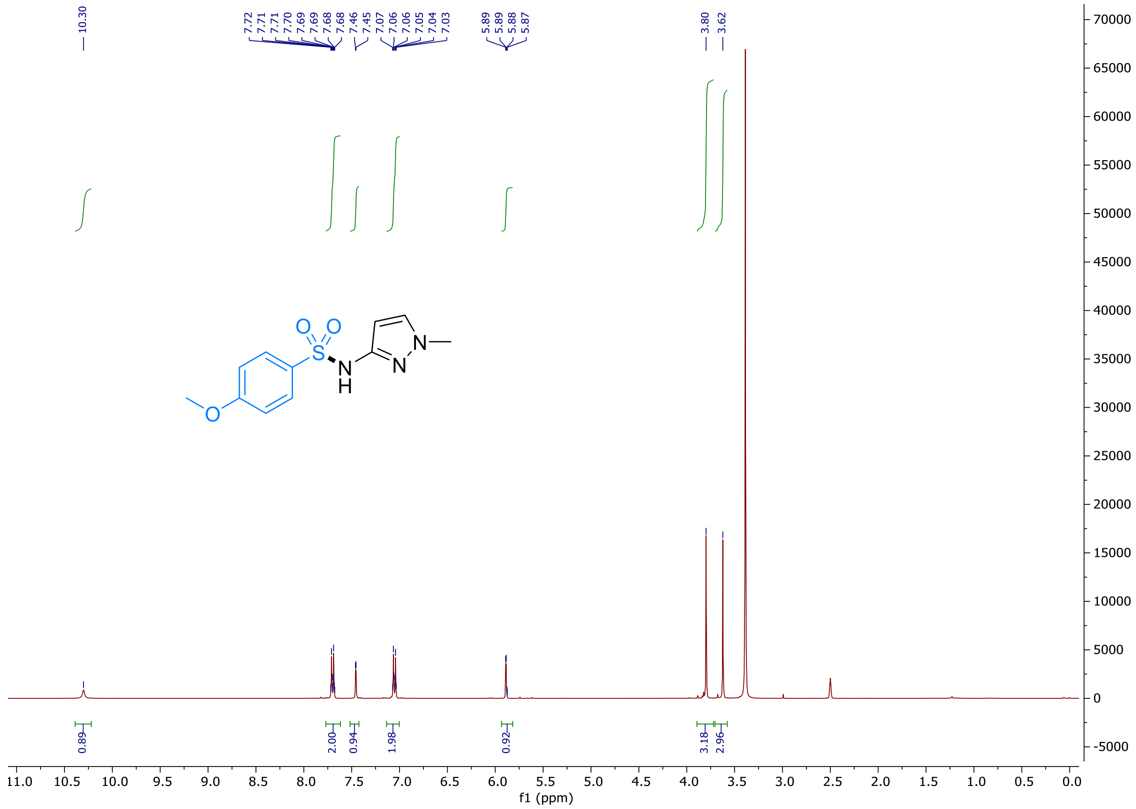

^{1}H NMR of **3s** (400 MHz, DMSO-d_6)

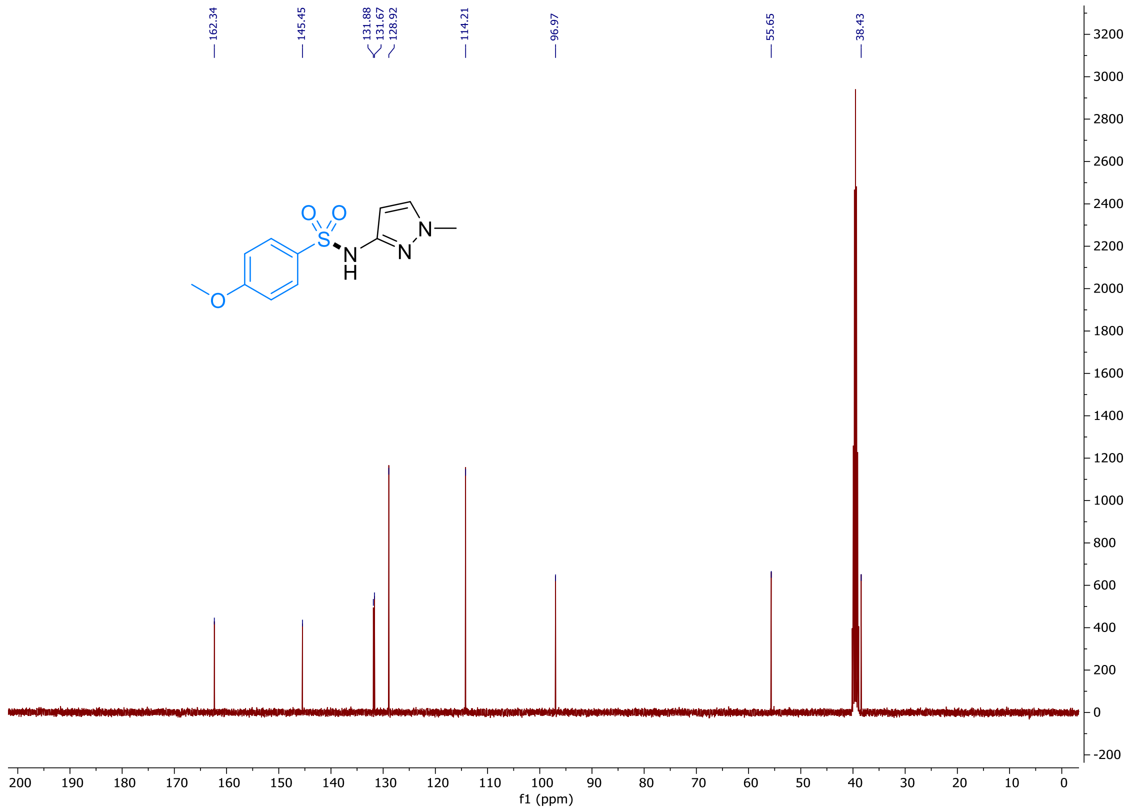

^{13}C NMR of **3s** (101 MHz, DMSO-d_6)

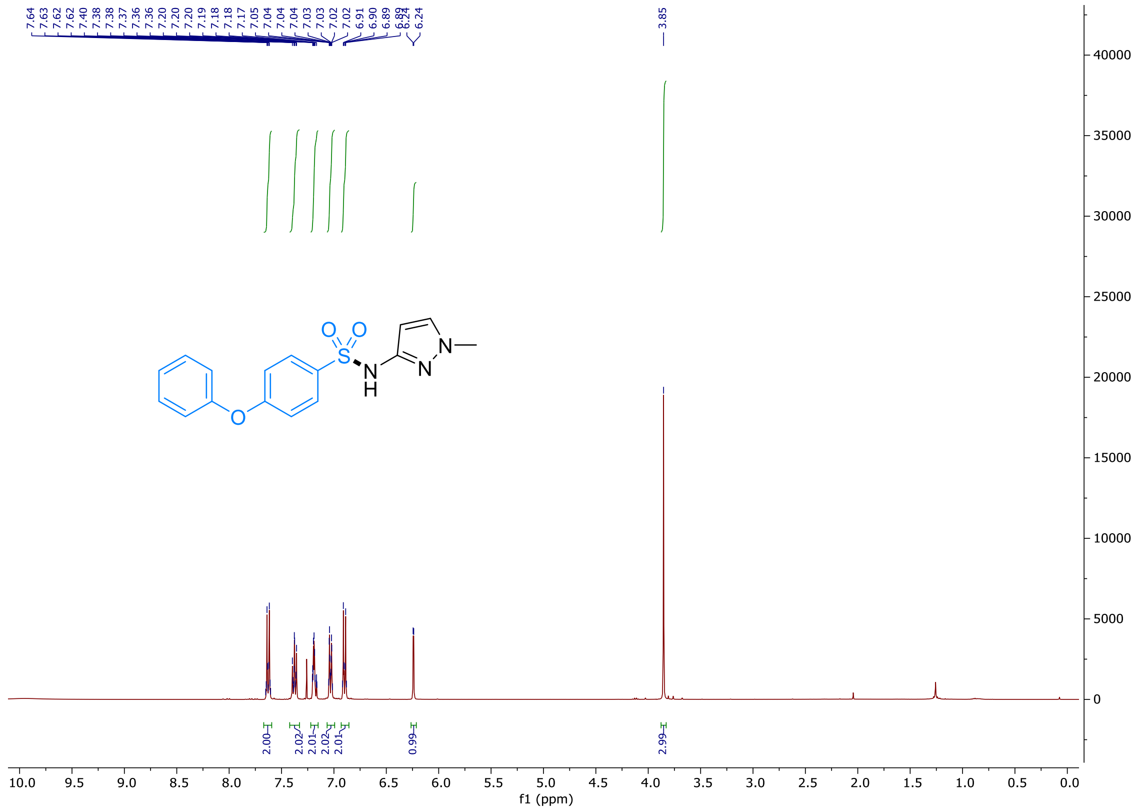

^{1}H NMR of **3t** (400 MHz, $CDCl_3$)

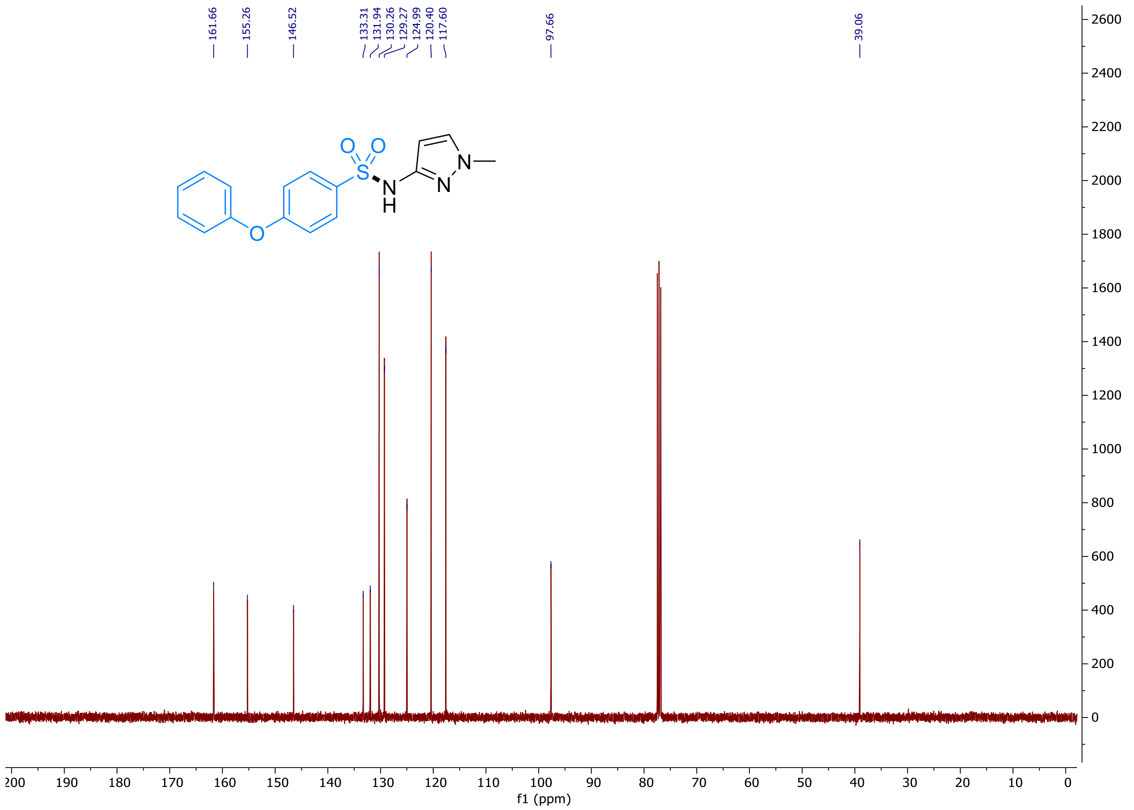

^{13}C NMR of **3t** (101 MHz, $CDCl_3$)

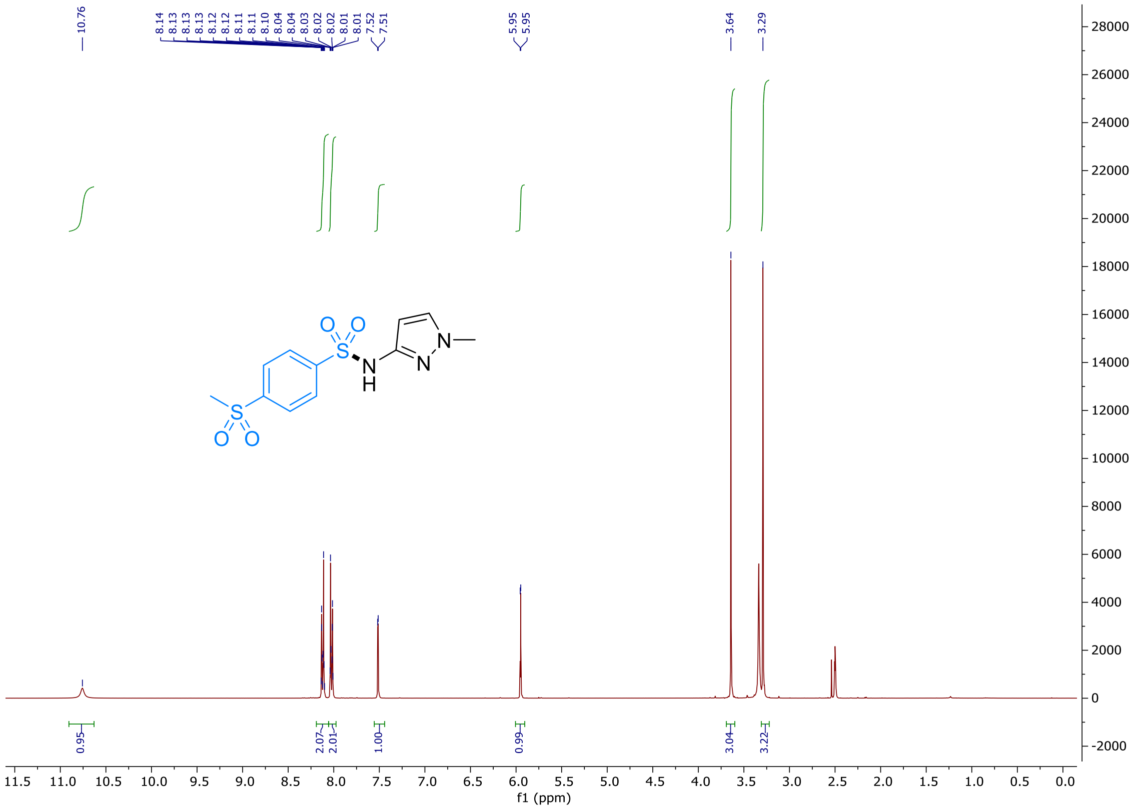

^{1}H NMR of **3u** (400 MHz, DMSO-d_6)

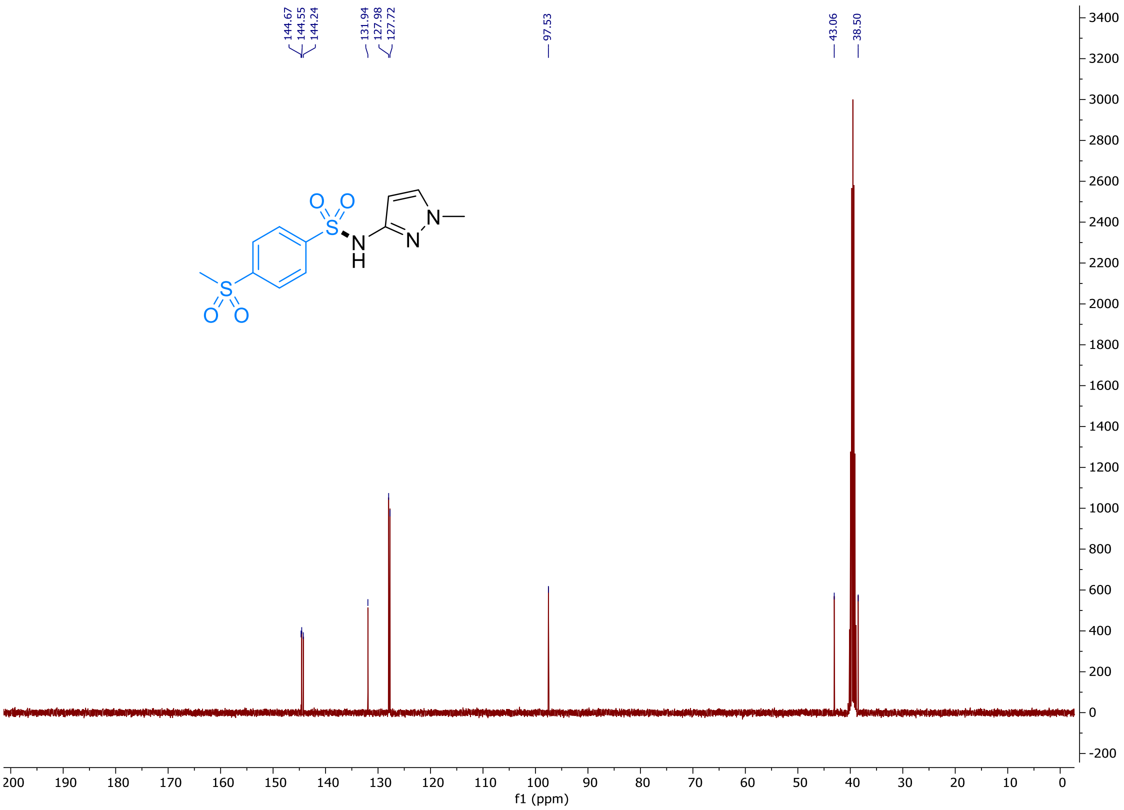

^{13}C NMR of **3u** (101 MHz, DMSO-d_6)

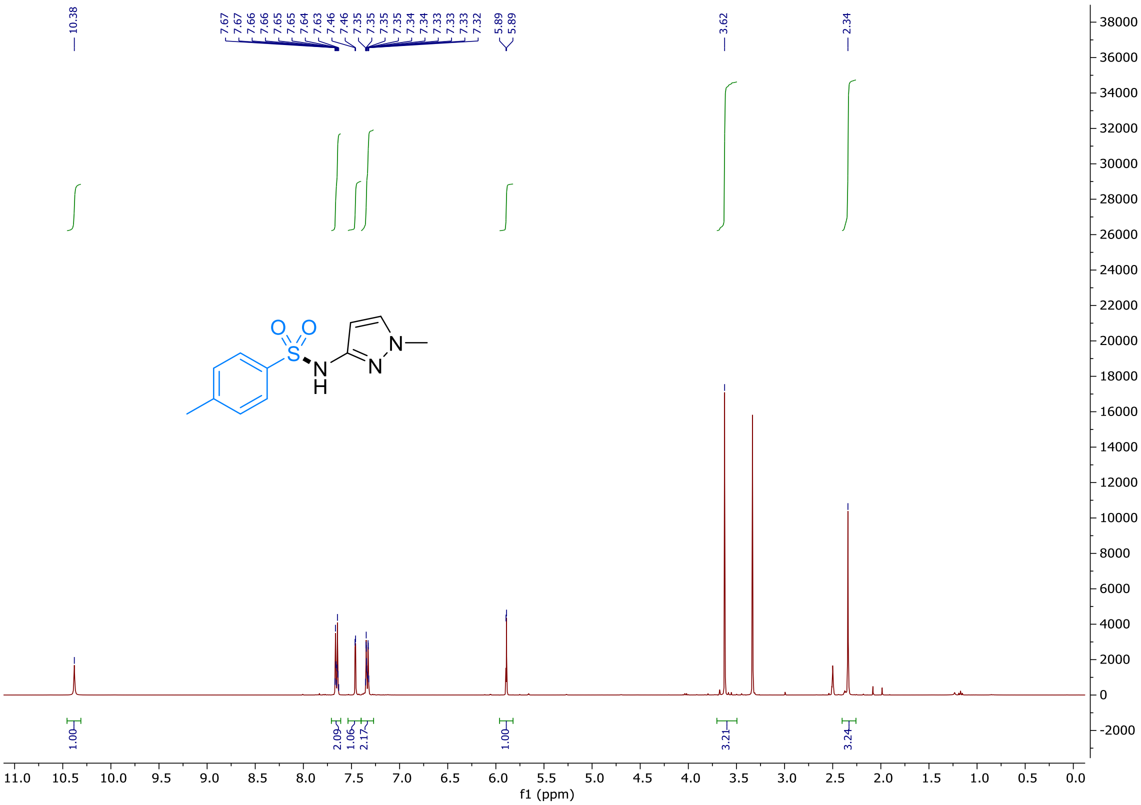

^{1}H NMR of **3v** (400 MHz, DMSO-d_6)

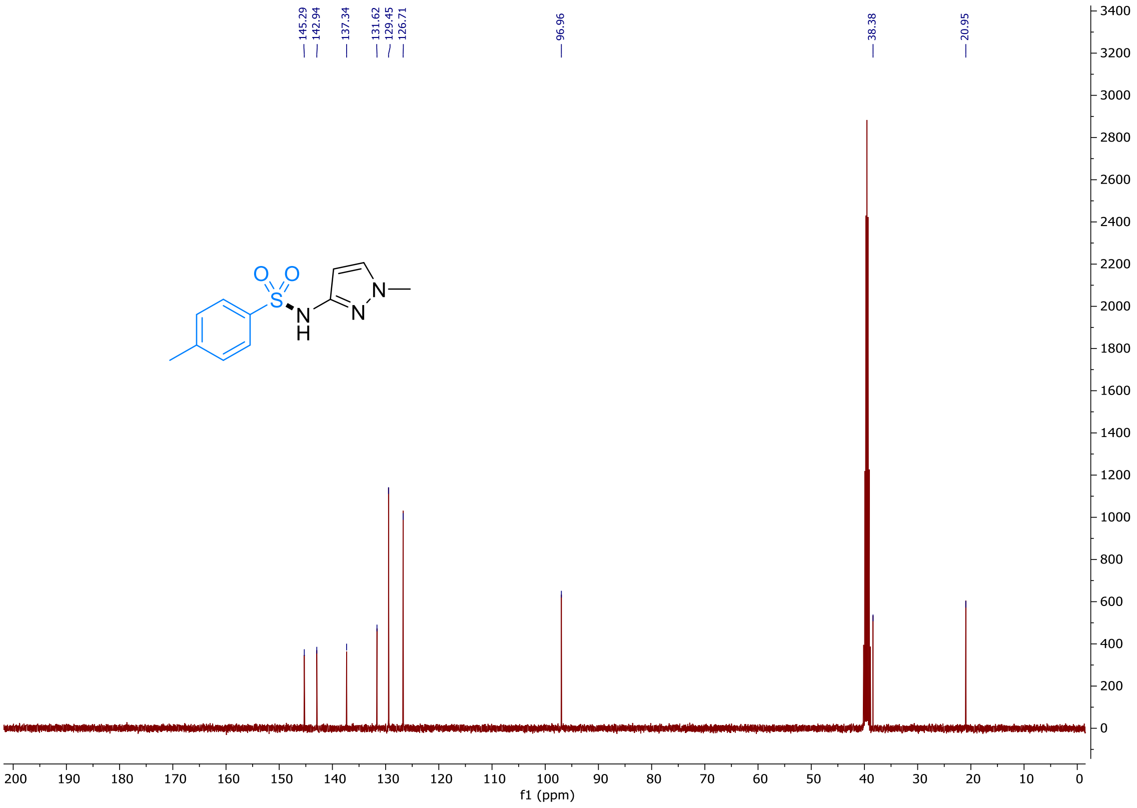

^{13}C NMR of **3v** (101 MHz, DMSO-d_6)

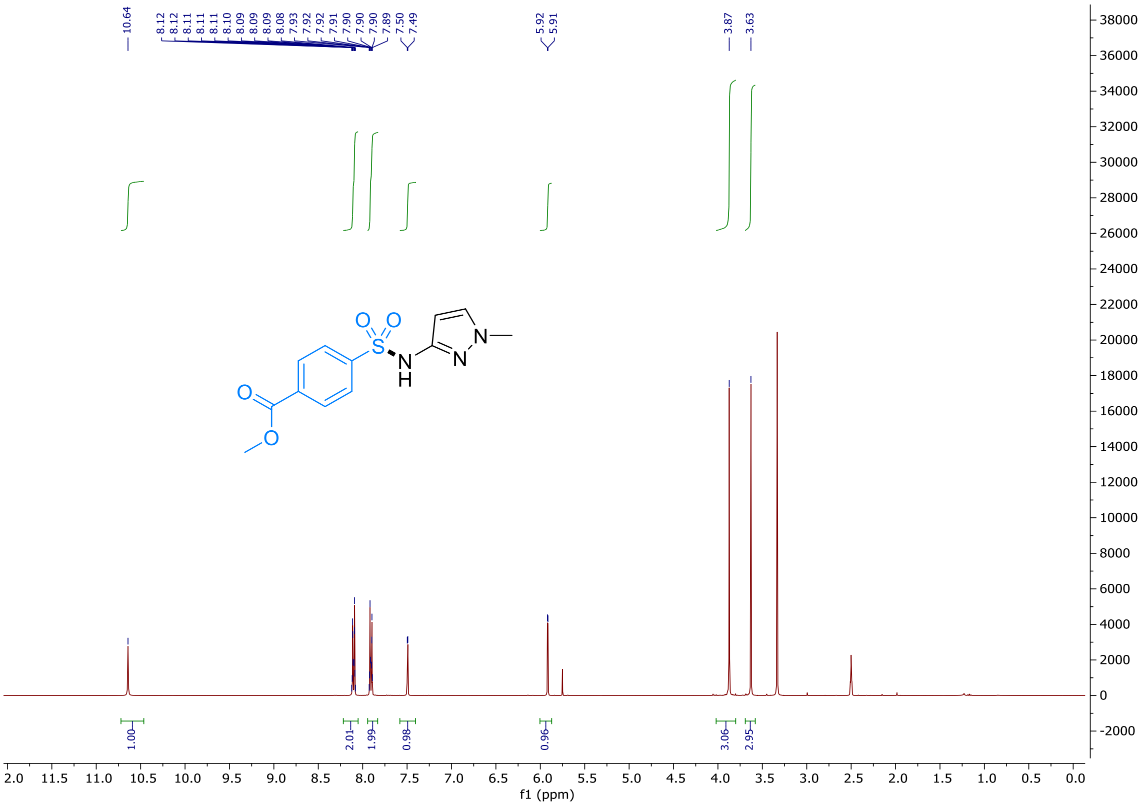

^{1}H NMR of **3w** (400 MHz, DMSO-d_6)

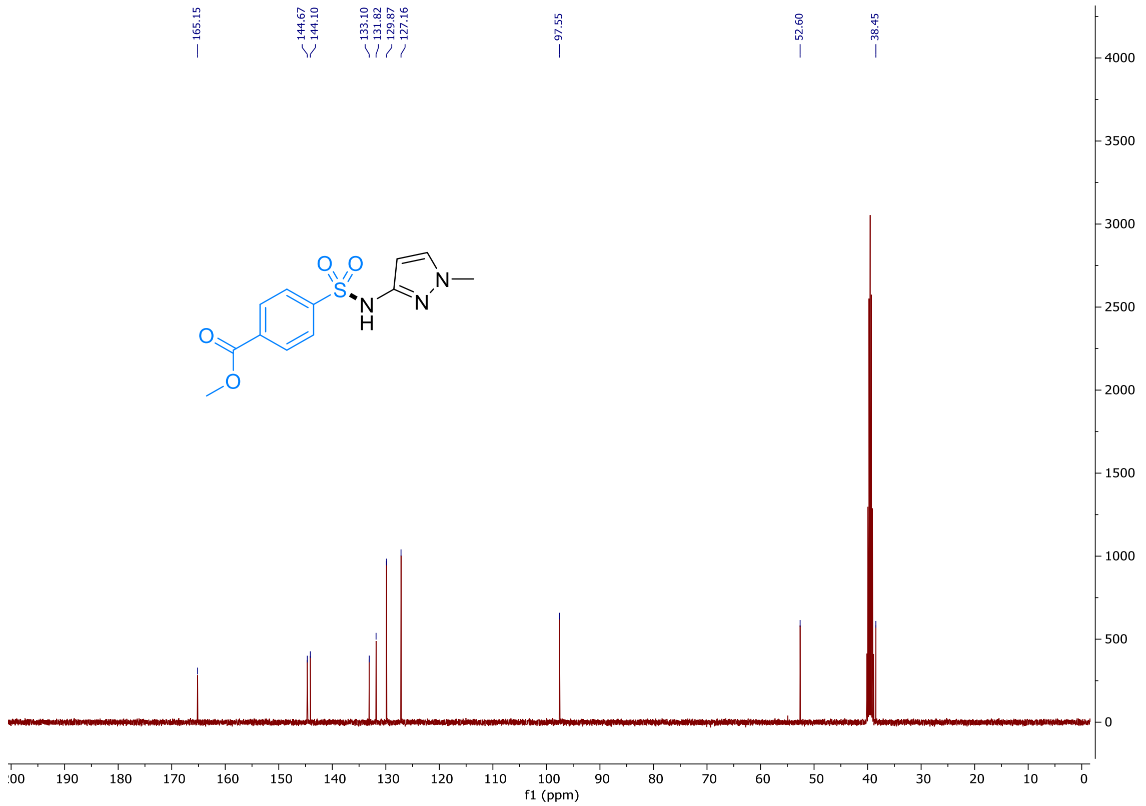

^{13}C NMR of **3w** (101 MHz, DMSO-d_6)

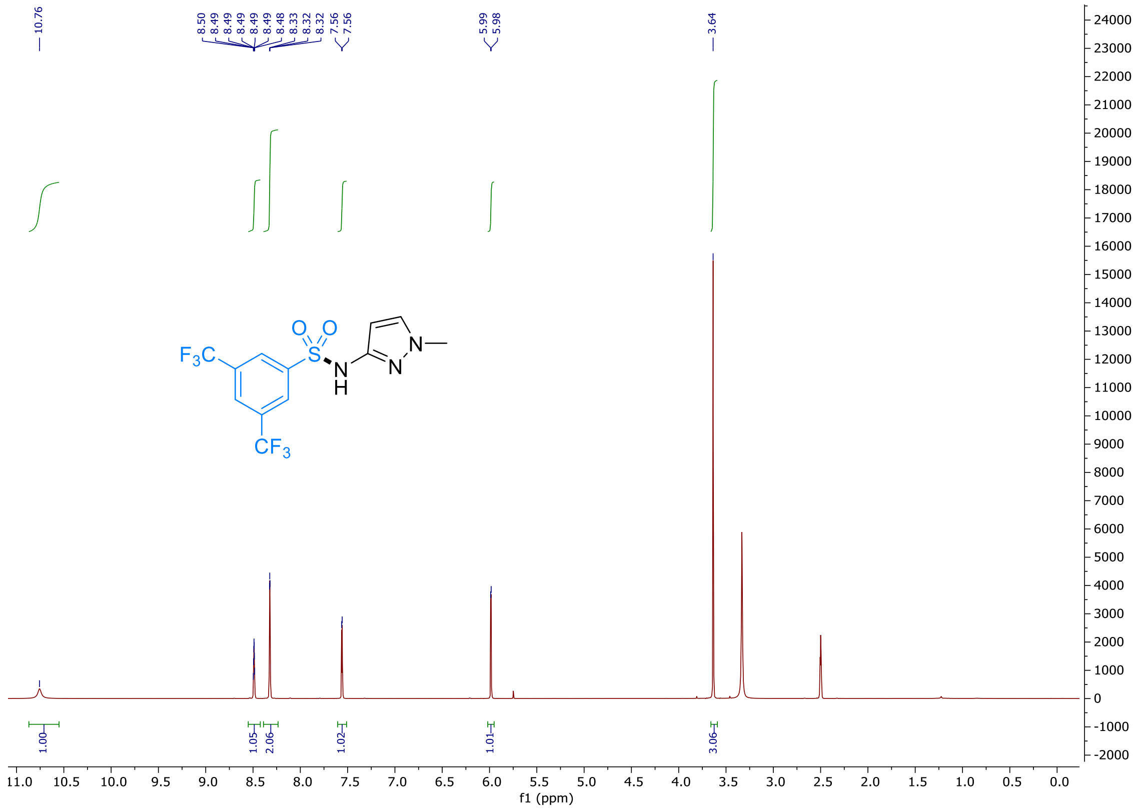

^{1}H NMR of **3x** (400 MHz, DMSO-d_6)

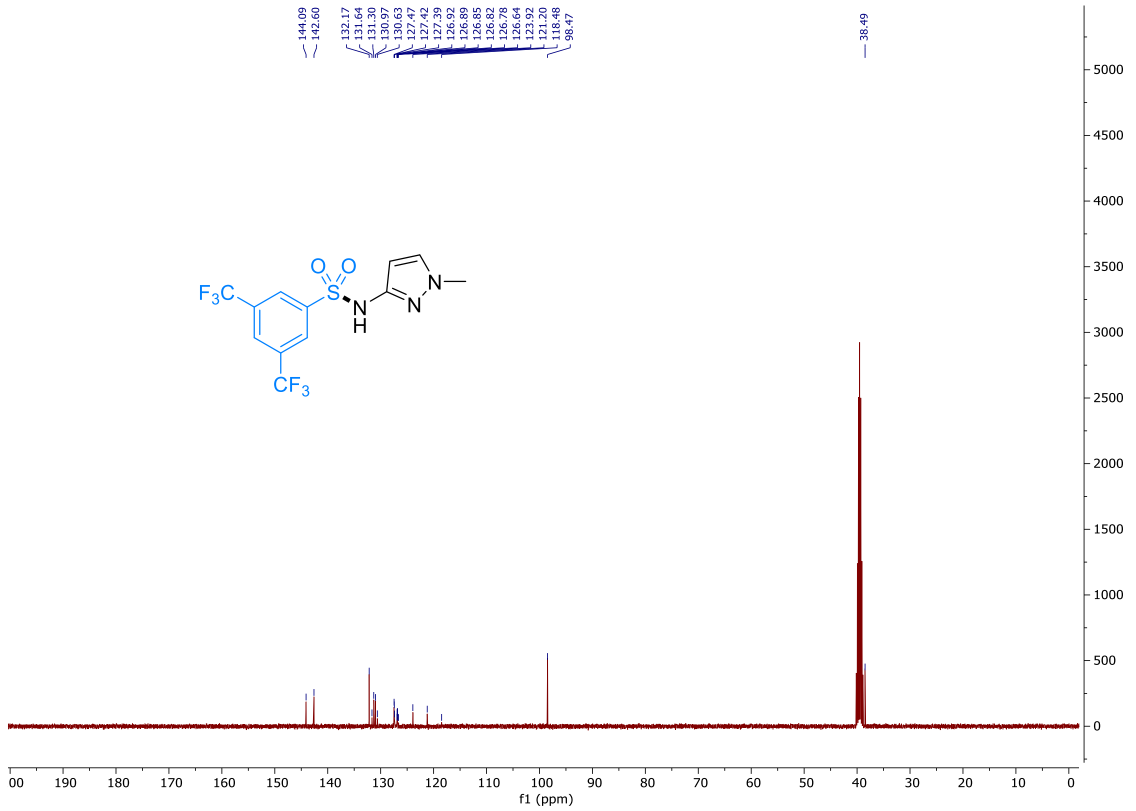

^{13}C NMR of **3x** (101 MHz, DMSO-d_6)

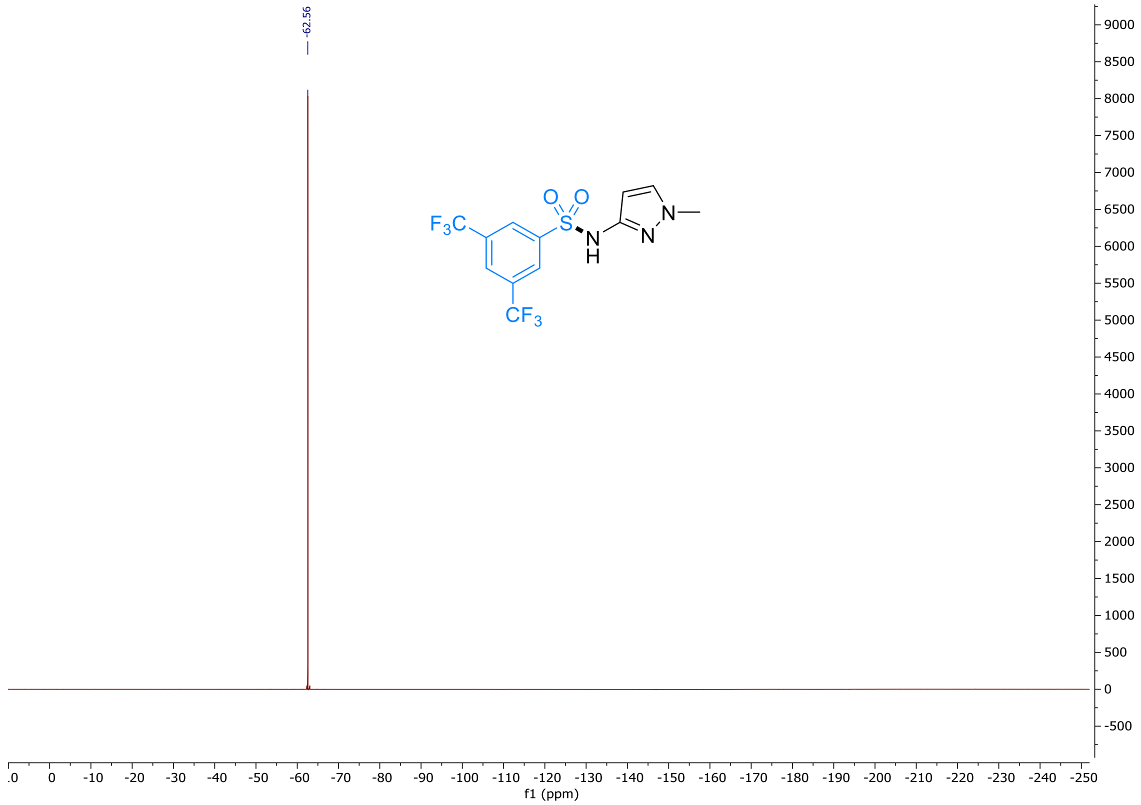

^{19}F NMR of **3x** (376 MHz, DMSO-d_6)

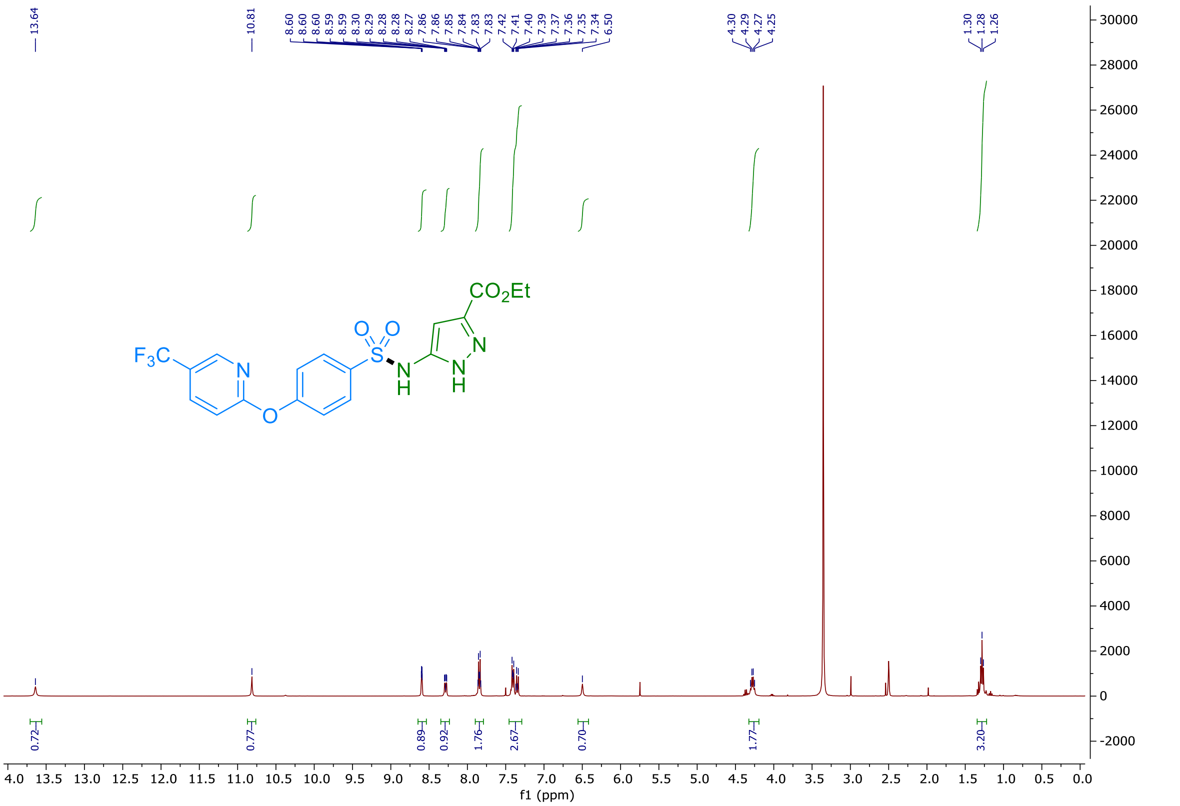

^{1}H NMR of **3y** (400 MHz, DMSO-d_6)

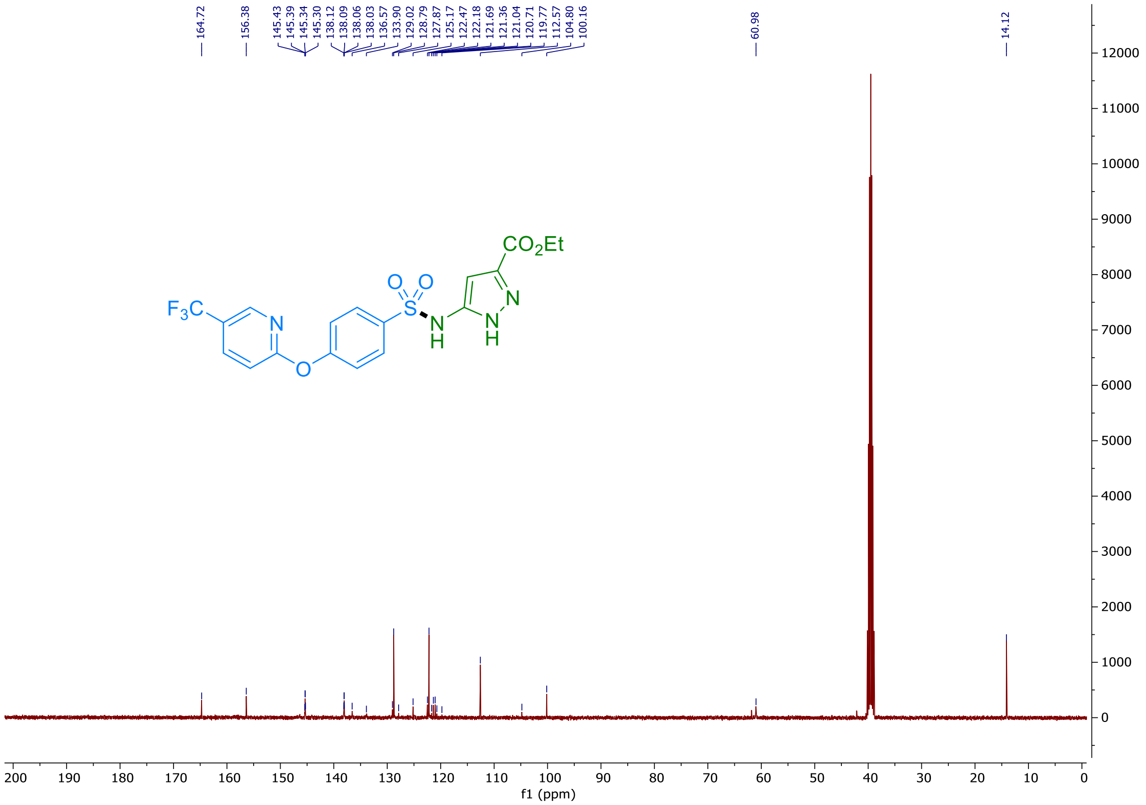

^{13}C NMR of **3y** (101 MHz, DMSO-d_6)

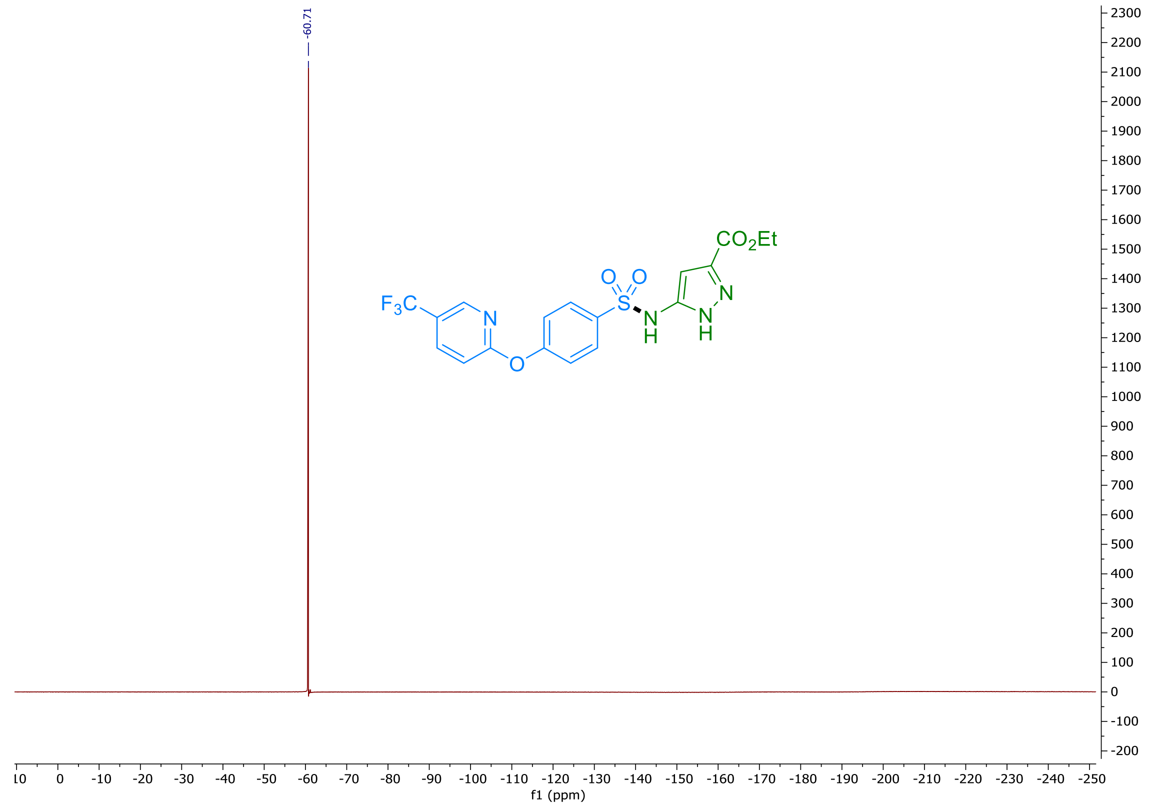

^{19}F NMR of **3y** (376 MHz, DMSO-d_6)

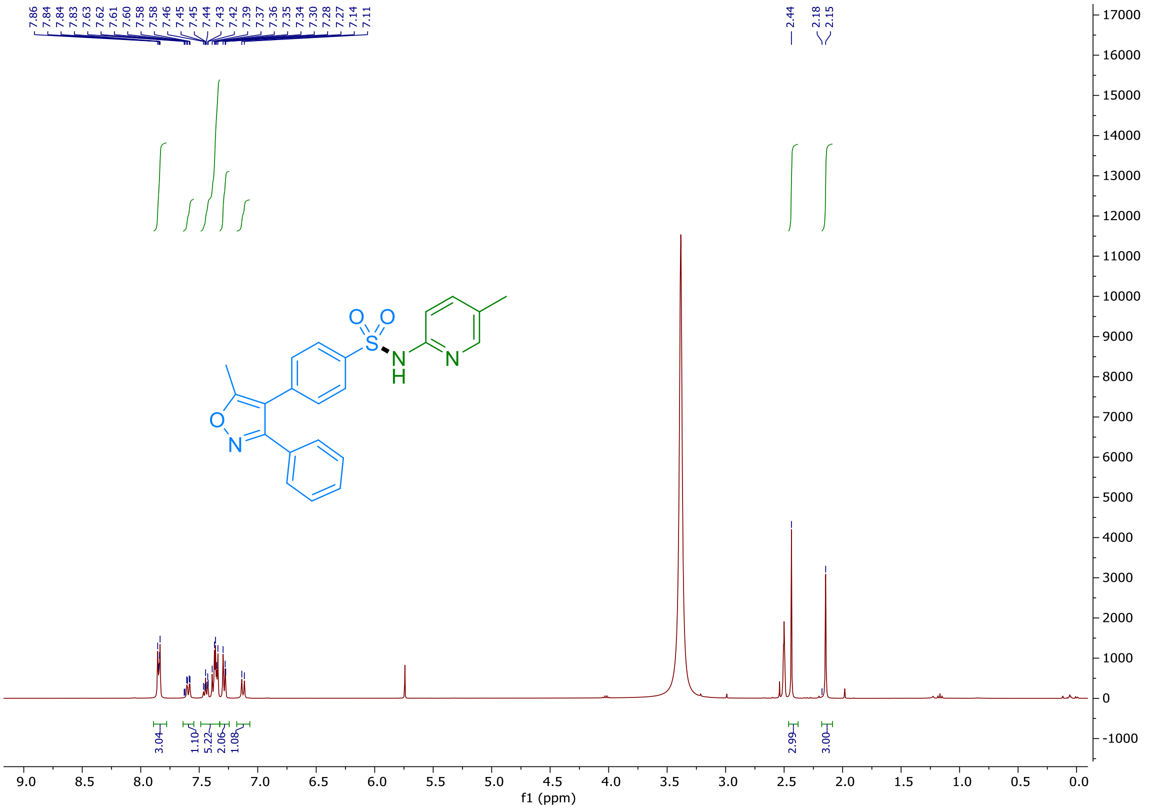

^{1}H NMR of **3z** (400 MHz, DMSO-d_6)

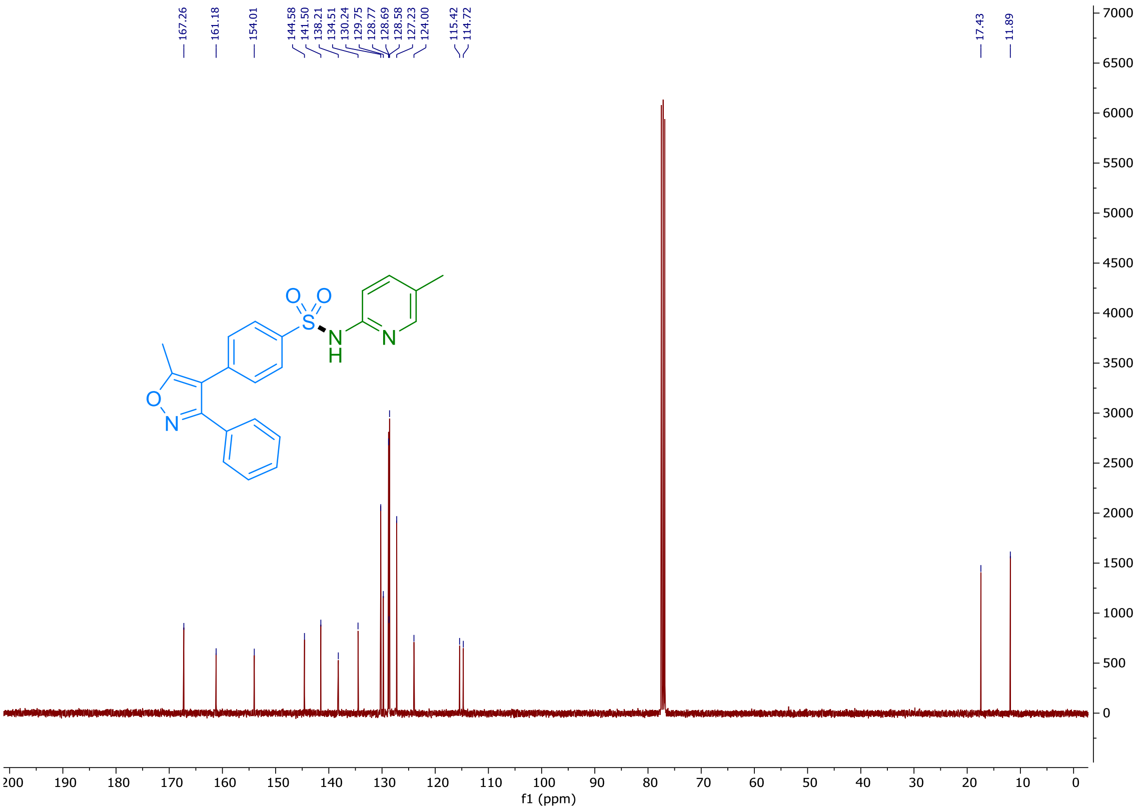

^{13}C NMR of **3z** (101 MHz, $CDCl_3$)

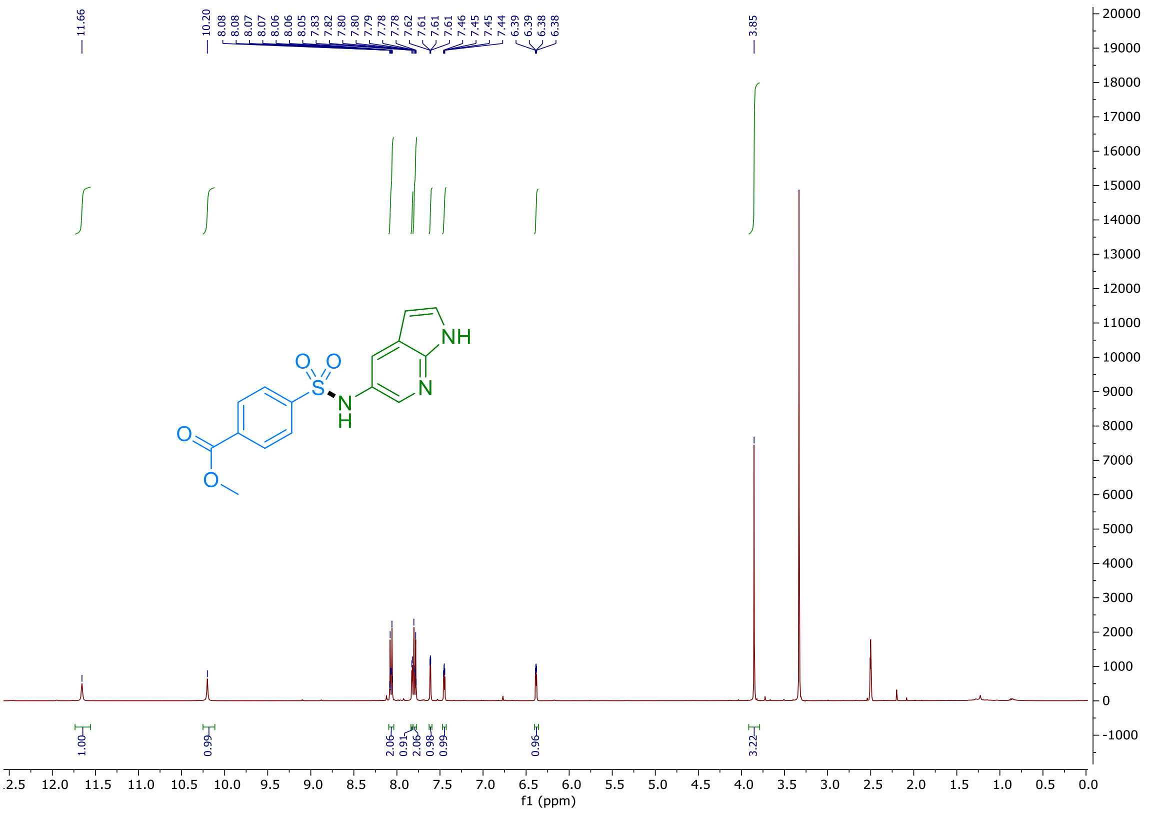

^{1}H NMR of **3aa** (400 MHz, DMSO-d_6)

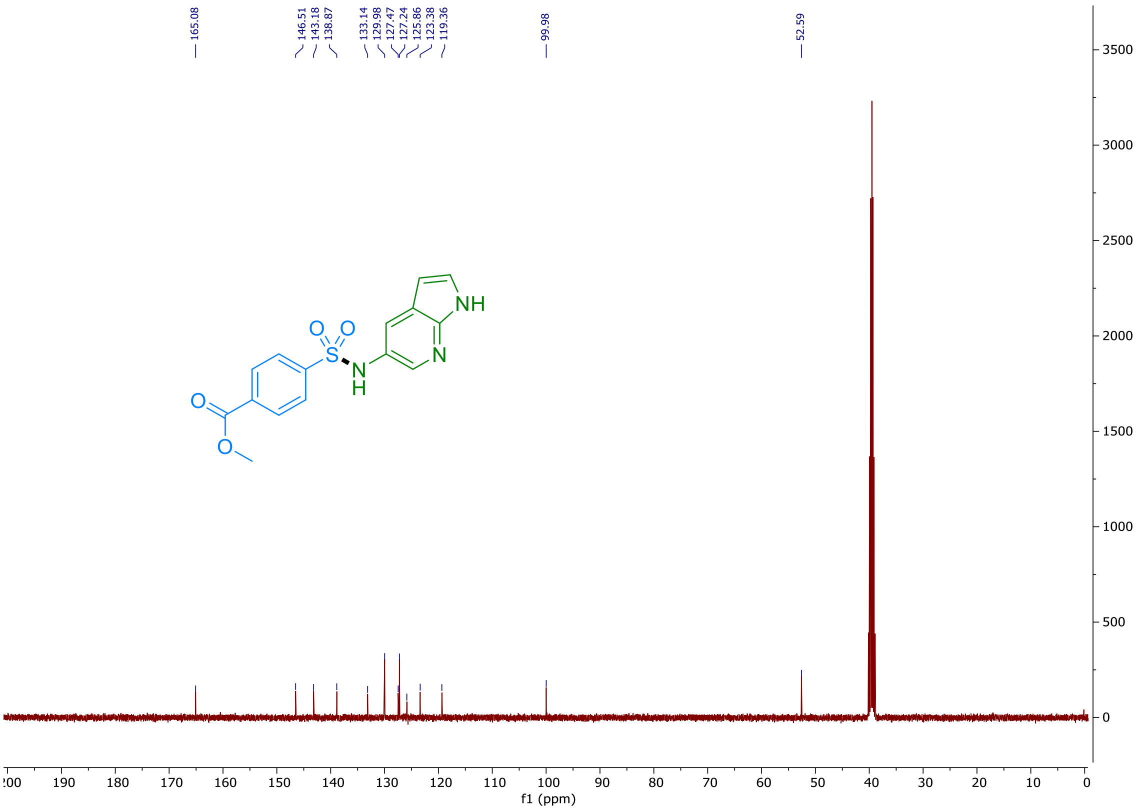

^{13}C NMR of **3aa** (101 MHz, DMSO-d_6)

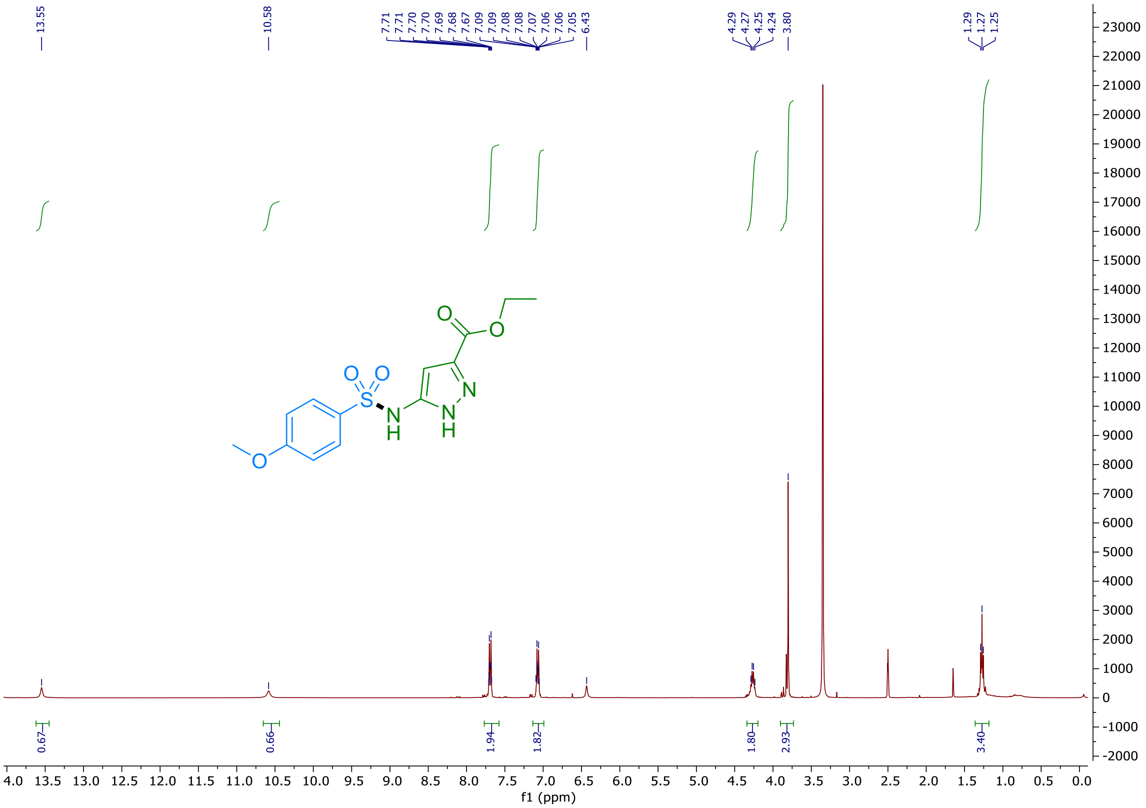

^{1}H NMR of **3ab** (400 MHz, DMSO-d_6)

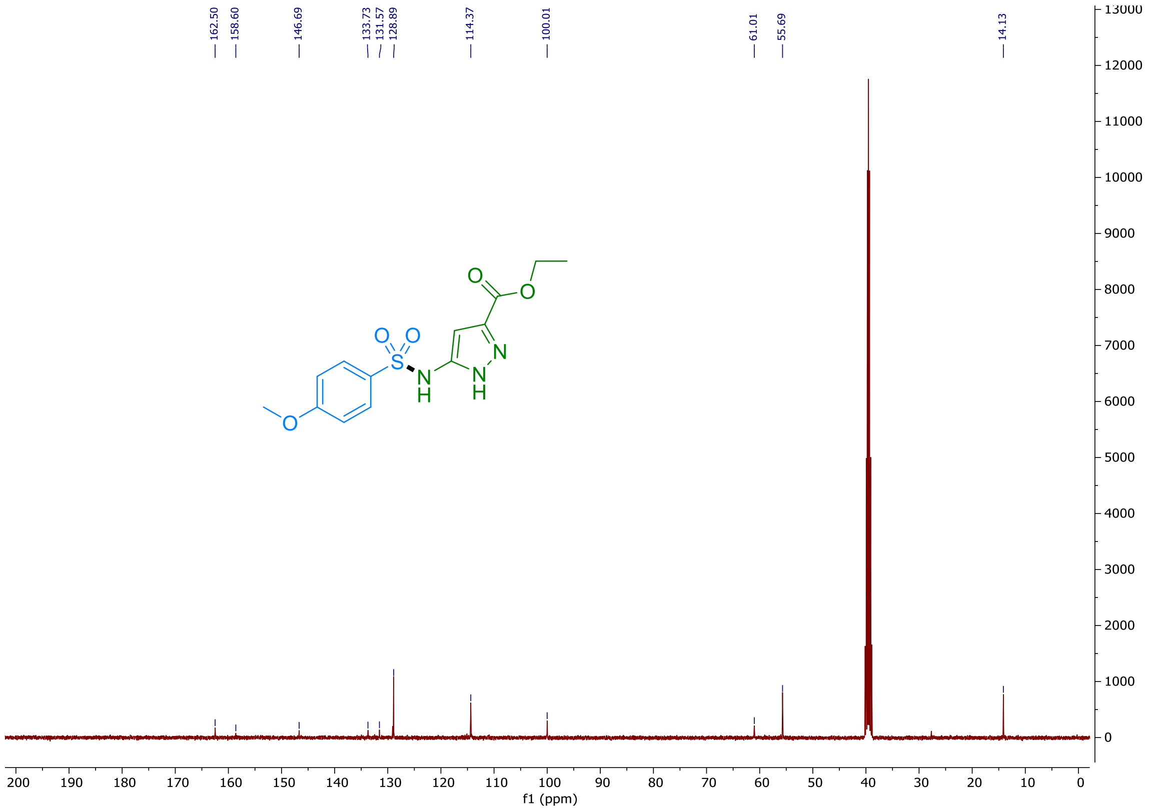

^{13}C NMR of **3ab** (101 MHz, DMSO-d_6)

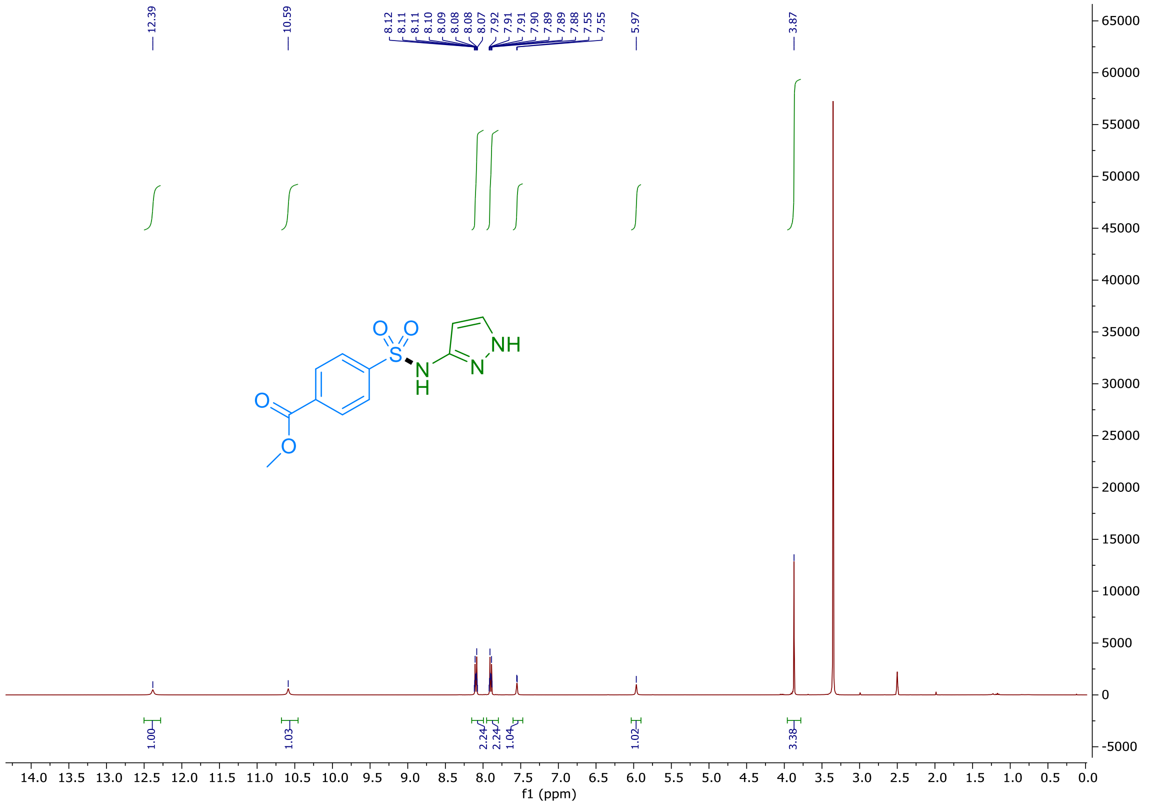

^{1}H NMR of **3ac** (400 MHz, DMSO-d_6)

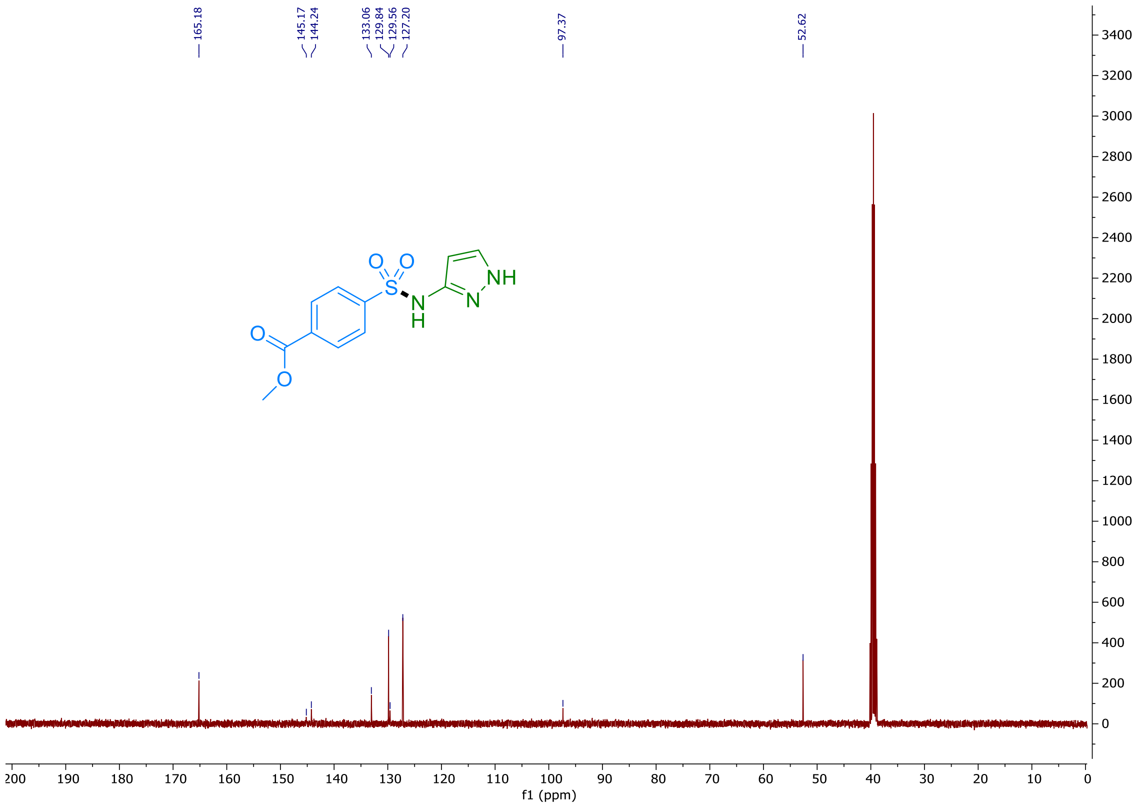

^{13}C NMR of **3ac** (101 MHz, DMSO-d_6)

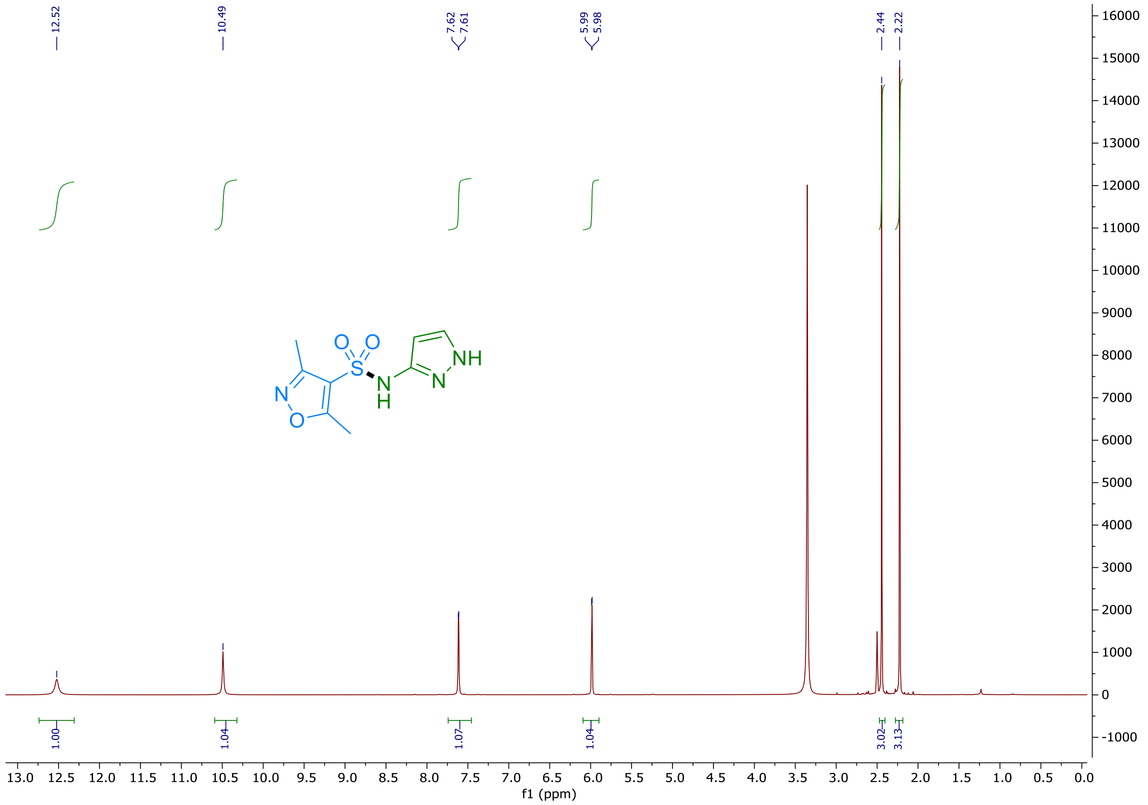

^{1}H NMR of **3ad** (400 MHz, DMSO-d_6)

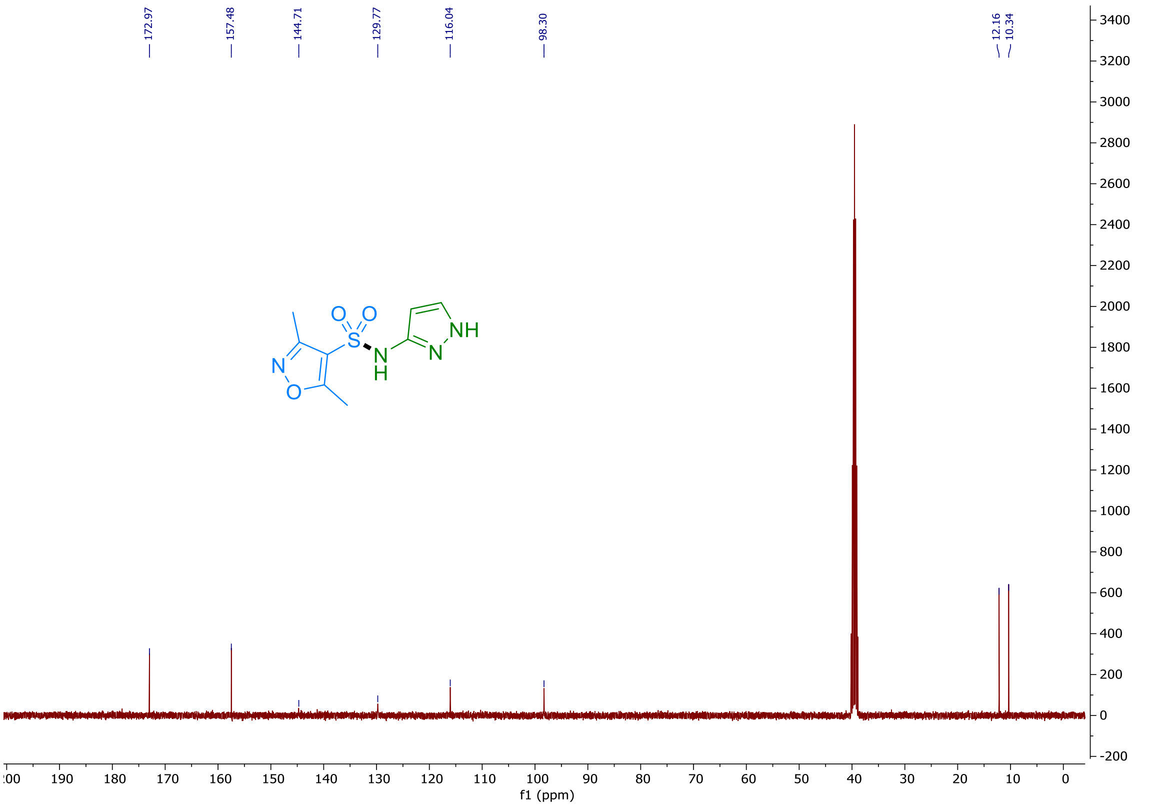

^{13}C NMR of **3ad** (101 MHz, DMSO-d_6)

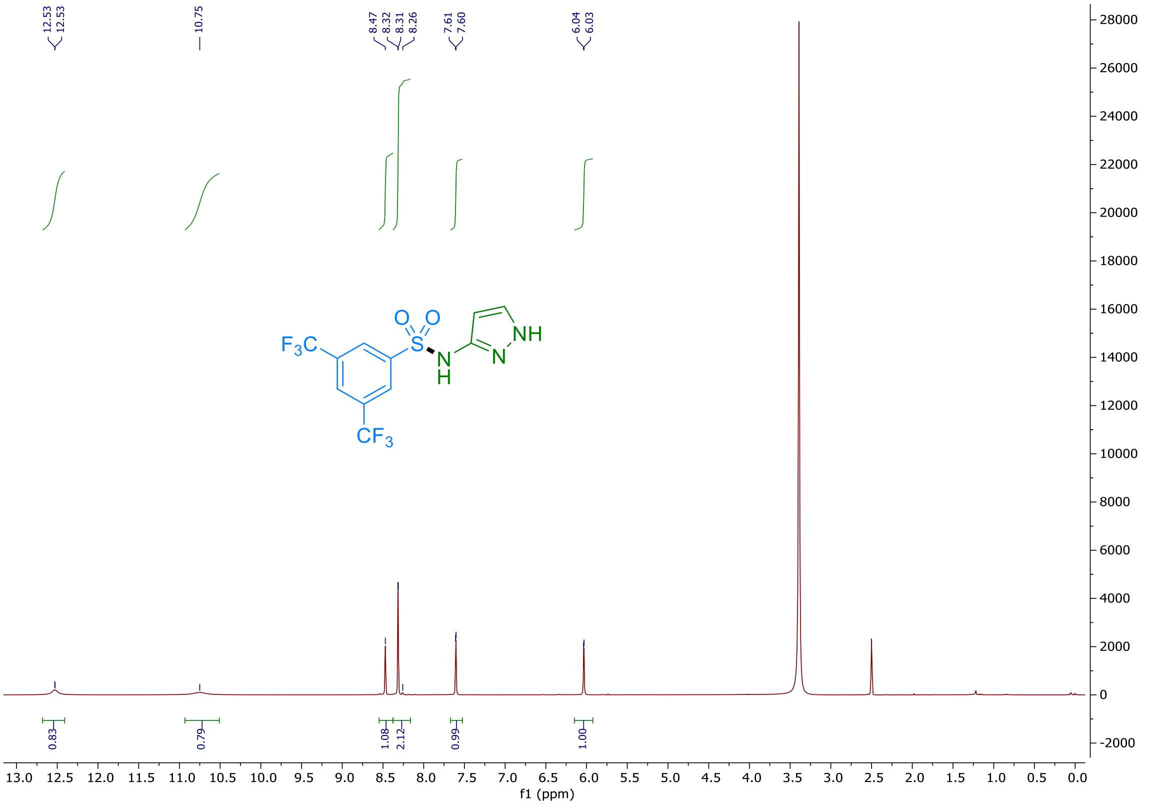

^{1}H NMR of **3ae** (400 MHz, DMSO-d_6)

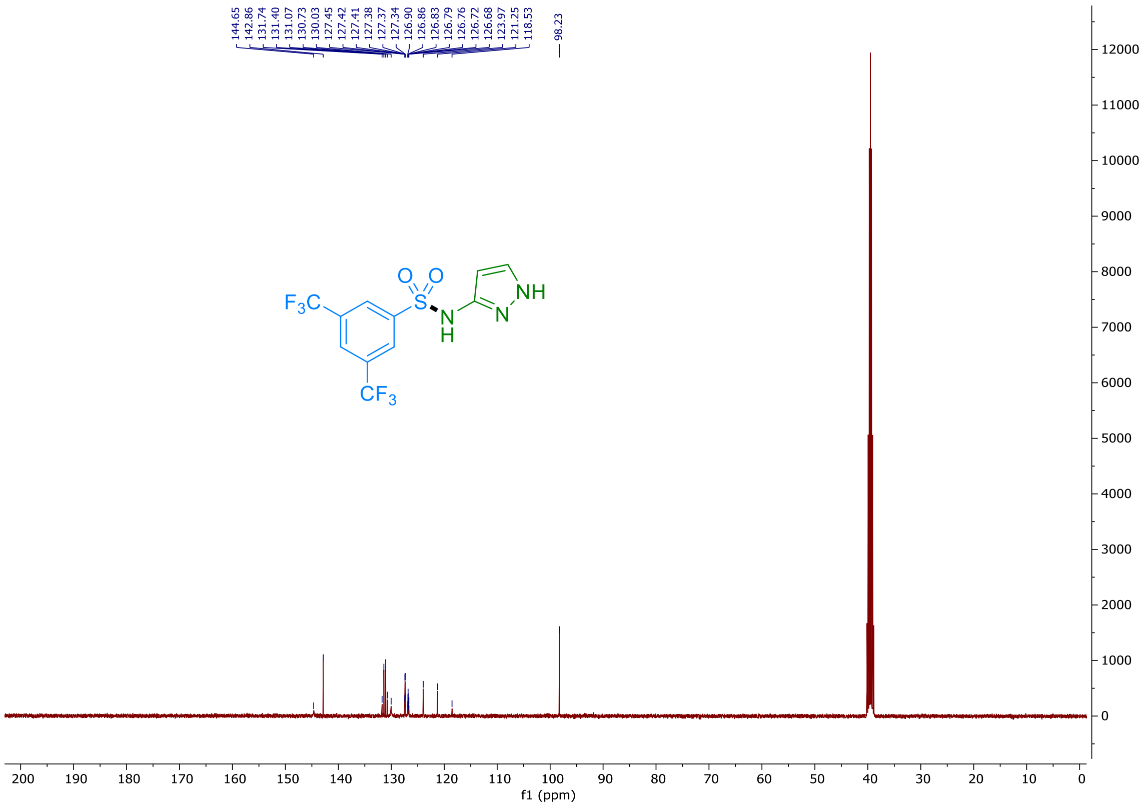

^{13}C NMR of **3ae** (101 MHz, DMSO-d_6)

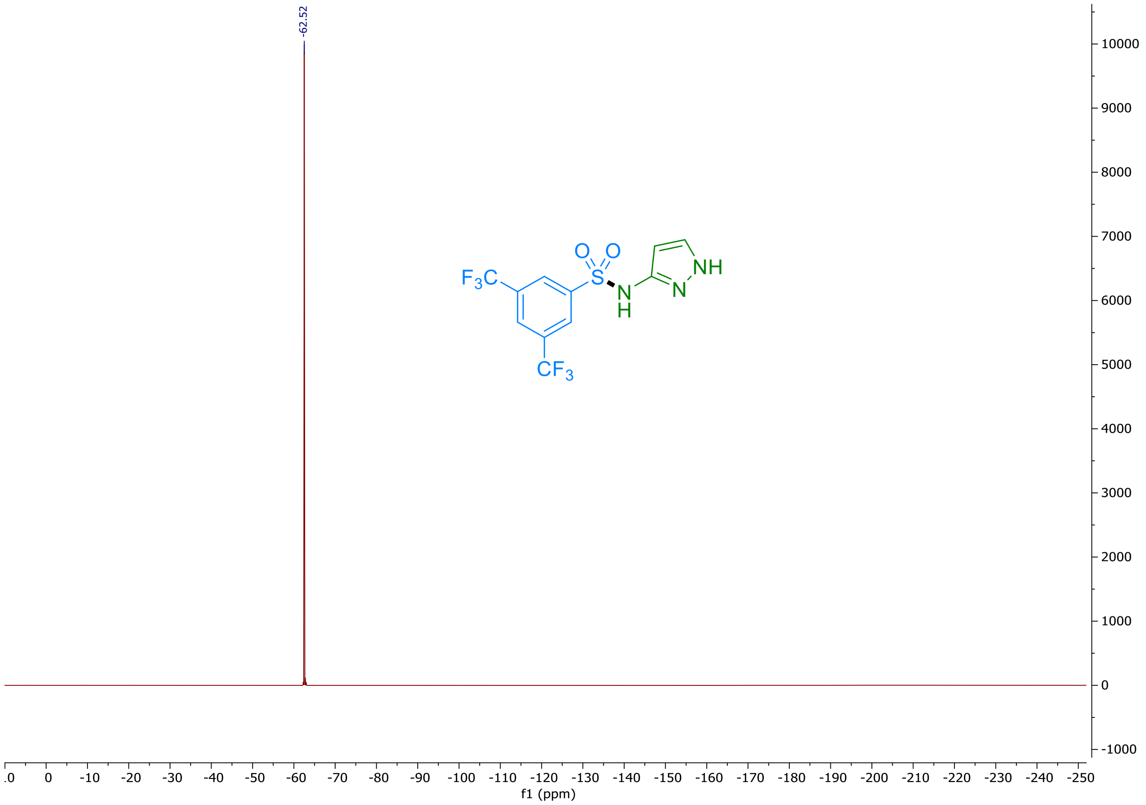

^{19}F NMR of **3ae** (376 MHz, DMSO-d_6)

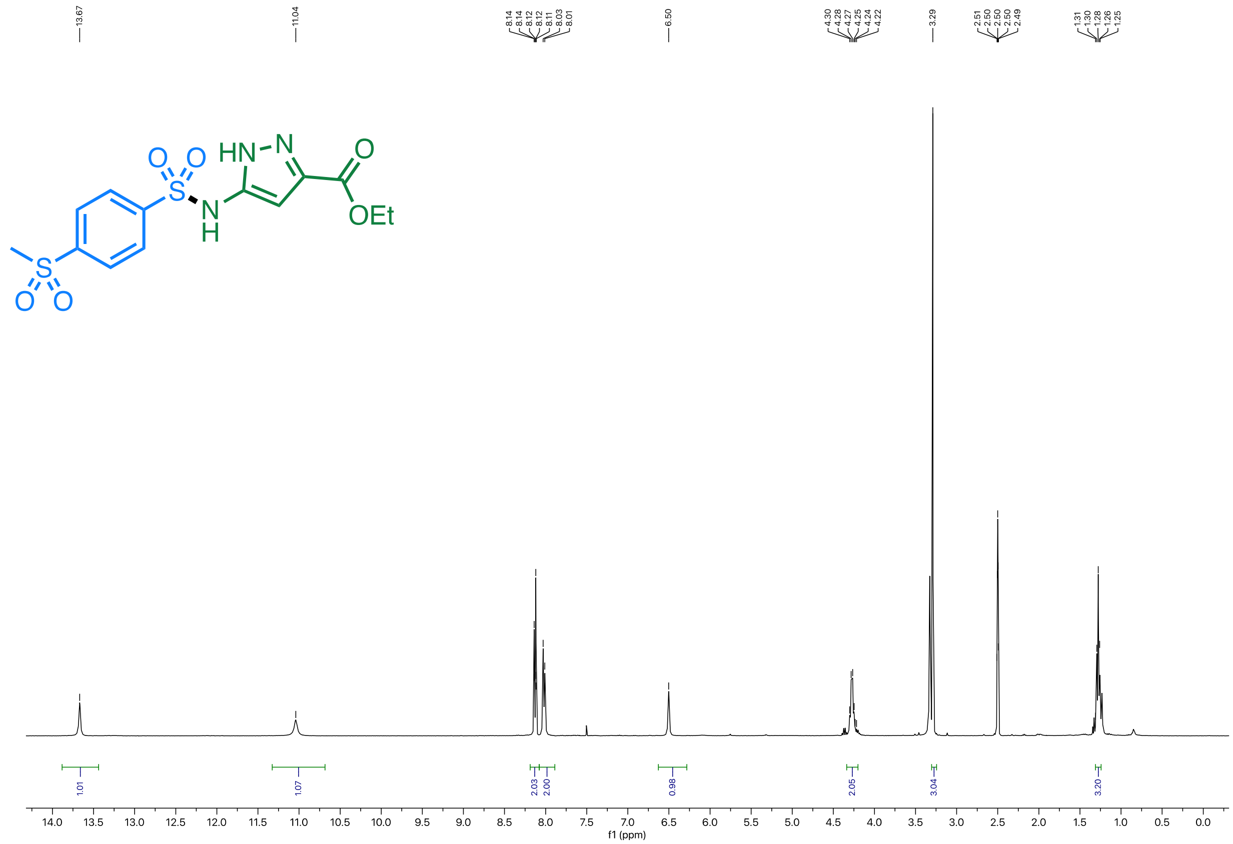

^{1}H NMR of **3ag** (400 MHz, DMSO-d_6).

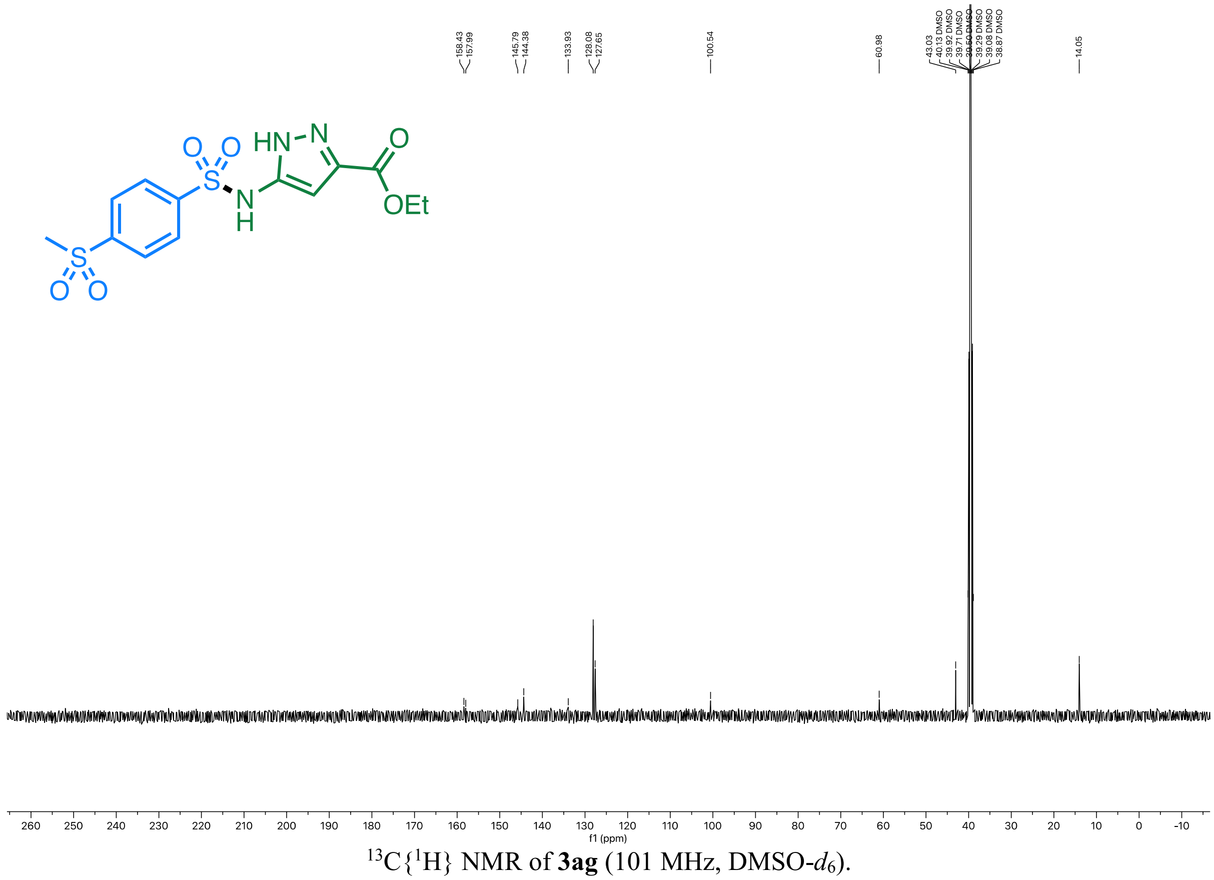

$^{13}C\{^{1}H\}$ NMR of **3ag** (101 MHz, DMSO-d_6).

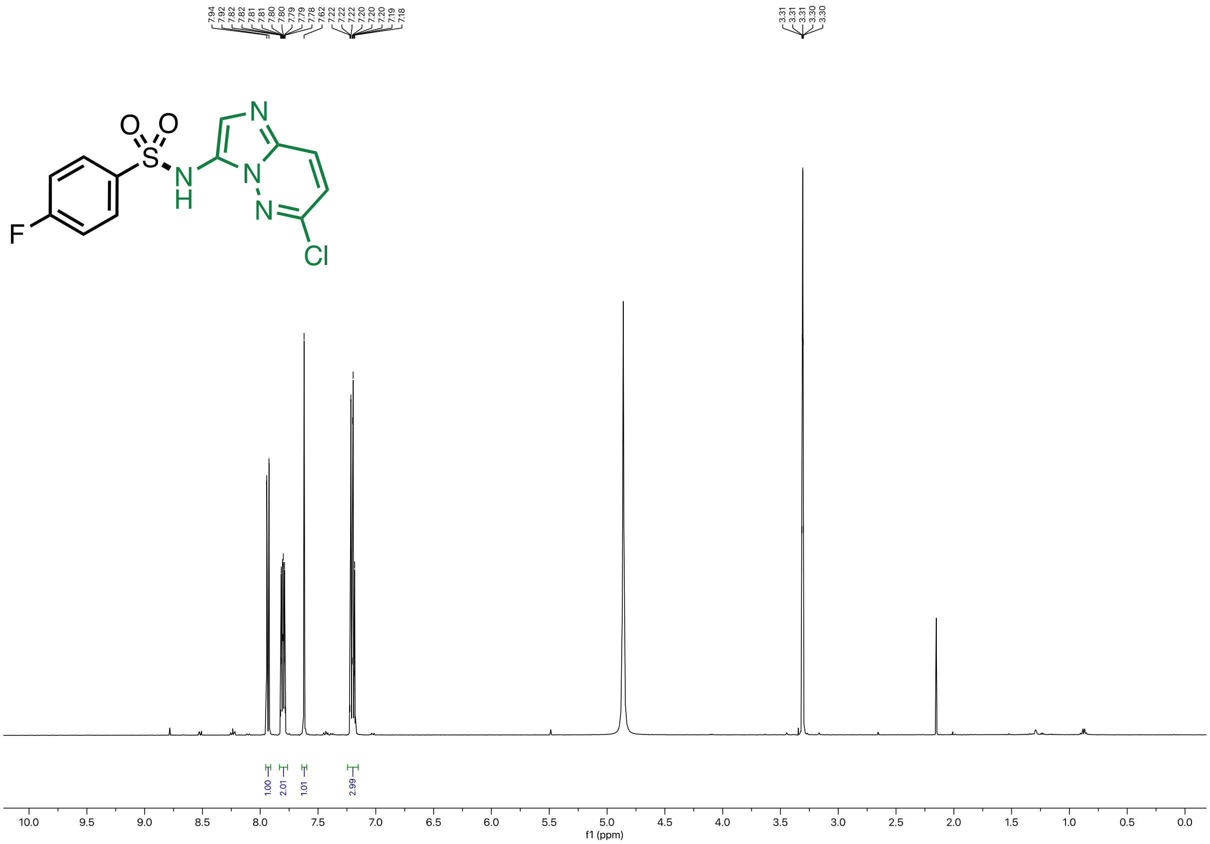

^{1}H NMR of **3ah** (500 MHz, CD_3OD).

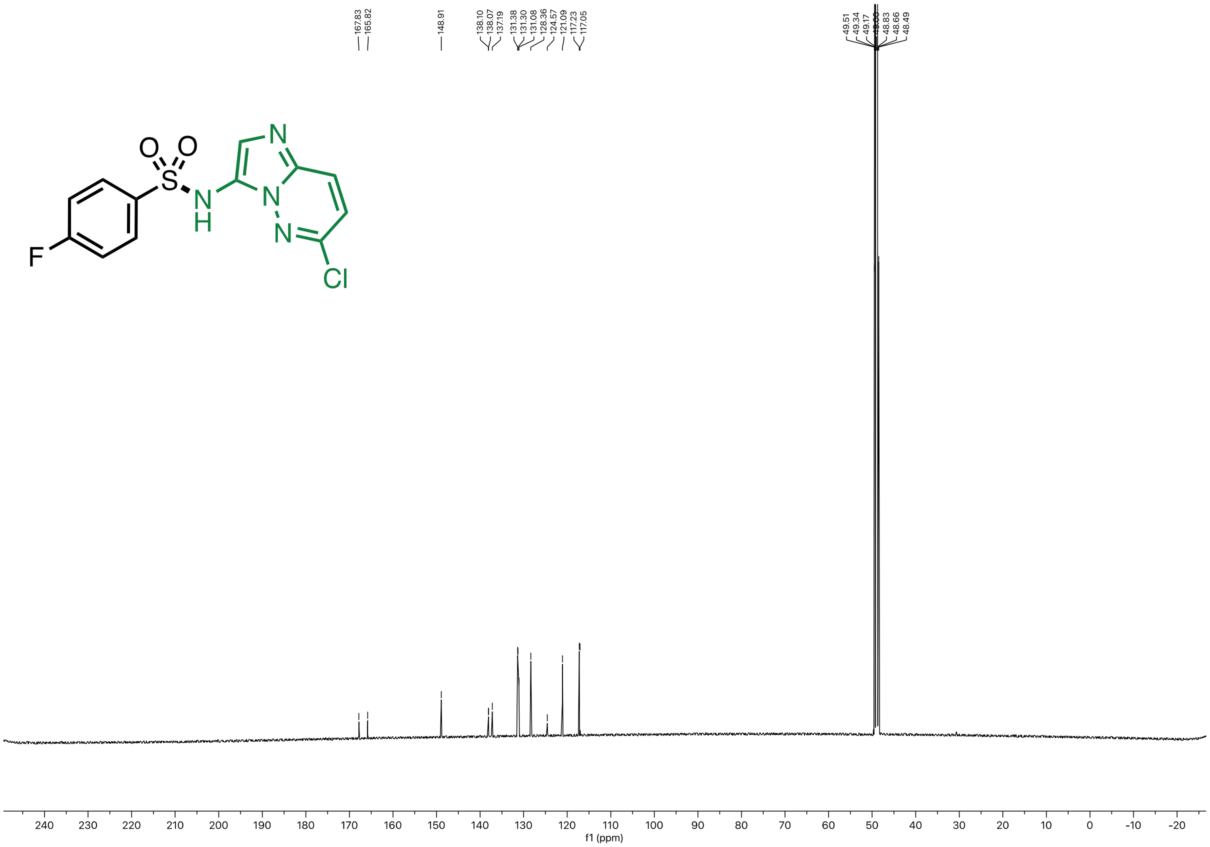

^{13}C{^{1}H} NMR of **3ah** (126 MHz, CD_3OD).

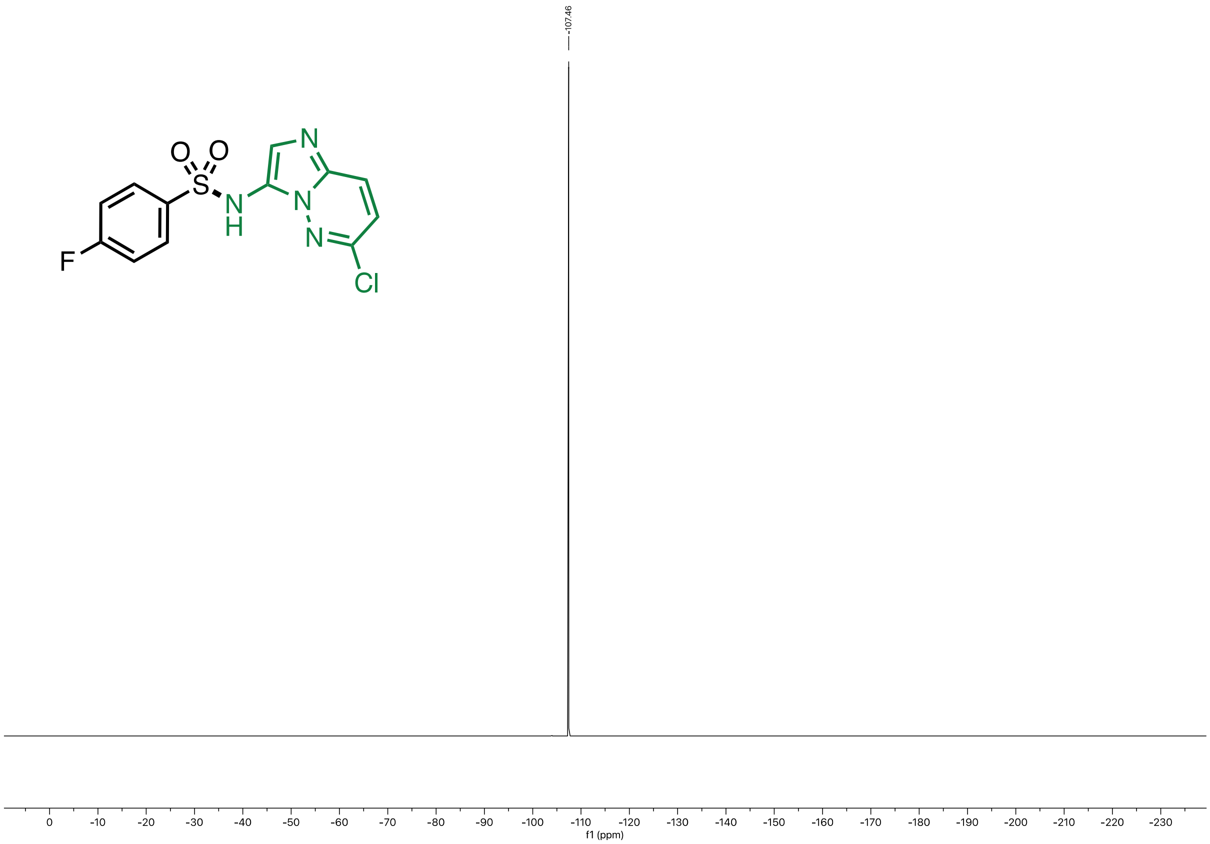

$^{19}F\{^{1}H\}$ NMR of **3ah** (376 MHz, CD_3OD).

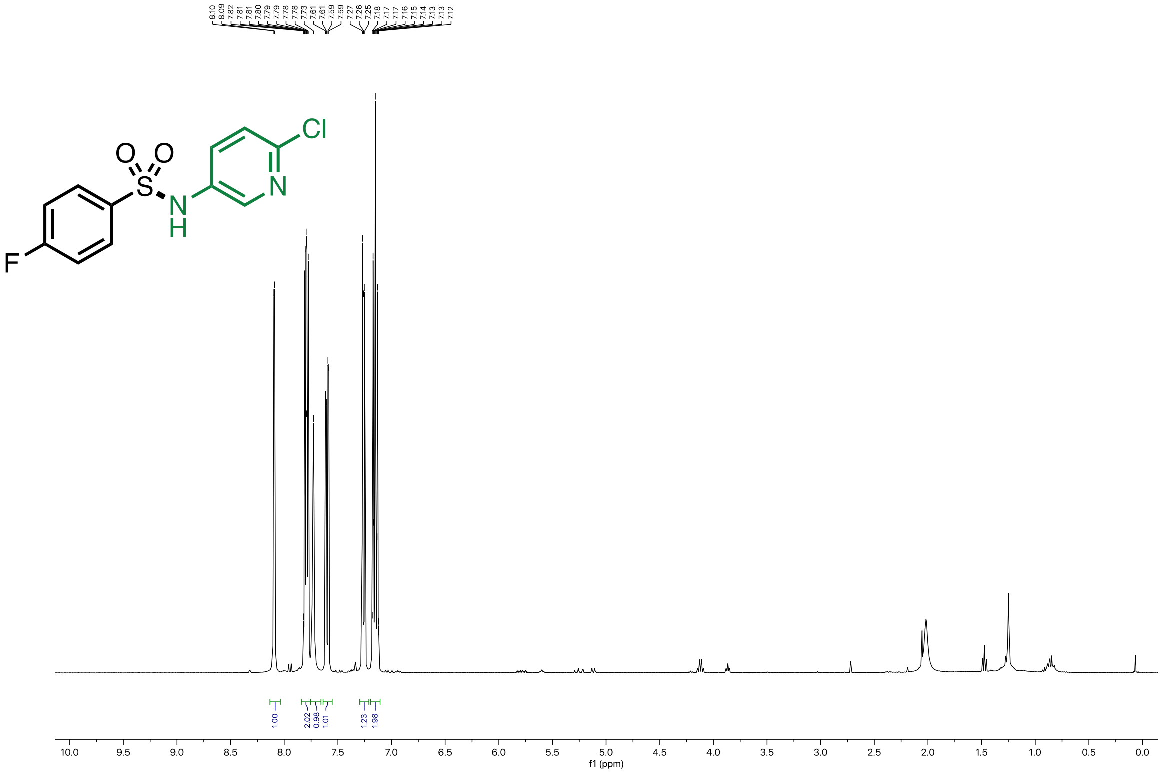

^{1}H NMR of **3ai** (400 MHz, $CDCl_3$).

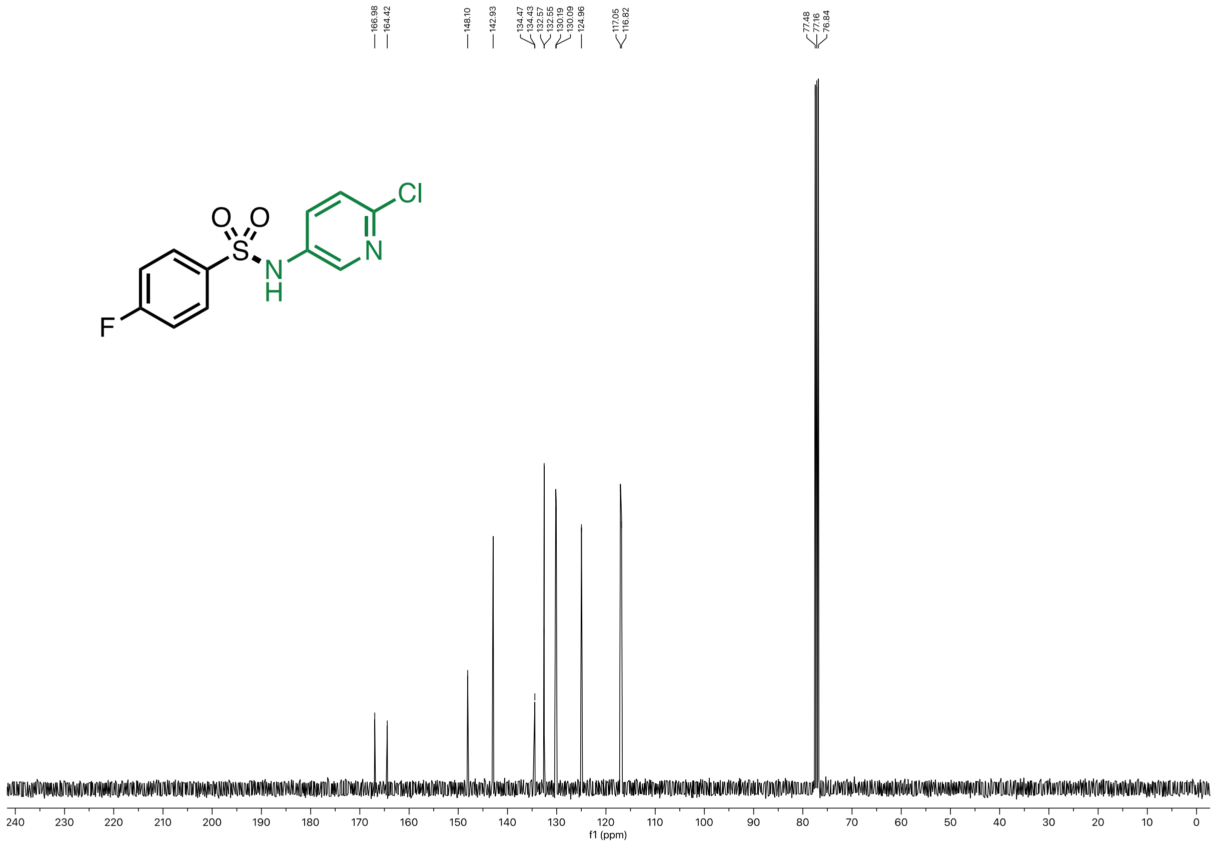

$^{13}C\{^{1}H\}$ NMR of **3ai** (101 MHz, $CDCl_3$).

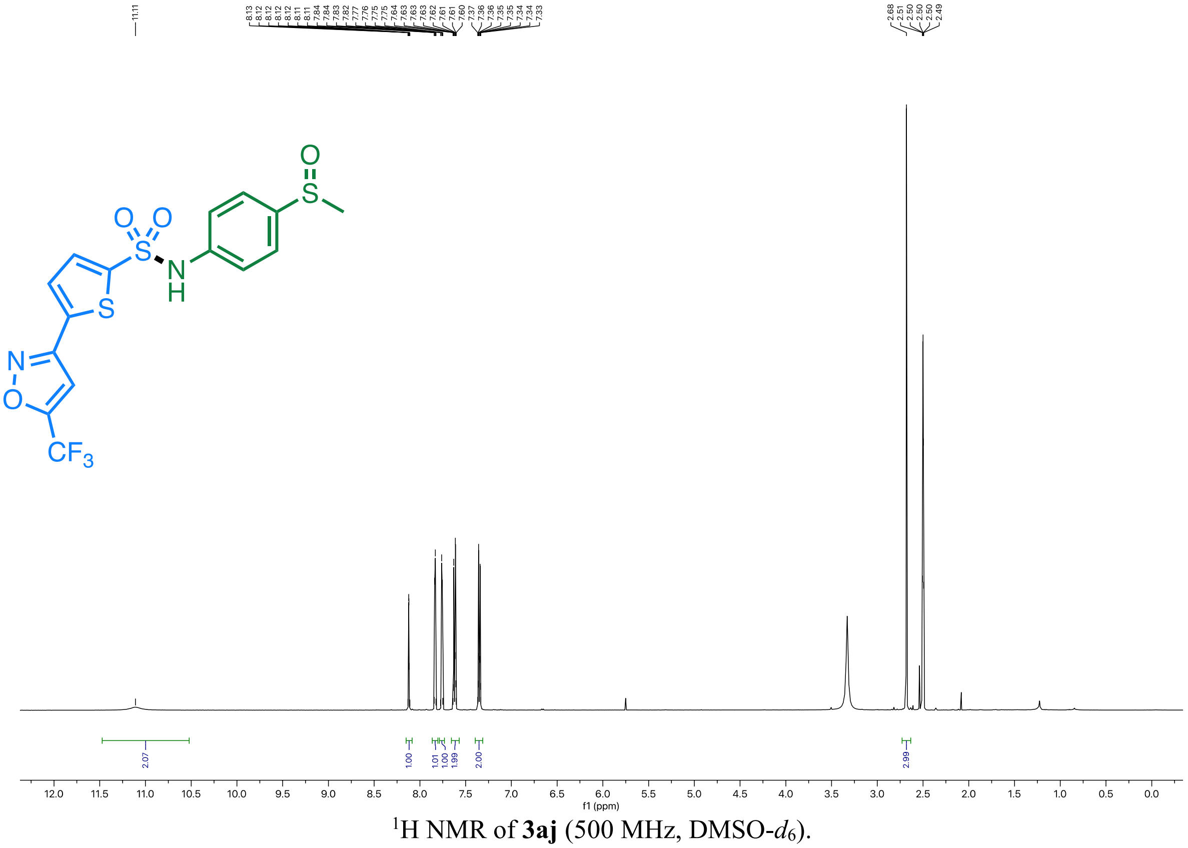

^{1}H NMR of **3aj** (500 MHz, DMSO-d_6).

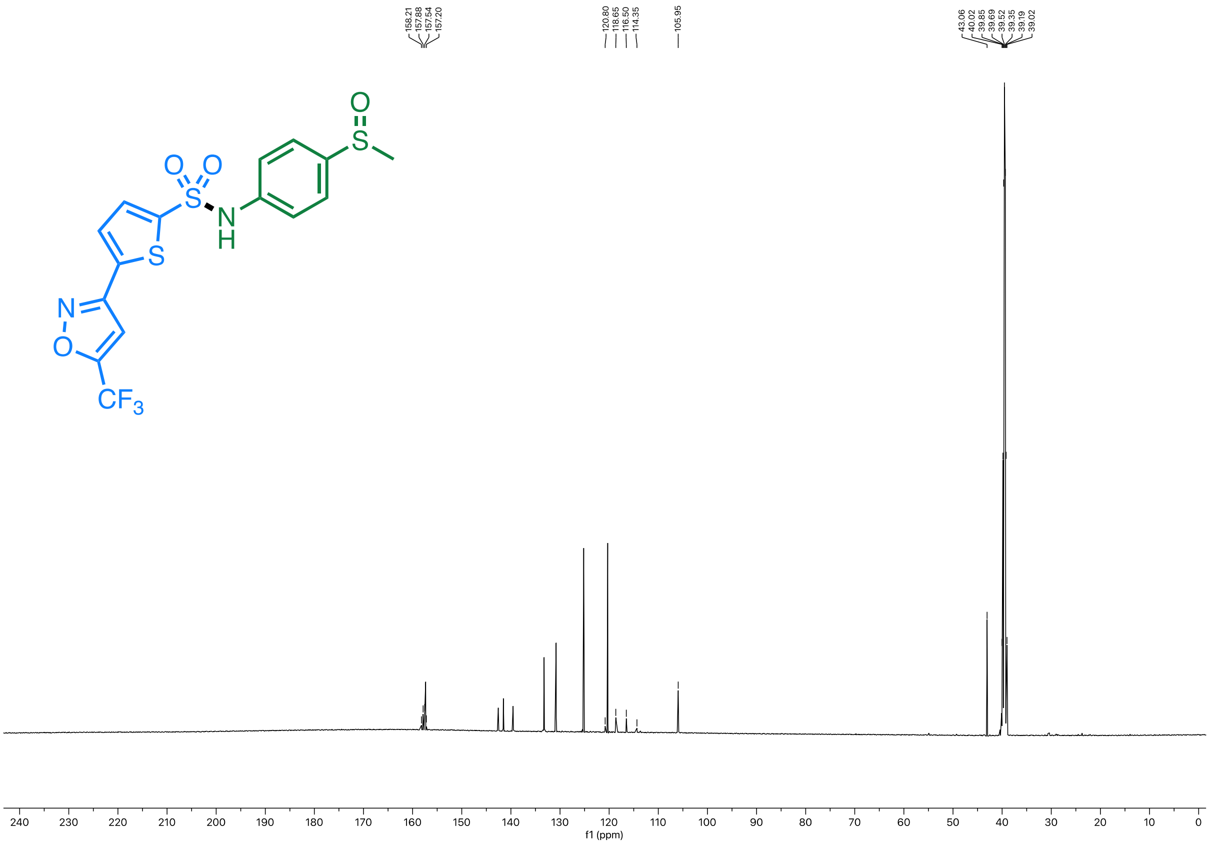

$^{13}C\{^1H\}$ NMR of **3aj** (126 MHz, DMSO-d_6).

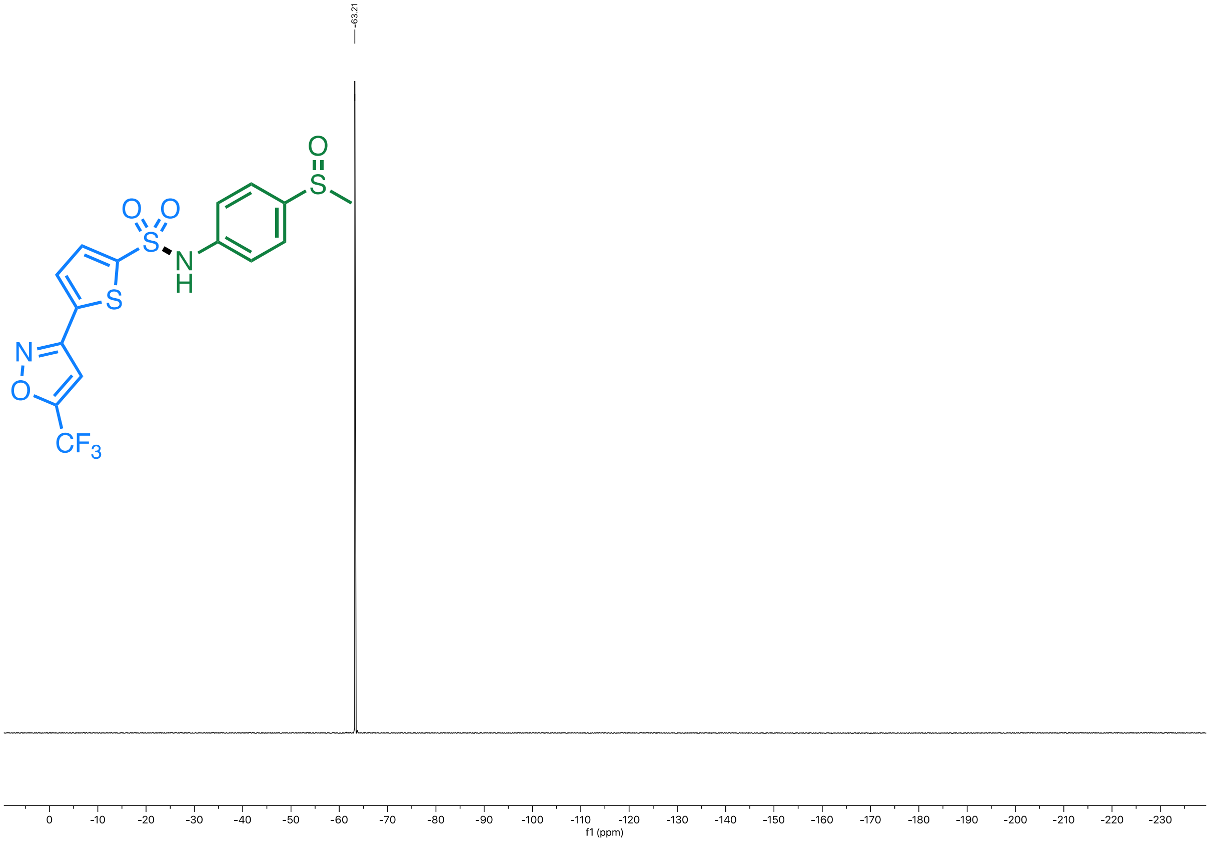

$^{19}F\{^1H\}$ NMR of **3aj** (376 MHz, DMSO-d_6).

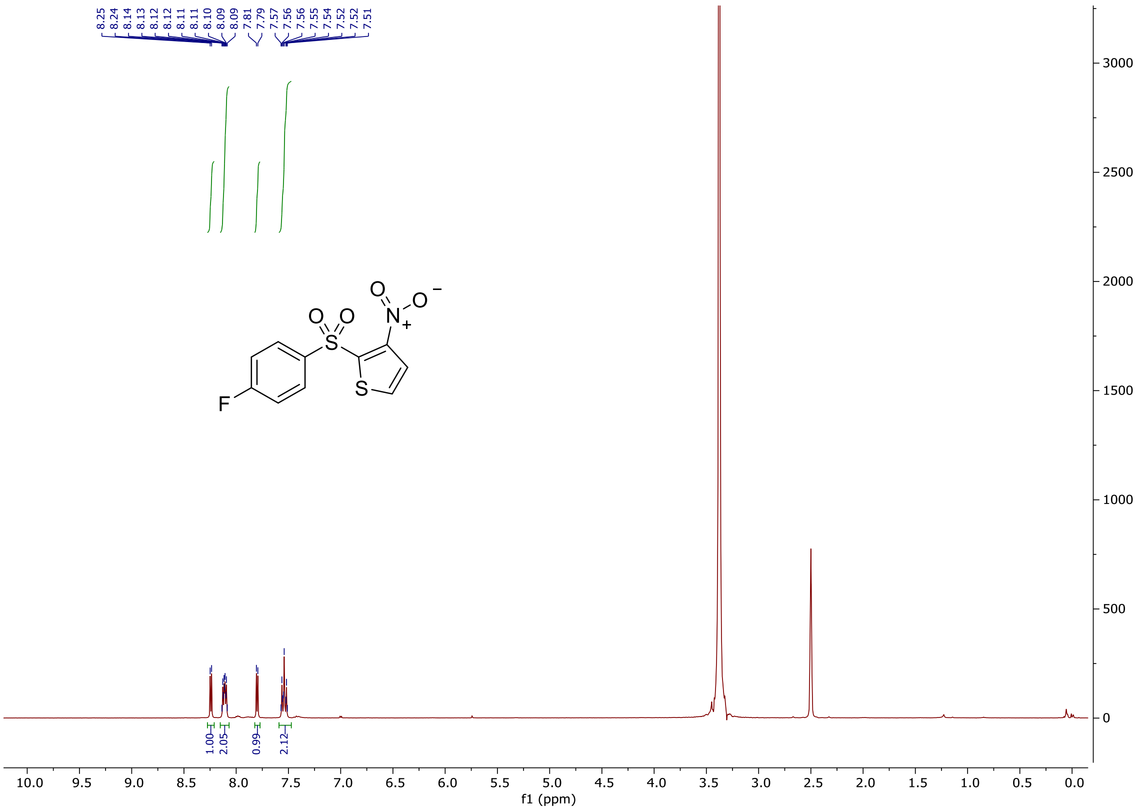

^{1}H NMR of **S1** (400 MHz, DMSO-d_6)

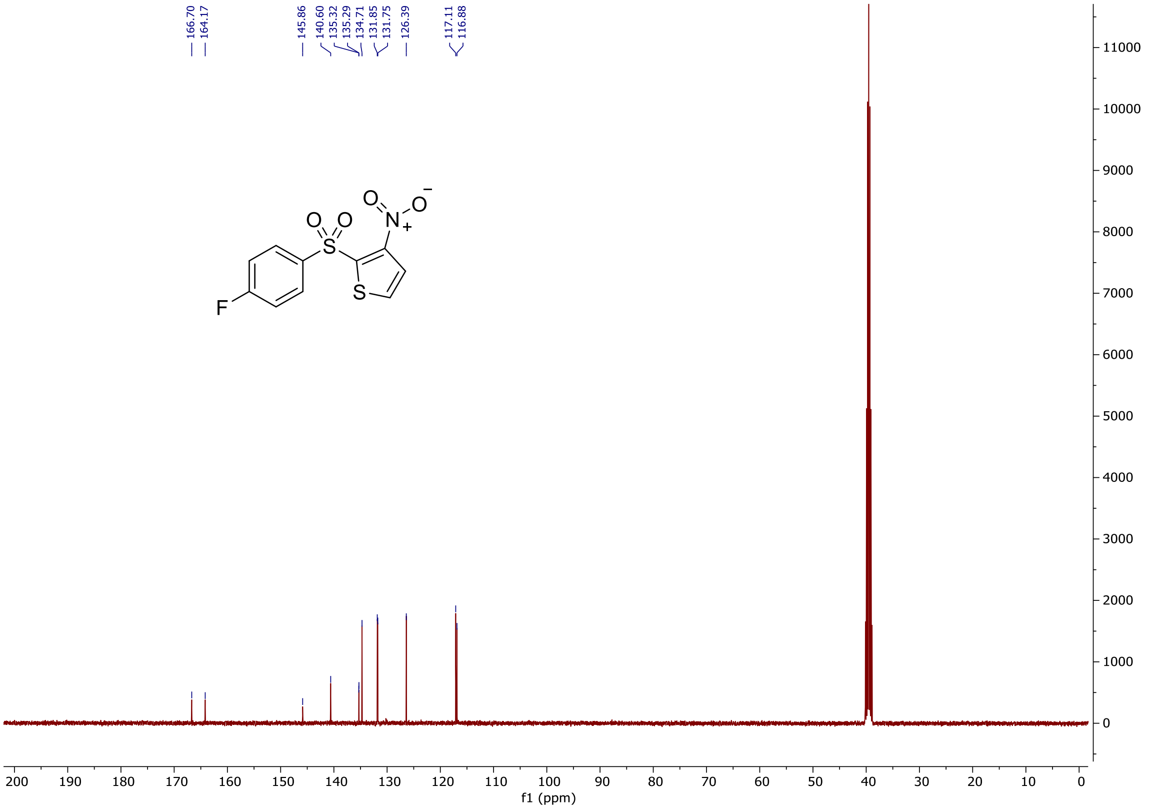

^{13}C NMR of **S1** (101 MHz, DMSO-d_6)

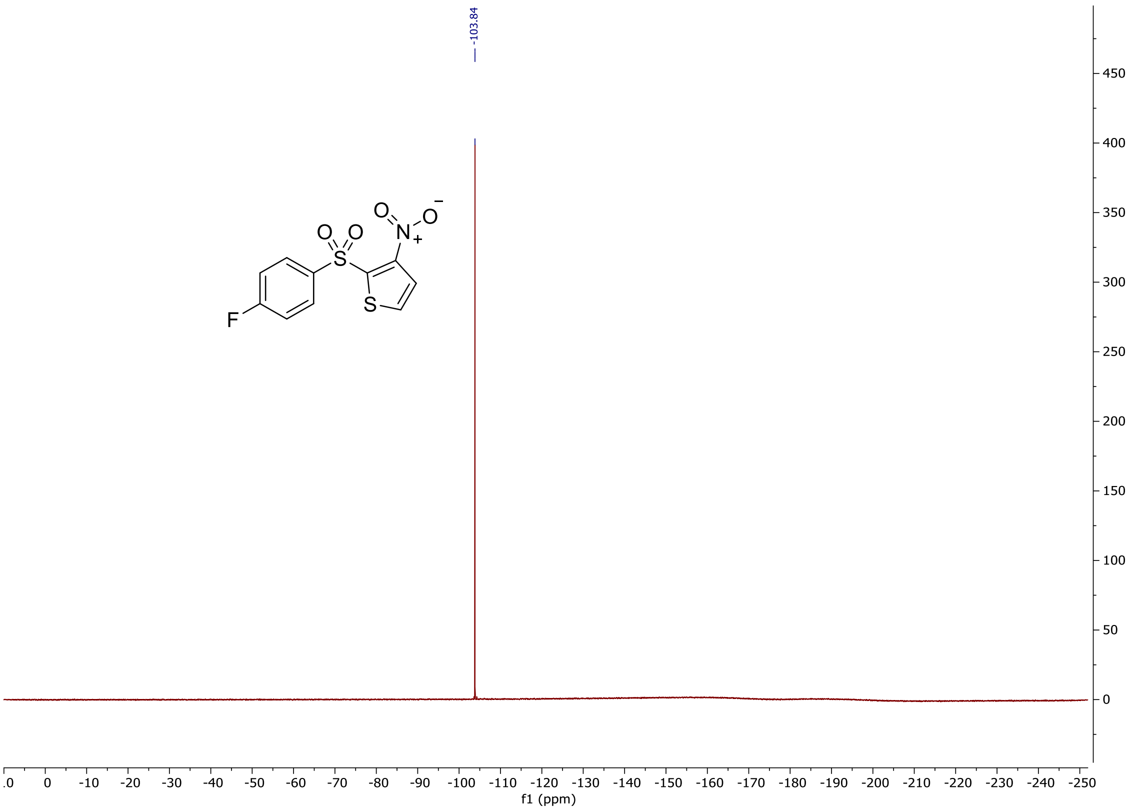

^{19}F NMR of **S1** (376 MHz, DMSO-d_6)

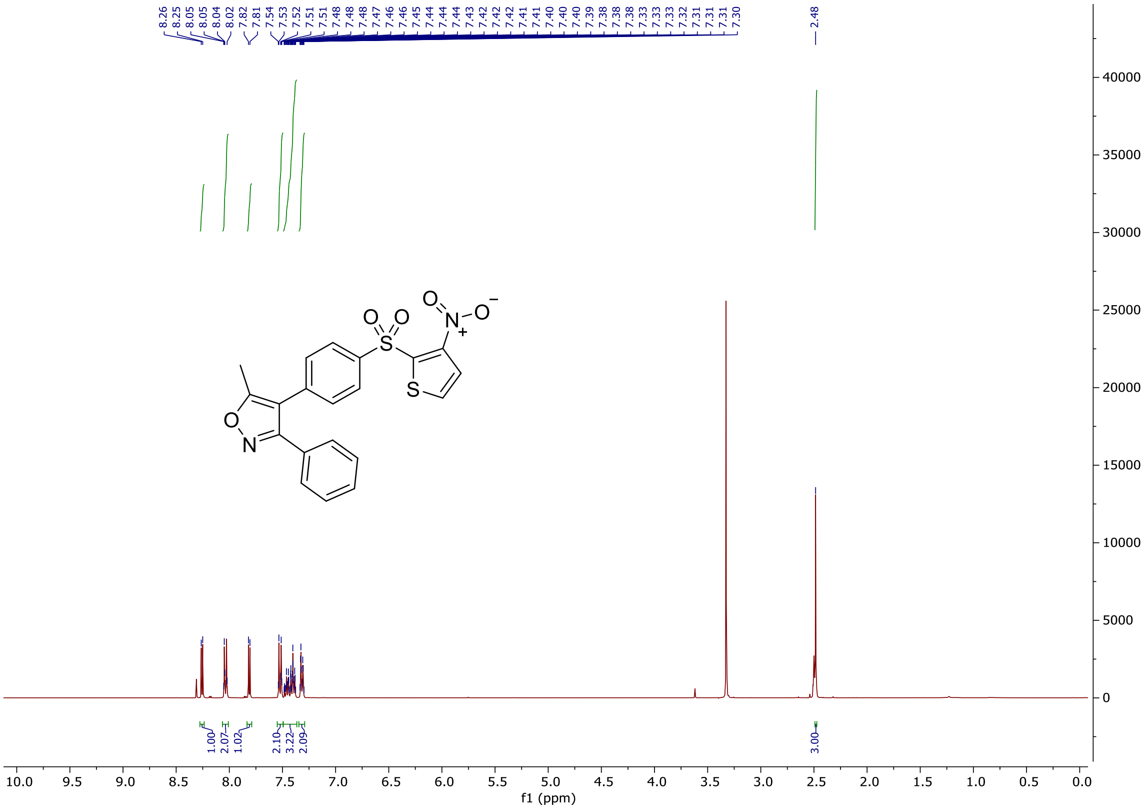

^{1}H NMR of **S2** (400 MHz, DMSO-d_6)

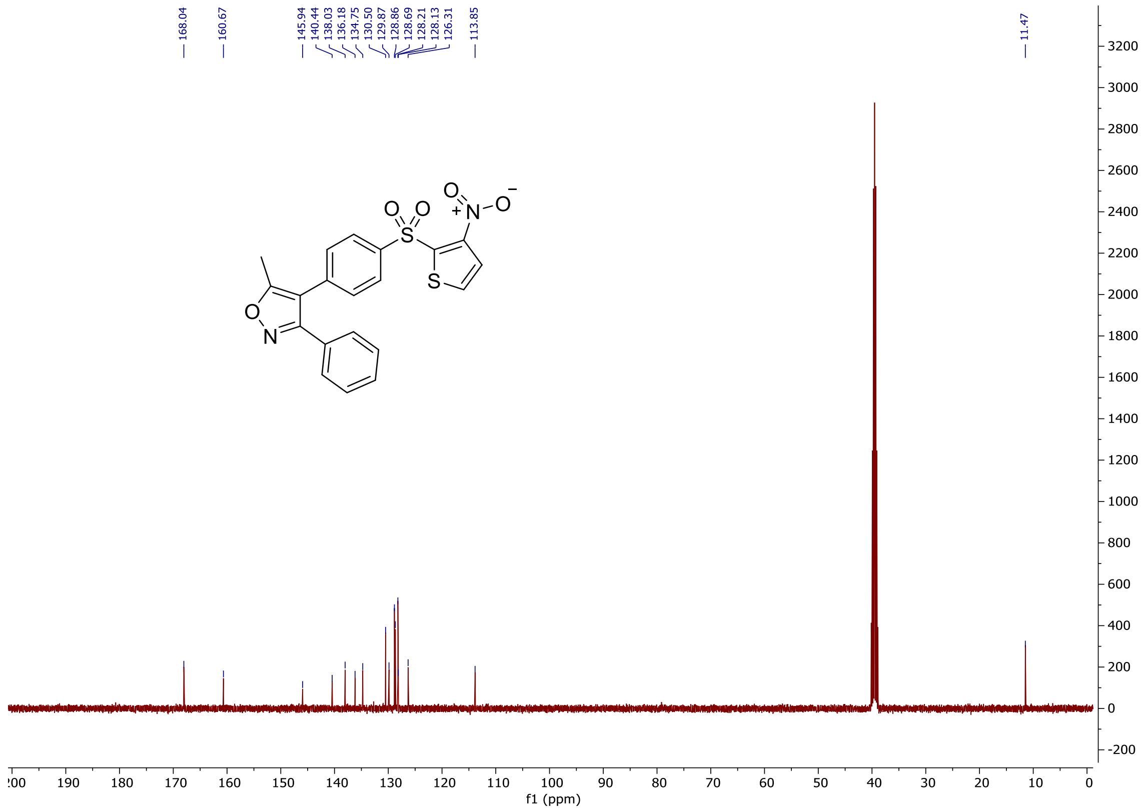

^{13}C NMR of **S2** (101 MHz, DMSO-d_6)
